# Strategic modulation of heterocyclic donors in binaphthyl acrylonitrile-based hole transport materials for perovskite solar cells: a DFT and machine learning investigation

**DOI:** 10.1039/d6ra04301a

**Published:** 2026-08-11

**Authors:** Iqra Shafiq, Asma Irshad, Muqadas Javed, Samia Majeed, Muhammad Adnan Asghar, Nadia Ayari, Ke Chen

**Affiliations:** a Institute of Chemistry, Khwaja Fareed University of Engineering & Information Technology Rahim Yar Khan 64200 Pakistan; b Department of Chemistry, Division of Science and Technology, University of Education Lahore 54000 Pakistan adnan.muhammad@ue.edu.pk; c Department of Physics, College of Science, Northern Border University Arar Saudi Arabia; d Department of Infectious Diseases, The Affiliated Hospital of Southwest Medical University Luzhou 646000 China chen_ke@swmu.edu.cn

## Abstract

Hole transport materials (HTMs) play a crucial role in perovskite solar cells (PSCs) by facilitating efficient charge extraction, suppressing interfacial recombination, and enhancing device stability. Here, a series of binaphthyl acrylonitrile-based organic chromophores (BNPD1–BNPD5) with heterocyclic donor moieties at their terminals were designed through molecular engineering for utilization as HTMs. The influence of the heterocyclic donor moieties on the optoelectronic properties of binaphthyl acrylonitrile-based derivatives was investigated through DFT/TDDFT methodologies at the M06/6-311G(d,p) level. The current study used CatBoost to predict the *E*_gap_ of binaphthyl acrylonitrile-based derivatives to complement the DFT/TD-DFT results. CatBoost achieved high accuracy (training *R*^2^ = 0.999 and test *R*^2^ = 0.8896) and demonstrated high predictability. SHAP enhanced the interpretability and trustworthiness of the model by providing accurate predictions and interpretable insights into the relevance of molecular descriptors. Various investigations, such as UV-visible spectroscopy, hole–electron transport, frontier molecular orbital (FMO), density of states (DOS) and transition density matrix (TDM) analyses, were conducted on the entitled chromophores. The HOMO/LUMO energy gaps of BNPD1–BNPD5 were found to be within the range of 4.050–2.683 eV, with the absorption maxima observed in the UV region (434.179–393.826 nm). According to the DOS and TDM analyses, good charge transfer occurred from the terminal donors to the central acceptor in all the derivatives. All the investigated chromophores exhibited relatively low exciton binding energies, following a decreasing trend (in eV): BNPD4 (0.648) > BNPD5 (0.621) > BNPR (0.611) > BNPD2 (0.340) > BNPD3 (0.335) > BNPD1 (0.069). A benchmark study using Spiro-OMeTAD (a reference HTM) illustrated close agreement, which also suggested that these materials can act as reasonable HTMs for perovskite solar cells.

## Abstract 2

Binaphthyl acrylonitrile-based chromophores (BNPD1–BNPD5) illustrated good optoelectronic properties can be used for HTMs.

## Graphical abstract

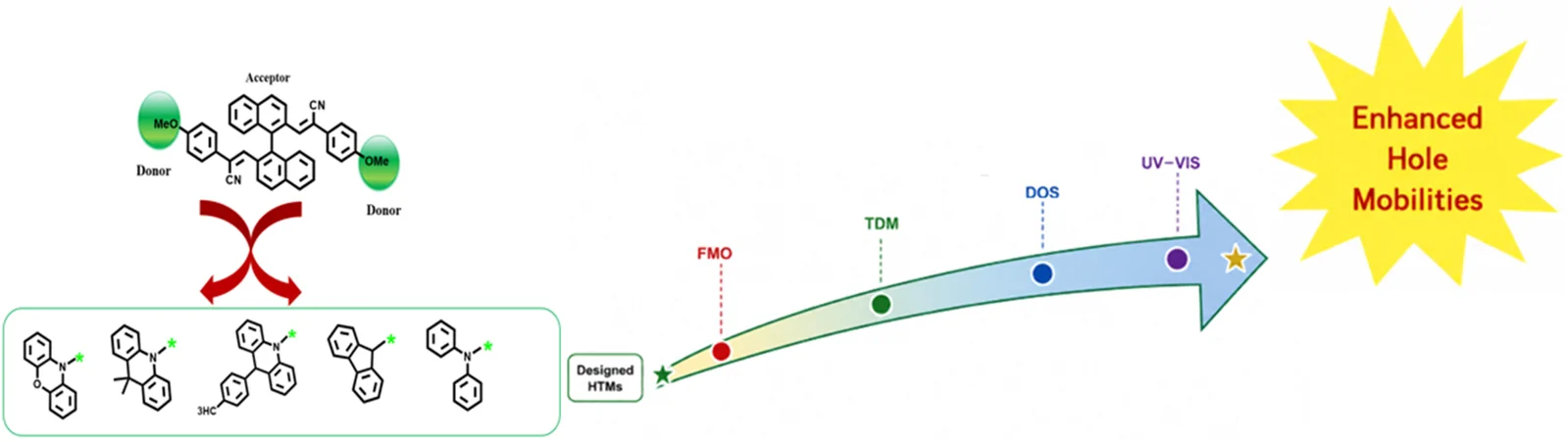

## Introduction

In the modern era, the global energy crisis remains a critical challenge, driven by escalating consumption rates and a heavy reliance on finite, non-renewable energy sources. Solar energy represents a promising solution to the increasing global energy crisis due to the widespread availability, non-exhaustible nature, and non-polluting characteristics of sunlight.^1,2^ In the last few years, organic solar cells (OSCs) have gained interest among various solar cell technologies due to their adaptable structure, inherent flexibility, lightweight design, straightforward fabrication processes, cost-effectiveness, and semi-transparency.^3–8^ Despite their potential, their practical application is constrained by inherent limitations, including a wide band gap, low *V*_oc_, high production costs, and limited energy-level tunability. Perovskite solar cells (PSCs), having perovskite materials as the light-harvesting-active layer, have recently emerged as a promising photovoltaic technology and are considered viable alternatives to silicon-based solar cells. Since their inception in 2009, PSCs have attracted considerable attention because of the rapid increase in their power conversion efficiencies (PCEs) from 3.81% to 25% and their ability to be produced at low cost with no limitations on processing size owing to their inherent nature.^9,10^ The materials used to make PSCs are known as perovskites, and they are characterized by a specific crystal structure with the formula ABX_3_, where A is a cation; B is a metal cation, such as lead(ii); and X is an organic ion, such as formamidinium (FA) or methylammonium (MA). These materials exhibit a remarkable enhancement in PCE (3.8–25.5%) because of their outstanding optoelectronic properties, which include extended charge-carrier diffusion lengths, high absorption coefficients, and tunable band gaps.^11^ Nevertheless, in spite of their promising developments, PSCs still face the challenges of material instability, toxicity and poor long-term operational stability of lead-based perovskites, which must be addressed in order to facilitate high performance; furthermore, synthesis and doping processes of PSCs are not efficient, and most of the doped additives utilized to date compromise device stability.^12^ Two of the most important components of PSCs are hole transport materials (HTMs), which serve as an interfacial hole-transporting layer that enables improved hole extraction and transport,^13^ and electron transport materials (ETMs), which pull electrons out of the perovskite absorber layer and carry them to the electrode and play a selective hole-barrier role that is necessary to ensure low recombination losses.^14^ This trade-off among stability, efficiency and cost has recently been the subject of research in the HTM community to eliminate them.^15^ Spiro-OMeTAD is the most commonly used HTM in PSCs thus far and has been proven valuable in facilitating high performance, but like all other materials, it has several drawbacks, including its high cost and complex synthetic processes and doping requirements; most dopant additives affect its stability.^16^ Consequently alternative organic and inorganic materials are being sought in research on HTMs. New activity in the HTM community has been dedicated to the eradication of this stability/efficiency/cost trade-off. The majority of new materials, such as dopant-free organic HTMs, hybrid organic-inorganic HTMs and 2D materials, have demonstrated performance comparable to or greater than that of Spiro-OMeTAD.^17^ Moreover, the interactions between the HTM layers and perovskite and optimization of the interface quality have also been reported as key factors influencing device performance. Organic HTMs, such as small molecules and polymers, are promising solutions that can be readily tailored to specific requirements, and in addition, they can be processed in solution; however, because of their sensitivity to environmental conditions, careful molecular design is usually a necessity. On the one hand, inorganic HTMs like CuSCN and NiO are very stable and exhibit good energy-level alignment; on the other hand, they can be affected by interfacial recombination and have limited flexibility with respect to processing conditions.^18^ Although there are a number of suitable materials that could be used to design HTMs, planar conjugated backbones with fused aromatic units are still one of the most appealing ones due to their uniform backbone conjugation distribution.^19,20^

Machine learning (ML) is an important branch of data science that enables computers to learn the patterns in data and make accurate predictions. It has widespread applications in data analysis and decision-making, including classification, regression, and clustering tasks. It can be a powerful tool for data analysis and decision making. The quality and quantity of the data used to train ML models are crucial to their performance. However, the trend of using ML to carry out complex data analysis and improve the quality of the prediction is growing. In recent years, ML has gained considerable attention in the fields of materials science and chemistry to accelerate the materials design and discovery process. However, the approach is counter-balanced by the traditional trial-and-error experimentation. Data-driven approaches and computational approaches, such as DFT, can identify promising candidate materials with desirable electronic and optoelectronic properties. These methods are used for the prediction of important parameters, like power conversion efficiency (PCE), *E*_gap_, and HOMO–LUMO levels, in photovoltaic materials like perovskites and devices such as organic solar cells. In addition, machine learning models are also employed to build quantitative structure–property relationships (QSPR) between molecular structures and properties. Thus, the use of ML-based approaches is considered an effective way to accelerate the discovery of materials and the development of next-generation energy technologies.^21–23^

Literature studies have demonstrated that binaphthyl acrylonitrile-based compounds exhibit high photovoltaic performance due to their unique structures and electronic properties. Binaphthyl acrylonitrile-based derivatives have demonstrated significant potential as efficient HTMs in both organic and perovskite solar cells, primarily due to their excellent film-forming capabilities and deep-seated HOMO levels. The intrinsic 3D-twisted configuration of the binaphthyl core effectively inhibits molecular over-aggregation and crystallization, leading to the formation of stable, amorphous thin films that ensure uniform contact with the photoactive layer. By incorporating acrylonitrile units, the molecular architecture gains increased oxidative stability and a dipole-moment-induced alignment that enhances the work function match with the anode. These materials exhibit high hole mobility and low interfacial resistance, which are critical for reducing recombination losses and improving the overall open-circuit voltage and long-term device stability.^24,25^ Literature studies have demonstrated that the utilization of heterocyclic donors in the molecular engineering of HTMs or OSCs improves the photovoltaic properties of organic chromophores, owing to their planar structures with improved π-conjugation.

**Scheme 1:**
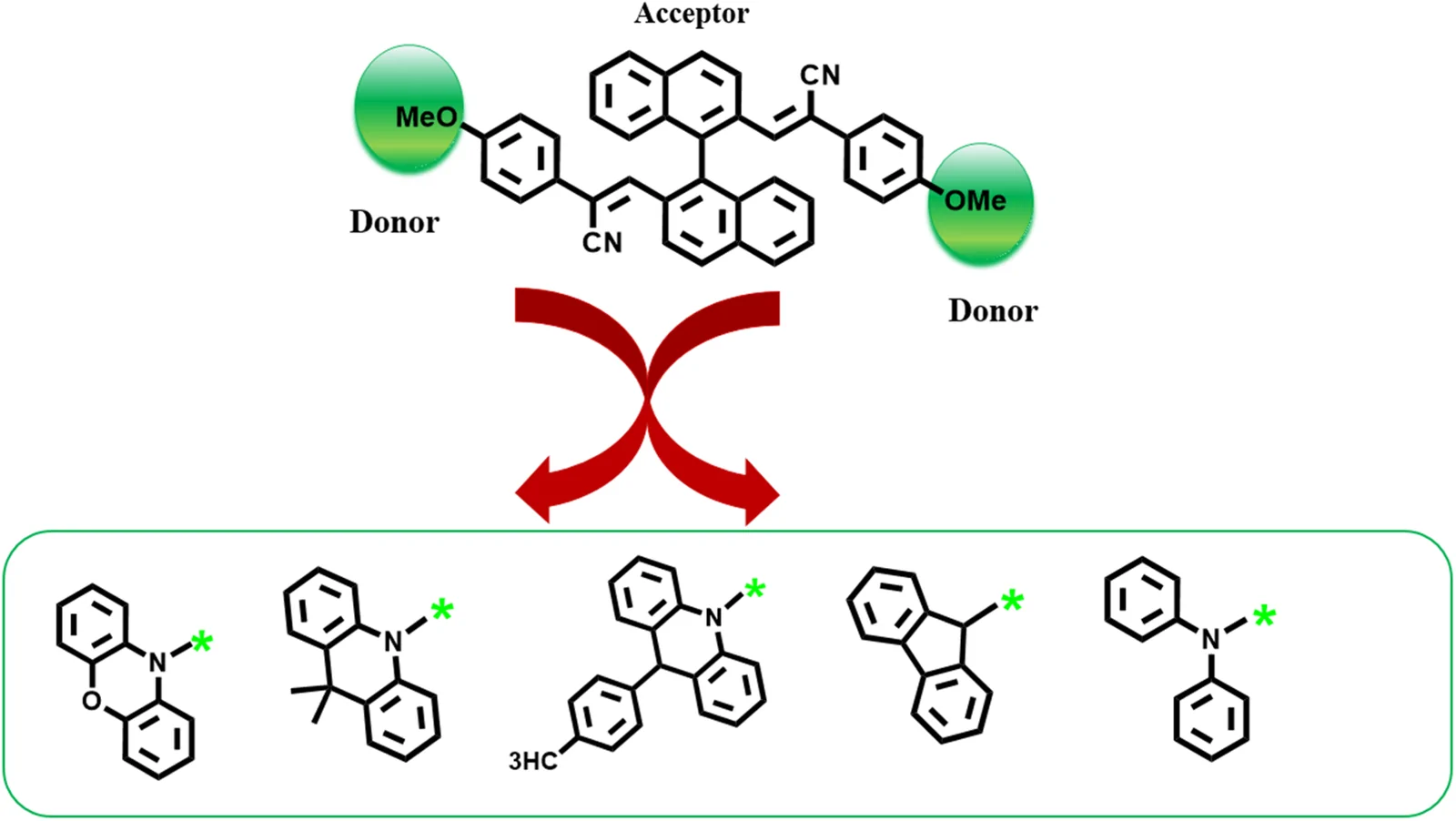
Systematic representation of the molecular engineering of BNPR with a heterocyclic donor to design BNPD1–BNPD5.

The present study focuses on designing HTMs through the molecular engineering of the binaphthyl acrylonitrile core with heterocyclic donors to improve their optical properties. For this purpose, a synthesized organic chromophore, Bi-MeOCN, was taken as the reference chromophore and renamed as BNPR. Various types of heterocyclic donors were utilized at both ends to design new derivatives (BNPD1–BNPD5) for use as HTMs in PSCs (Scheme 1). A literature review revealed that the use of heterocyclic donors with the binaphthyl acrylonitrile core as HTMs has not been studied previously. The *E*_gap_ of the designed binaphthyl acrylonitrile-based chromophores was predicted using the best model. In order to enhance model interpretability, shapley additive ex planations (SHAP) analysis was performed to identify the molecular properties that exert the greatest influence on the predicted *E*_gap_ values. Further, statistical verification, including the Kolmogorov–Smirnov (KS) test for normality, and a synthetic accessibility test were conducted to validate the reliability and practical feasibility of the predicted chromophores, respectively. Typically, the ML-predicted *E*_gap_ values were validated against DFT calculations. This comparison demonstrates the effectiveness and reliability of the proposed model in predicting the electronic properties of the chromophores. The optical and photovoltaic characteristics of these chromophores were explored through quantum chemical calculations (DFT/TD-DFT). It is anticipated that these materials might exhibit good optoelectronic properties and can be reasonable candidates for utilization as organic HTMs.

### Machine learning methodology

#### Dataset collection and preprocessing

In total, 1693 chromophores were collected from published computational studies reporting *E*_gap_ values calculated using density functional theory (DFT). The molecular structures were drawn and described using simplified molecular input line entry system (SMILES)^26^ notation, along with the corresponding *E*_gap_ values. A systematic workflow for data preprocessing was used to ensure data quality and consistency. First, duplicate molecular structures were identified using their SMILES string and discarded. The *E*_gap_ values were compared for duplicate SMILES to determine the consistency of labels. In this way, duplicate structures were kept if they showed the same *E*_gap_ value, while others with conflicting *E*_gap_ values were deleted to avoid label ambiguity during model training. This process led to the elimination of 60 records, namely, 36 with small *E*_gap_ differences (≤0.10 eV) and 24 with large differences (>0.10 eV), leaving a total of 1632 chromophores. Next, those SMILES strings that were identified as invalid were checked using the RDKit cheminformatics toolkit.^27^ There were 1552 valid molecular entries, while 80 chemically invalid structures were identified and excluded. All canonical SMILES were generated from all valid structures using RDKit to remove redundant molecular representations. 155 more chromophores were removed as duplicate molecules with the same canonical SMILES. Thus, the final selected data set consisted of 1396 unique and chemically correct chromophores.

### Molecular feature generation

The molecular descriptors and fingerprints were generated using the cheminformatics package RDKit within the Anaconda Navigator (https://www.anaconda.com/docs/tools/anaconda-navigator/main) and Jupyter Notebook^28^ environments. A total of 217 physicochemical and topological molecular descriptors were calculated, and 2048-bit Morgan circular fingerprints were calculated for each chromophore. No additional data were removed due to descriptor calculation failures, as indicated by missing values (NaN).^29,30^ This resulted in a set of 2267 columns: the target property (*E*_gap_), the molecular representation (SMILES), 217 RDKit descriptors, and 2048 Morgan fingerprint features.^31^ After generating the features, the first feature matrix had 2265 predictor variables. In addition, the descriptor and Morgan fingerprint datasets were stored in Excel and CSV formats for further analysis.

### Feature preprocessing and selection

Some feature preprocessing steps were taken to decrease dimensionality and eliminate redundant information before model development. Features were then removed if they had more than 20% missing values, and constant and duplicate features were removed. The feature space was reduced from 2265 to 2017 variables by these preprocessing steps. The SelectKBest (https://scikit-learn.org/stable/modules/generated/sklearn.feature_selection.SelectKBest.html) algorithm, based on the F-statistic (f_regression) (https://scikit-learn.org/stable/modules/generated/sklearn.feature_selection.f_regression.html), was incorporated into the ML workflow as an additional feature selection step. During hyperparameter optimization, the number of selected descriptors (100, 250, 500, 750, 1000, 1500, 2000, and all available descriptors) was evaluated. This approach helped identify the most informative molecular properties while avoiding irrelevant and redundant descriptors.

### Model development and validation

**Fig. 1:**
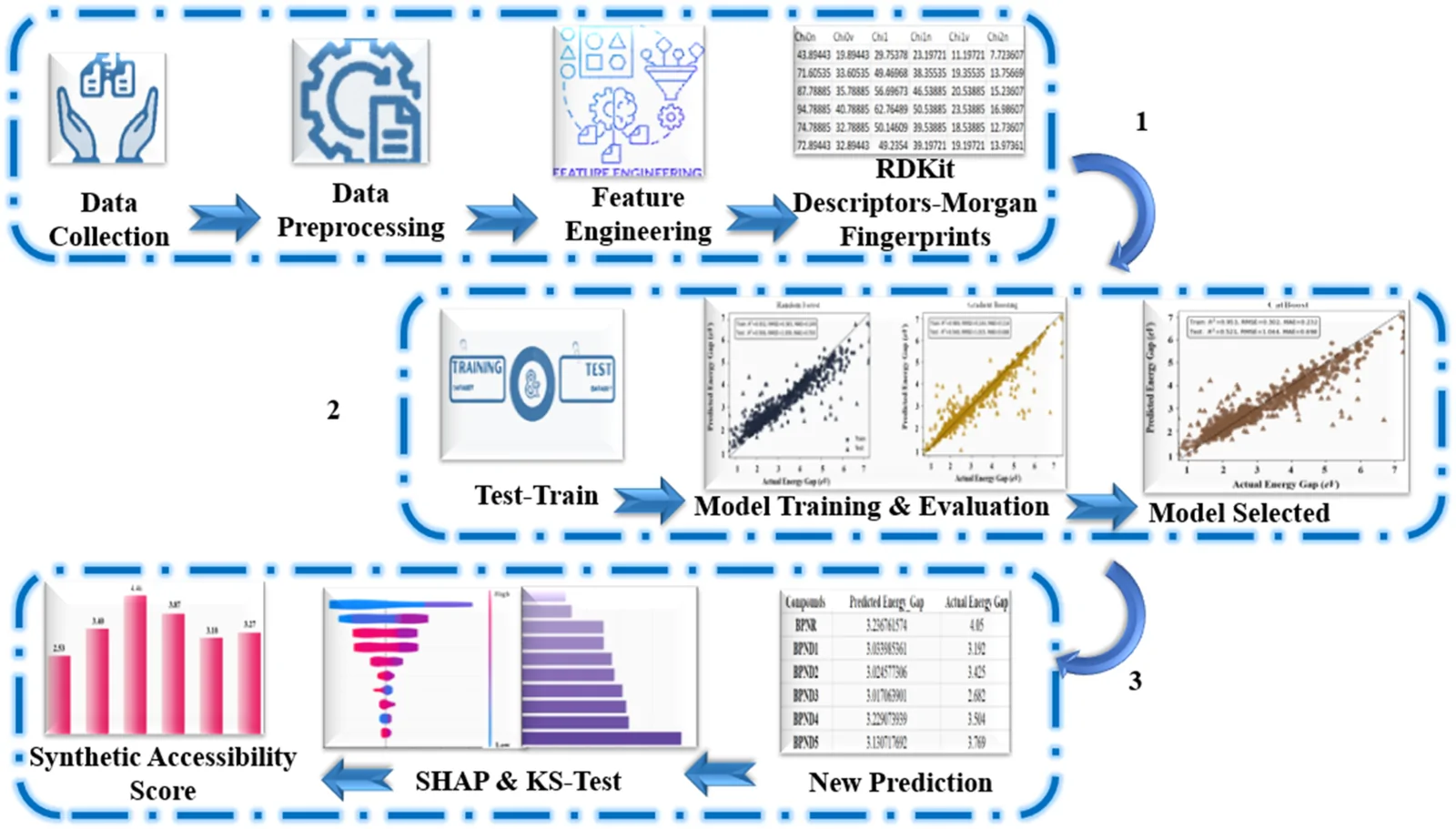
Complete machine learning workflow adopted in the current study.

After preprocessing, a final data set of 1396 valid chromophores was generated and split into separate training, validation, and test sets. The training and validation sets were only used for development of model, hyperparameter optimization, and feature selection procedures, while the test set was used only for the final stage of model evaluation. This protocol allowed for an unbiased assessment of the generalization performance of the model and prevented overfitting. The model robustness and stability were further investigated by adopting a 5-fold cross-validation during model training. In this procedure, the training data were divided into 5 equal parts, with the first 4 parts used for training and the remaining part used for validation. This process was performed 5 times, with each fold serving as the validation set once, and the mean accuracy after each fold was used for model selection and optimization.^32^ The curated descriptor/fingerprint database was used to test several ML algorithms. The performance of the model was evaluated by calculating the coefficient of determination (*R*^2^), mean absolute error (MAE) and root mean square error (RMSE) according to eqn (S1)–(S3).^33^ CatBoost showed the highest predictive capabilities among the ensemble algorithms considered, and was chosen as the final algorithm to predict *E*_gap_. The process of rigorous data curation, feature engineering, feature selection, independent validation, and cross-validation ensured the integrity, methodological reproducibility, and reliability of the predicted *E*_gap_ values for the organic chromophores. Fig. 1 illustrates the complete machine learning workflow.

### Computational method

The Gaussian 16 program^34^ was used to perform all calculations in the current study. The DFT/TD-DFT methodologies were employed using the M06 functional^35^ with the 6-311 G(d,p) basis set^36^ to investigate the optoelectronic properties of BNPR and BNPD1–BNPD5. Initially, the geometrical optimization of the designed chromophores was performed, and the absence of imaginary frequencies confirmed the successful optimization of the structures. After the optimization of the chromophores, different analyses were performed, like FMOs, GRP, UV-Vis, DOS and TDM, to study their charge-transfer phenomena and optoelectronic characteristics. In order to check the effect of chloroform solvent, the CPCM model was utilized. To generate the input files and to view the structures, Gauss View 5.0 ^37^ was utilized. Various software packages, such as Chem Craft,^38^ Avogadro,^39^ GaussSum,^40^ PyMOlyze,^41^ and Multiwfn,^42^ were used to analyze the data from Gaussian log files. Origin 8.0 (ref. 43) was used to generate UV-Vis spectra from the data obtained through GaussSum from Gaussian log files.

## Results and discussion

**Fig. 2:**
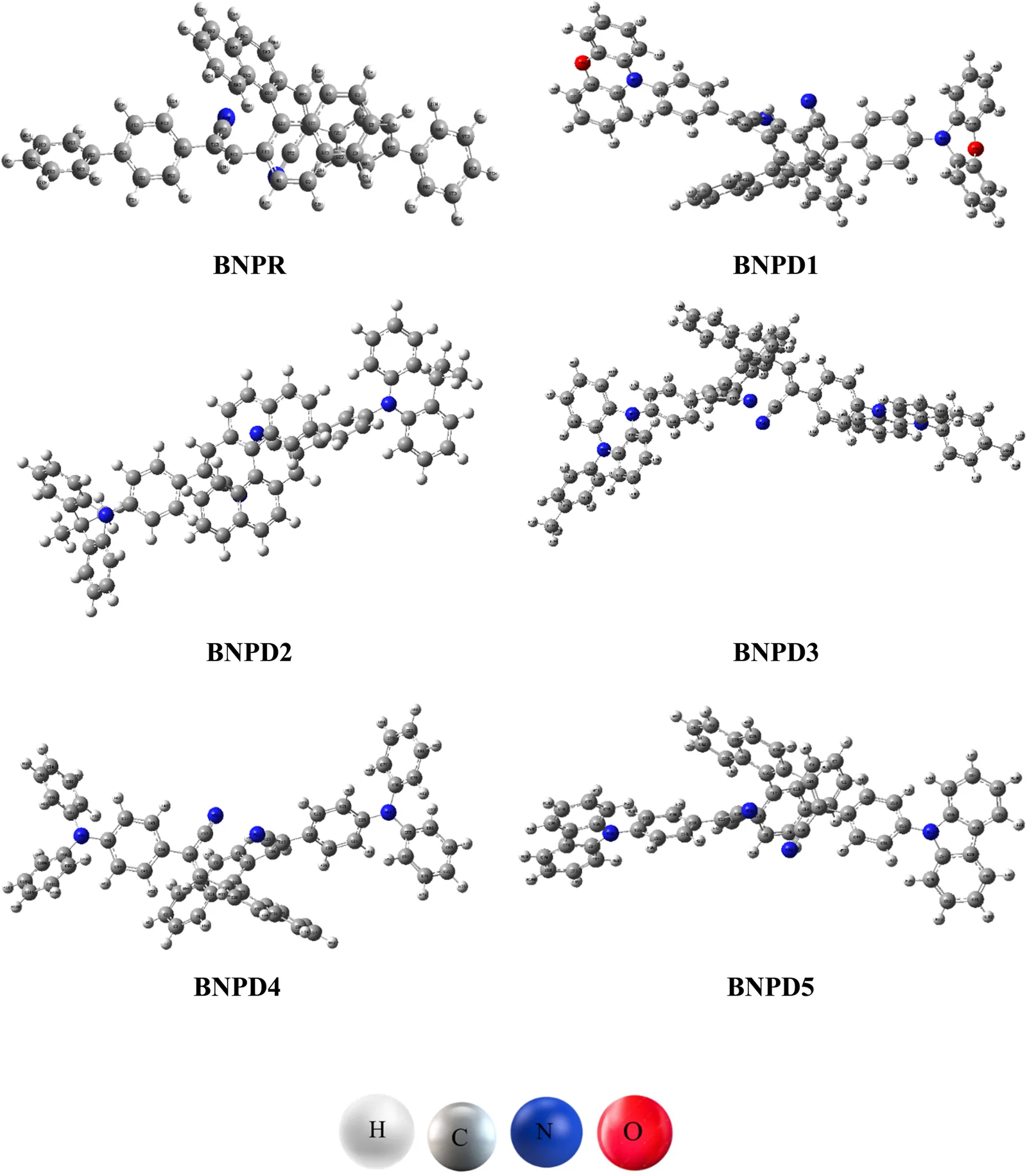
Optimized geometries of the BNPR and BNPD1–BNPD5 chromophores.

A synthesized organic chromophore, Bi-MeOCN,^24^ was selected as the reference chromophore to design five new chromophores (BNPD1–BNPD5) as HTMs. In the current study, the synthesized Bi-MeOCN chromophore was take renamed as BNPR, and consists of a binaphthyl acrylonitrile core as the acceptor and a methyl benzene moiety. Five different chromophores (BNPD1–BNPD5) were designed by attaching different heterocyclic donor units to both terminals of the core. Both ends comprise the same donor; the purpose of designing these five new chromophores was to explore the effects of different donors on the optoelectronic and photophysical properties of the binaphthyl acrylonitrile core. The donors utilized for designing the chromophores are as follows: 10H-phenoxazine in BNPD1, 9,9-dimethyl-9,10-dihydroacridine in BNPD2, 9-(*p*-tolyl)-9,10-dihydroacridine in BNPD3, triphenylamine in BNPD4, and 9H-fluorene in BNPD5. The ChemDraw structures of the reference chromophore (BNPR) and its designed chromophores (BNPD1–BNPD5) are shown in Scheme 1 and Fig. S1, along with their IUPAC names. The Cartesian coordinates of all the optimized structures are provided in Tables S1–S6. A pictorial representation of the optimized geometrical structures of the reference and designed chromophores is presented in Fig. 2.

### Model training and selection

**Fig. 3:**
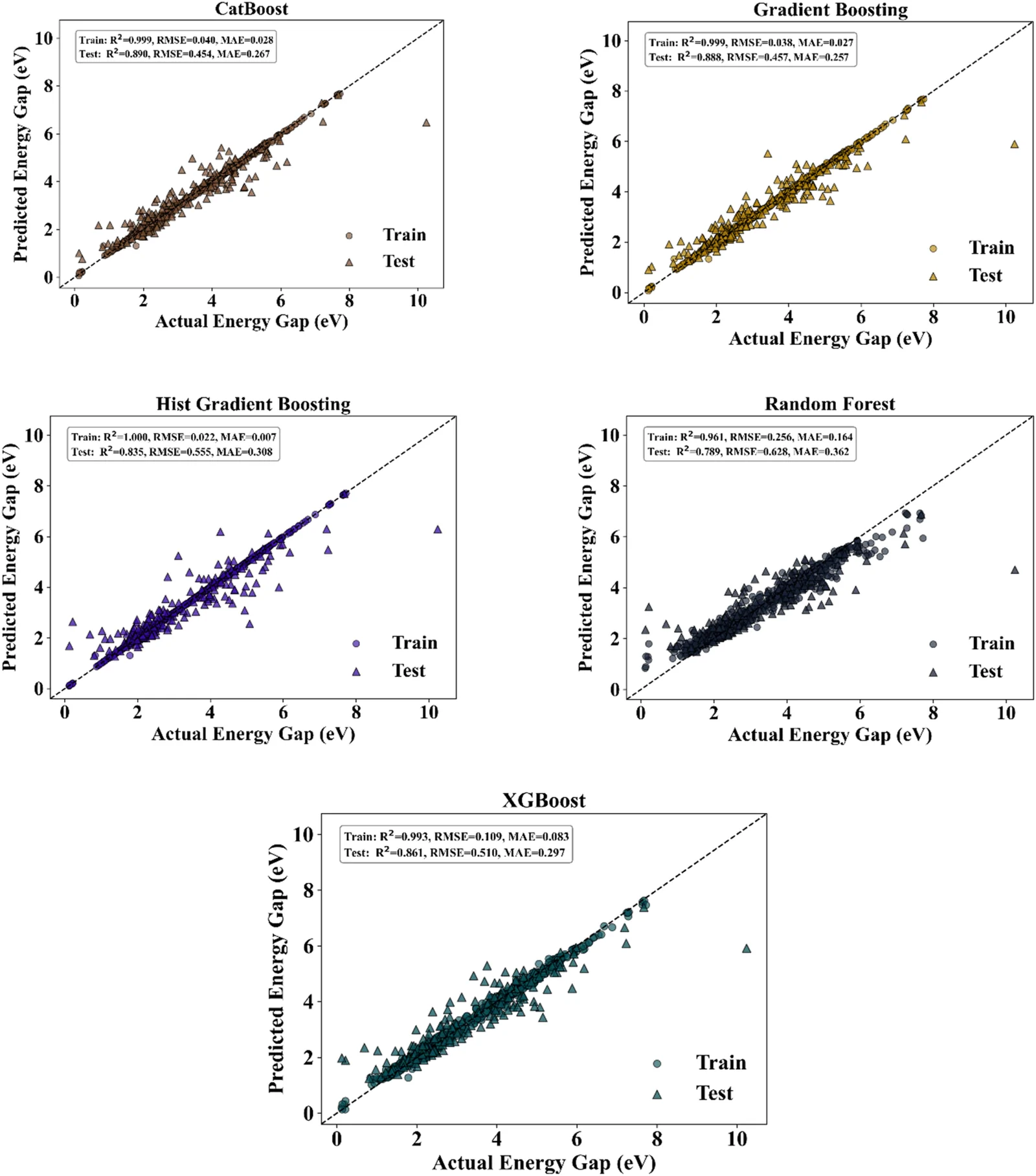
Calibration plots between the actual and predicted energy gaps of ML models (XGBoost, random forest, hist gradient boosting, CatBoost, and gradient boosting).

The calibration plots (Fig. 3) illustrate the relationship between the actual and predicted *E*_gap_ values of the training and test datasets. Furthermore, the models employed were XGBoost, random forest, hist gradient boosting, CatBoost, and gradient boosting.^44^ The dashed diagonal line is characterized as an ideal case where the predicted *E*_gap_ values are perfectly matched to the actual/calculated *E*_gap_ values. Among the training datasets, the employed models display strong performance. As mentioned above in the methodology section, model performance was evaluated using various evaluation metrics. Table S7 comprises the train and test *R*^2^ values along with their error parameters (RMSE and MAE). Random forest, gradient boosting, histogram gradient boosting, XGBoost, and CatBoost models were evaluated on an independent test set and using 5-fold cross-validation. CatBoost had the highest predictive accuracy among the models, with a test *R*^2^ value of 0.890, test RMSE of 0.454 eV, and test MAE of 0.267 eV; that is, it was able to explain almost 89% of the variance in unseen chromophores.

Gradient boosting had similar predictive ability (test *R*^2^ = 0.888), while XGBoost, Histogram Gradient boosting, and random forest had relatively poor predictive ability. The robustness of the models was also evaluated using 5-fold cross-validation. A statistical method called cross-validation (CV) is used to understand how well a machine learning model will perform on new, unseen data. Rather than performing train–test splitting once, the data are split several times into a training set and a validation set, with the model being tested on a different split of the data each time. Consistent with the 5-fold cross-validation results (see Table S8), CatBoost achieved the highest average CV *R*^2^ (0.8674 ± 0.0220), together with the lowest CV RMSE (0.4727 ± 0.0500 eV) and CV MAE (0.2813 ± 0.0156 eV). Small standard deviations among the different validation folds show consistent and reliable performance, indicating that the structure–property relationships learned are not highly sensitive to the specific way the data is split into validation folds. In conclusion, the model accuracy on the accuracy test set and the accuracy obtained from the cross-validation results show that the CatBoost model has good generalization performance. CatBoost demonstrated the highest predictive performance, had the lowest prediction errors, and was consistent across all validation folds; therefore, it was chosen as the best model for further analysis and prediction of *E*_gap_ of unseen chromophores within the chemical space covered by the current dataset.

### Energy gap prediction of the binaphthyl acrylonitrile-based chromophores

Based on the above ML analysis, CatBoost was the most promising predictive model identified in this study. It was an efficient model for predicting the *E*_gap_ of binaphthyl acrylonitrile-based chromophores based on the molecular descriptors calculated using RDKit. CatBoost is an ensemble gradient boosting algorithm based on sequential decision trees. Each tree attempts to reduce the errors of the previous trees and enhance prediction performance. It has been shown to reduce overfitting and enhance generalization, especially when dealing with high-dimensional and complex data.^45,46^ To determine the structure–property relationships, a quantitative structure–property relationship (QSPR) model was adapted. Moreover, its feature importance analysis became the key in the selection of descriptors for better model interpretability. The default and optimal hyperparameters of the CatBoost model are tabulated in Table S9. The step-by-step algorithm of CatBoost is also provided in the SI File. The model was demonstrated to be reliable and stable, proving that the predicted *E*_gap_ values are highly correlated with the calculated *E*_gap_ values obtained by DFT and TD-DFT. Moreover, the predicted and actual *E*_gap_ values of the binaphthyl acrylonitrile-based chromophores are tabulated in Table S10. Overall, the results indicate that predictive accuracy lies in the range of 79.90–95.05%. This suggests that it is a valuable tool for the screening of new chromophores.

### SHAP analysis

**Fig. 4:**
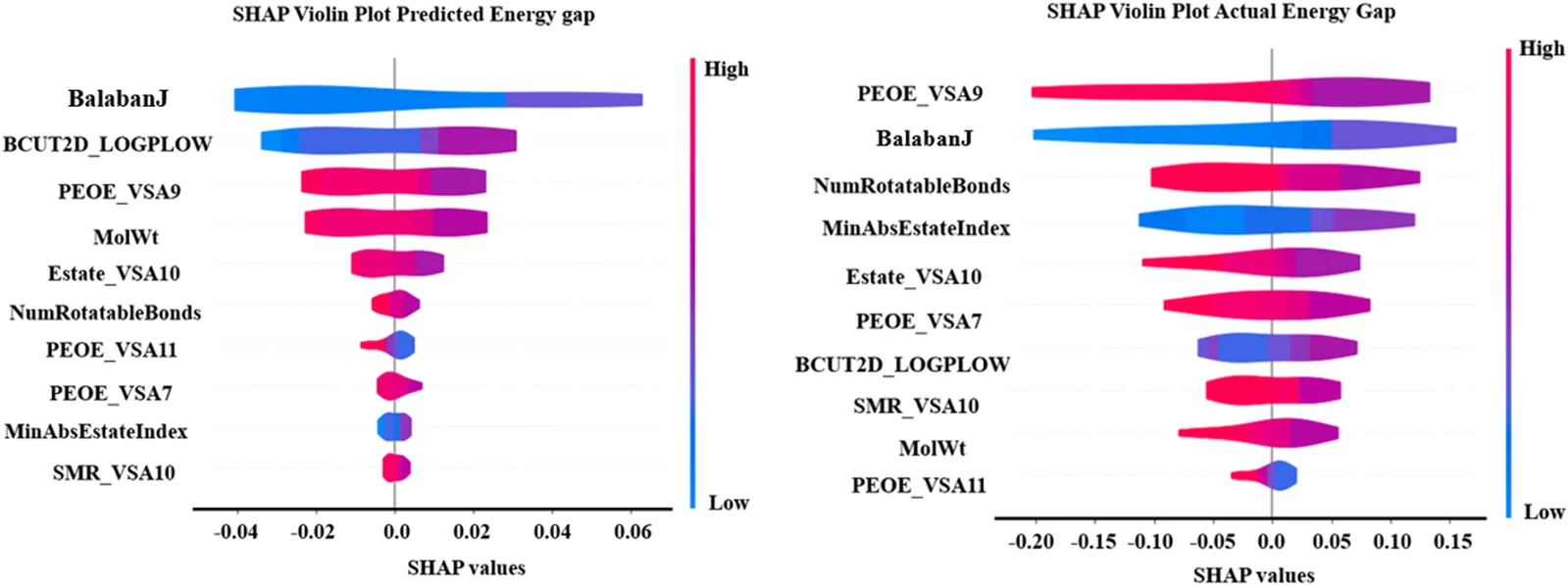
SHAP violin plots of top 10 descriptors *vs.* actual and predicted energy gaps.

The SHAP violin plots (see Fig. 4) illustrate the impact of molecular descriptors on the predicted and actual *E*_gap_ values. SHAP analyses were first introduced in 2010 by Štrumbelj and Kononenko and are based on Shapley values. A major advantage of SHAP values is that they measure the effect of features on each sample, both positively and negatively.^47,48^ The most significant descriptors are those at the top, with a broader distribution and longer tail of SHAP values. Descriptions of selected descriptors are tabulated in Table S11. The color bar (blue = low and pink/red = high) shows the effect of feature values on the *E*_gap_ value. Positive SHAP values suggest a positive correlation (higher *E*_gap_) and negative values suggest a negative correlation (lower *E*_gap_). A combination of blue and pink/red indicates non-linear and context-sensitive effects. For the predicted *E*_gap_ values, the most important descriptors are BalabanJ, BCUT2D_LOGPLOW and PEOE_VSA9. In case of the BalabanJ descriptor, there is a clear pattern, with higher values (pink/red) generally located towards the positive SHAP scale. This suggests that higher molecular connectivity and topological complexity generally correspond to a higher predicted gap. BalabanJ is a chemical topological index that represents the connectivity and branching pattern of a molecule from a chemical point of view.^49^ The molecular topology of HTMs plays a significant role in determining the pathways of π-electron delocalization and spatial distribution of frontier molecular orbitals. Thus, the HOMO–LUMO separation can be changed by varying molecular connectivity, making BalabanJ a physically meaningful descriptor for *E*_gap_ prediction. In terms of HTM design, molecular connectivity and branching play a significant role in determining the level of π-conjugation, molecular packing, and charge-transport pathways. Thus, BalabanJ provides information on structural characteristics that govern electronic energy levels as well as the efficiency of hole transport in organic semiconducting material. Similarly, BCUT2D_LOGPLOW shows a preference for lower values (blue) towards negative SHAP values, while higher values towards positive SHAP value suggest that atomic partial charge and lipophilicity contribute to *E*_gap_. BCUT2D_LOGPLOW includes information about atomic properties and charge-weighted molecular environment. These characteristics affect electronic polarization, intermolecular interactions, and the stability of frontier orbitals, which all play a role in varying the electronic energy gap of HTMs.^50^ Similarly, the descriptor PEOE_VSA9 also shows a predominantly positive contribution at higher values, supporting the role of electronic surface area in increasing the predicted *E*_gap_ values. PEOE_VSA9 represents the van der Waals surface area of atoms within a specified range of partial charges. In conjugated HTM systems, charge distribution over the molecular surface influences the localization of electron density, orbital energies, and intermolecular charge-transfer interactions. Therefore, the high level of contribution from PEOE_VSA9 suggests that electrostatic properties are important factors in determining *E*_gap_. This descriptor is important for HTM design since it is a key factor in determining intermolecular electronic coupling, charge mobility, and frontier orbital energy alignment. Thus, PEOE_VSA9 describes electronic properties that are closely linked to effective hole transport and energy-level tuning of HTM molecules.^51^ In contrast, descriptors such as MolWt and EState_VSA10 exhibit a bidirectional distribution, which indicates that they have a non-monotonic influence on the prediction of *E*_gap_.

For instance, the molecular weight (MolWt) can be used as an indirect measure of molecular size and conjugation length, while EState_VSA10 is a combination of electronic-state and surface-area information.^51^ These two SHAP distributions are bidirectional, indicating that they affect *E*_gap_ in a variety of ways, depending on the molecular structure. Finally, the weak descriptors, like MinAbsEStateIndex and SMR_VSA10, have tighter distributions that are centered at zero, suggesting weaker effects and a less prominent role in the prediction of *E*_gap_. The actual *E*_gap_ plot shows a different ranking of descriptors, with PEOE_VSA9, BalabanJ, and NumRotatableBonds being the most important descriptors. PEOE_VSA9 is again prominently positive, with higher values having positive SHAP values, confirming that electronic surface area has a significant impact on the actual *E*_gap_. This observation is chemically sensible since the electronic surface properties of HTMs influence the energy levels of frontier molecular orbitals and charge localization. These are the main factors determining the energy difference between the HOMO and LUMO. In contrast, NumRotatableBonds shows a trend where higher values are more often associated with negative SHAP values, suggesting that greater molecular flexibility leads to lower *E*_gap_, possibly through increased conformational flexibility and decreased electronic delocalization. Implementing more flexible chromophores is a potential strategy for reducing *E*_gap_. Therefore, in HTMs, a higher number of rotatable bonds can lead to molecular planarity and less effective π-conjugation.^52,53^ These structural distortions could lead to changes in the overlap of different components involved and may promote changes in the energies of frontier orbitals. This, in turn, could impact the *E*_gap_ between the HOMO and LUMO, as well as the charge transport properties of chromophores. The MinAbsEStateIndex descriptor also exhibits a negative trend at higher values, suggesting that specific features of electronic states may lower the *E*_gap_. Based on its influence, it is proposed that the local electronic environment and charge distribution play a role in tuning the energy levels that contribute to *E*_gap_ through the electronic-state environment of atoms within a molecule, as described by MinAbsEStateIndex.^54^ Bi-directional (mixed) descriptors, such as EState_VSA10 and PEOE_VSA7, highlight the complex role of electronic and surface features.

Other descriptors, such as MolWt and PEOE_VSA11, have smaller magnitude contributions, implying minor adjustments to the actual *E*_gap_ values. Altogether, the SHAP analysis results reveal that conformational flexibility, surface electronic properties, charge distribution, and molecular topology all play a dominant role in influencing the behavior of *E*_gap_. Structural connectivity effects are captured by descriptors like BalabanJ, electronic distribution characteristics are captured by descriptors like PEOE_VSA9, which are charge-dependent, and molecular rigidity and effective conjugation are captured by descriptors like NumRotatableBonds. The HOMO–LUMO *E*_gap_ between these two states is directly related to the *E*_gap_ of HTMs. Furthermore, these factors, such as molecular conjugation, intermolecular interactions, electronic charge distribution, and molecular rigidity, are significant design parameters for the performance of HTMs because they influence charge transport and device efficiency.

### Kolmogorov–Smirnov (KS) normality test

**Fig. 5:**
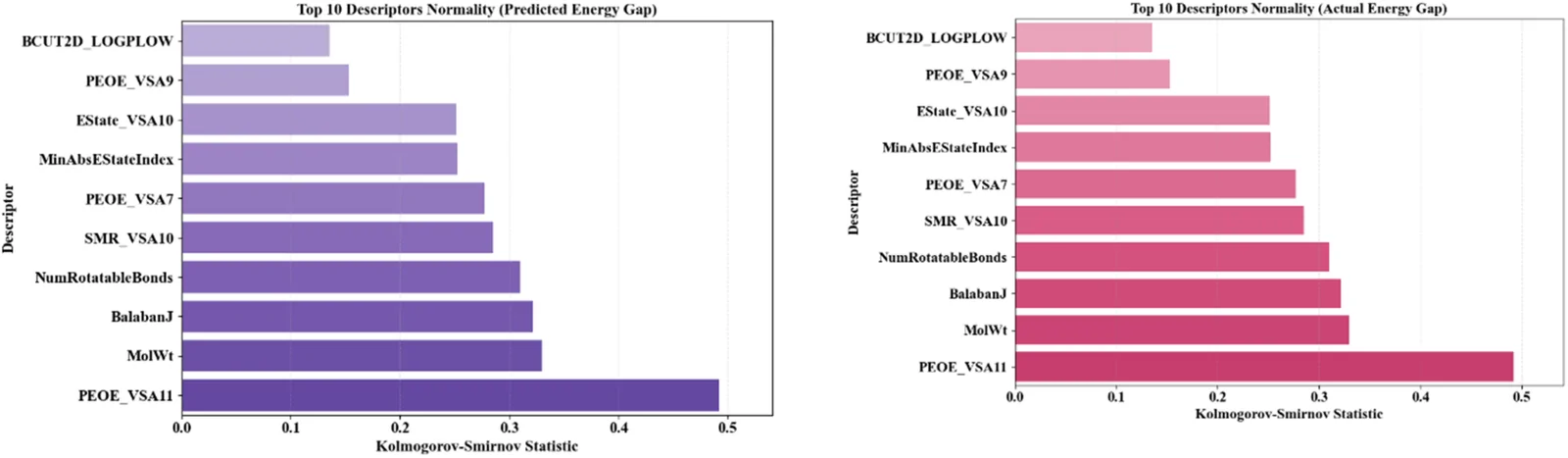
Bar plot illustration of the top ten descriptors *vs.* the actual and predicted energy gaps.

Normality plots (see Fig. 5) of the Kolmogorov–Smirnov (KS) statistic provide indications of the distribution of the top ten molecular descriptors influencing the predicted and actual *E*_gaps_. The Kolmogorov–Smirnov test is a non-parametric goodness-of-fit test that measures the significance of the largest difference between the predicted and actual cumulative distributions.^55^ In these plots, the descriptors are ordered by the KS statistic values, where smaller values correspond to distributions closer to the normal distribution, whereas larger values correspond to distributions farther from the normal distribution. These plots are vital for understanding the statistical nature of descriptors and how they can be used in machine learning approaches. For the predicted *E*_gap_ plot, descriptors BCUT2D_LOGPLOW and PEOE_VSA9 have the lowest KS statistic values. This suggests that their distributions are closer to a normal distribution and, therefore, are more statistically normal. However, descriptors like PEOE_VSA11, MolWt, and BalabanJ have higher KS values, indicating significant departure from normality. It is interesting to note that electronic surface area descriptors (PEOE_VSA descriptors) are generally associated with medium-to-high KS values due to their variable distributions and variations among chromophores.

Similarly, descriptors related to molecular structure, like NumRotatableBonds and BalabanJ, also show moderate deviation, highlighting the variability in the molecular structure and flexibility within the dataset. In the actual *E*_gap_ plot, a similar but slightly different trend can be seen. Again, BCUT2D_LOGPLOW and PEOE_VSA9 show lower KS values, indicating a relatively normal distribution and consistent pattern in the predicted and actual plots, respectively. However, the descriptor PEOE_VSA11 appears with the highest KS statistic, implying a significant departure from normality and suggesting a highly non-normal (or possibly multimodal) distribution in the actual *E*_gap_ plot. Descriptors like NumRotatableBonds, BalabanJ, and MolWt demonstrate moderate KS statistics, suggesting some degree of deviation from normality but also some level of distributional consistency. Descriptors such as EState_VSA10 and MinAbsEStateIndex have medium KS values, implying that they are moderately spread and balanced descriptors. Therefore, these distribution profiles demonstrate that although a few descriptors are close to the normal distribution, most of them are non-normal with different levels of deviation, reflecting the inherent complexity of the molecular descriptor space.

### Synthetic accessibility score

**Fig. 6:**
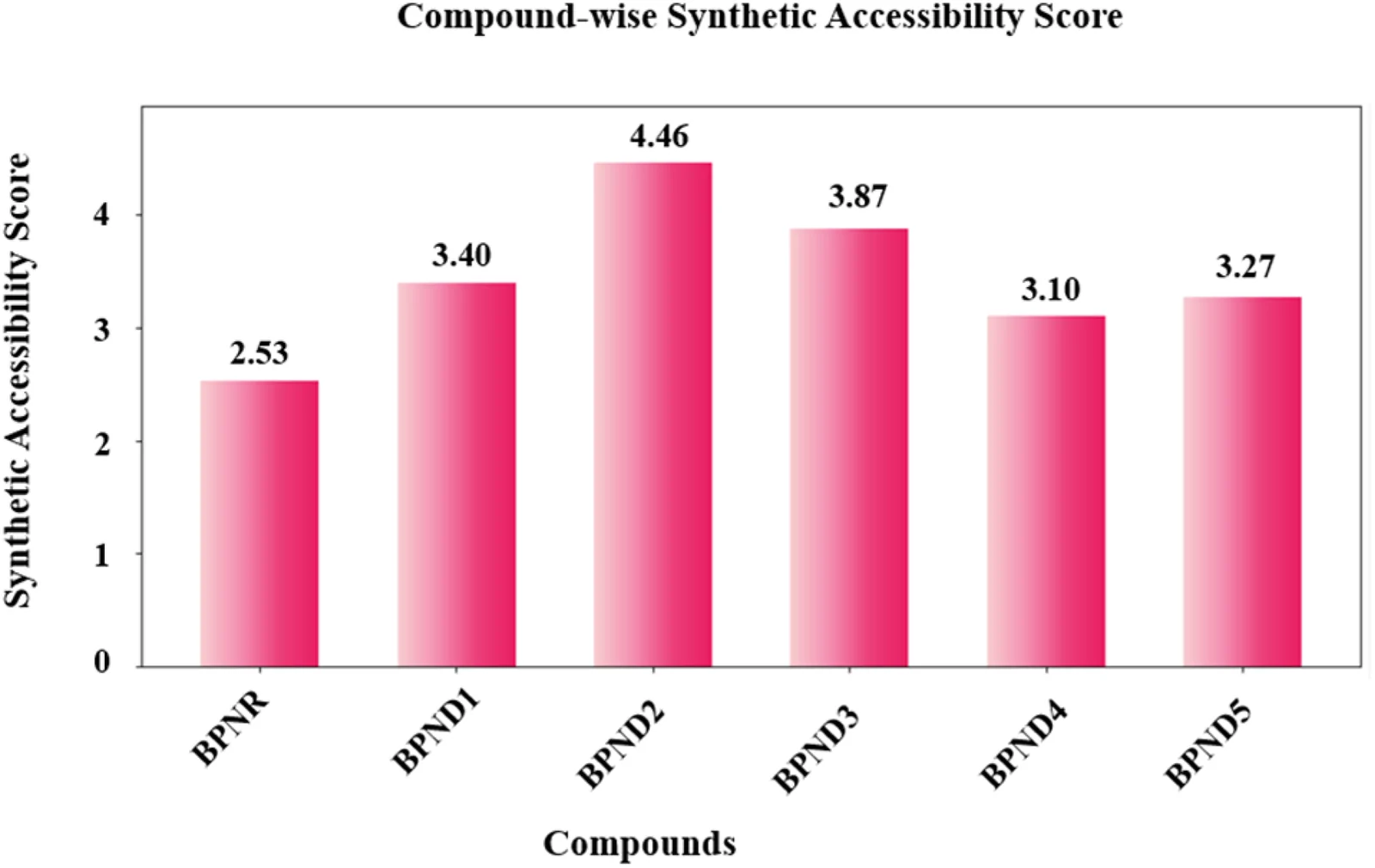
Bar plot illustration of the synthetic accessibility score of the investigated chromophores.

Synthetic accessibility scores (SAS) give an indication about the ease or difficulty with which chromophores can be synthesized in the laboratory.^56^ A lower SAS indicates that the compound is easier to synthesize, whereas a higher SAS indicates that the compound is more difficult to synthesize in the laboratory. Fig. 6 demonstrates a significant range of synthetic accessibility scores among the investigated chromophores. This reflects significant variation in their synthetic routes due to differences in their structural complexity. Among the investigated chromophores, BPNR exhibits the lowest SAS. This proves that it is more accessible and easier to synthesize in the laboratory through conventional synthetic routes. Similarly, BNPD4 and BNPD5 exhibit low SAS of 3.10 and 3.27, respectively, suggesting their moderate synthetic accessibility. Among the series, BNPD1 (3.40) displays a slightly higher SAS but remains within a controllable range for synthesis. On the other hand, BNPD3 (3.87) and especially BNPD2 (4.46) exhibit much higher SA scores, suggesting that their structures are more complex and may contain multiple functional groups or steric effects, which could make them more difficult to synthesize. The decreasing trend in SAS is as follows: BNPD2 > BNPD3 > BNPD1 > BNPD5 > BNPD4 > BNPR. Among the investigated systems, BNPD2 may require a complex synthetic approach due to its higher SAS.

SAS does not directly provide reaction pathways; however, it is useful for estimating synthetic feasibility based on molecular complexity and fragment contributions.^57^ The proposed chromophores have a D–A–D structure with a binaphthyl–acrylonitrile framework. The donor substituents can be suitably modified, followed by functionalization of the binaphthyl backbone and, finally, connection of the terminal acrylonitrile through either Suzuki–Miyaura, Heck or Buchwald–Hartwig cross-coupling reactions.^58^ Possible experimental challenges include steric hindrance from bulky donor groups, low coupling reaction yields, purification of highly conjugated intermediate products, and improving the overall reaction yield. However, the relatively low SAS of BNPR, BNPD4, and BNPD5 suggests their experimental feasibility through conventional organic synthetic methods. Overall, the proposed approach of using SAS analysis along with the machine-learning-inspired screening is a practical way for identifying chromophores that are both computationally promising and potentially implementable in the laboratory. SAS analysis combined with model prediction provides new routes towards the realistic selection of chromophores for practical applications.

## Prediction validation by DFT and TD-DFT

### Frontier molecular orbital (FMO) analysis

The chemical stability, reactivity and optoelectronic properties of compounds are strongly governed by the FMO energies.^59–67^ The FMO energies provide quantitative and visual insights for the characterization of the optoelectronic and photovoltaic properties of compounds in solar cells.^64^ Chromophores with high energy gaps resist changes in electronic configuration and exhibit low polarizability, high kinetic stability, and low chemical reactivity, and are generally considered hard chromophores, and *vice versa*. For the development of solar cell materials, soft compounds are highly efficient and exhibit strong intermolecular charge transfer.^68–71^ The bonding and anti-bonding properties of chromophores in their ground and excited states can be described by their HOMOs and LUMOs, respectively. The HOMO acts as a donor, while the LUMO acts as an acceptor, collectively dictating the chemical behavior and ICT of the molecule.^72^ The molecular orbital energies of the designed compounds were investigated, and the HOMO/LUMO results are presented in Table 1. The other molecular orbital energies (HOMO−1/LUMO+1 and HOMO−2/LUMO+2) are shown in Table S12. Pictographs illustrating the ICT in MOs are shown in Fig. 8 and S2.

**Table 1:** FMO energies of designed chromophores (eV)

Compound	*E*_HOMO_	*E*_LUMO_	Δ*E*^a^
BNPR	−6.264	−2.214	4.050
BNPD1	−5.428	−2.236	3.192
BNPD2	−5.638	−2.213	3.425
BNPD3	−4.896	−2.214	2.682
BNPD4	−5.671	−2.167	3.504
BNPD5	−6.076	−2.307	3.769

a Energy gap = *E*_LUMO_ – *E*_HOMO_ (eV).

The energy gap of the reference chromophore was found to be greater than those of its derivatives when modified with various heterocyclic donor moieties. Among the derivatives, BNPD5 exhibited the highest energy difference between the HOMO and LUMO (3.769 eV) due to its donor moiety (9*H*-fluorene), which reduces resonance effects, resulting in a higher energy gap compared with the other chromophores.^73,74^ A slight change in the *E*_gap_ value (3.504 eV) occurs when the 9*H*-fluorene donor moiety is replaced with diphenylamine in BNPD4. This energy difference is further narrowed when the 9,9-dimethyl-9,10-dihydroacridine donor is introduced in BNPD2, owing to its strong donating ability and improved resonance. Further reduction in the energy gap was observed by introducing the 10*H*-phenoxazine donor moiety in BNPD1. The smallest energy gap was observed in BNPD3 (2.682 eV) due to the strong electron donating ability of the 9-(*p*-tolyl)-9,10-dihydroacridine donor moiety. The decreasing order of energy gaps among the designed chromophores is BNPD5 > BNPD4 > BNPD2 > BNPD1 > BNPD3, with values of 3.769 > 3.504 > 3.425 > 3.192 > 2.682 eV. Among all the designed molecules, BNPD4 (9-(*p*-tolyl)-9,10-dihydroacridine) exhibits the lowest energy gap. Although BNPD2 and BNPD3 have similar structures, the substitution of a benzene ring in BNPD3 enhances its conjugation and electron-donating capability, resulting in a lower energy gap.^75–77^

Spiro-OMeTAD is widely employed as a benchmark HTM in PSCs, with a reported HOMO energy level of approximately −5.2 eV, LUMO of around −2.2 eV, and an energy gap of ∼3.0 eV. These electronic features ensure efficient hole extraction from the perovskite absorber while minimizing electron back-transfer.^78^ In comparison, the BNPR and BNPD1–BNPD5 chromophores exhibit HOMO energy levels ranging from −6.264 to −4.896 eV, indicating tunable hole-donating characteristics through molecular engineering. Among them, BNPD3 (−4.896 eV) presents a shallower HOMO level than Spiro-OMeTAD, which may facilitate more efficient hole injection and reduce the energetic barrier at the perovskite/HTM interface. Conversely, compounds such as BNPR and BNPD5, with deeper HOMO levels (≤−6.0 eV), are expected to offer enhanced oxidative stability, which is beneficial for long-term device operation. The LUMO energies of the designed chromophores (−2.307 to −2.167 eV) are comparable to that of Spiro-OMeTAD, suggesting effective electron-blocking ability and suppressed charge recombination at the interface. Notably, the energy gaps (Δ*E*) of the BNPD series (2.682–3.769 eV) are either comparable to or lower than that of Spiro-OMeTAD, implying improved intramolecular charge transfer and potentially higher hole mobility. Overall, the results indicate that the BNPD chromophores demonstrate competitive and, in some cases, superior electronic characteristics compared with Spiro-OMeTAD.

A benchmarked study of BNP-based chromophores with our previously reported ND-series (naphthalene-1,5-diamine based HTMs) indicates that the BNP-based compounds show broader variations in energy gaps (2.682–4.050 eV), highlighting their greater structural flexibility for HTM design. Notably, BNPD3 presents the narrowest energy gap among the studied compounds, which is advantageous for efficient hole transport and interfacial charge extraction. Overall, the BNP-based chromophores demonstrate superior band-gap modulation, suggesting their greater potential as hole-transport materials in photovoltaic devices.^79^

The combination of tunable HOMO levels, suitable LUMO alignment, and reduced energy gaps suggests that these materials are promising alternative HTMs for perovskite solar cells, with potential advantages in charge transport efficiency and device stability.

**Fig. 7:**
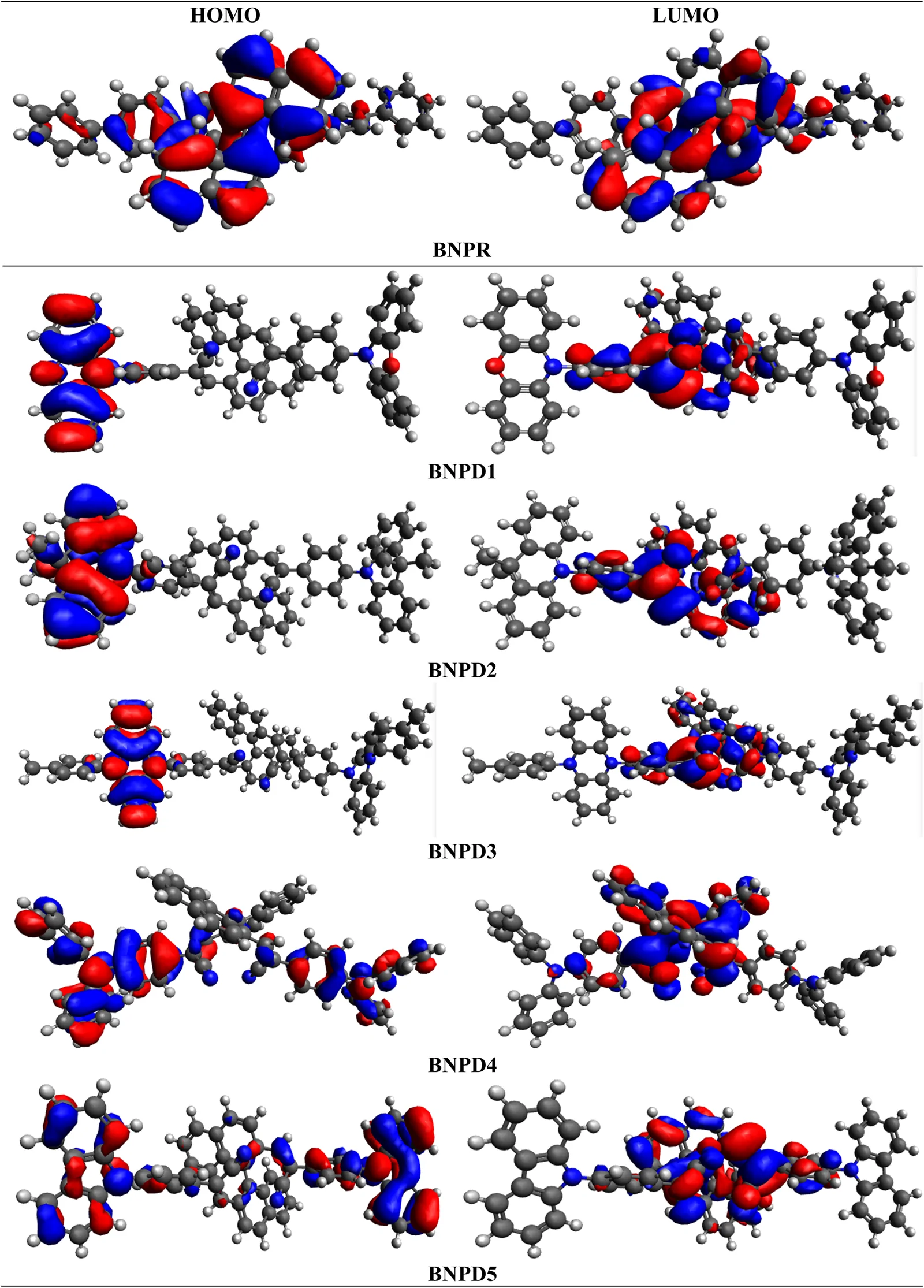
HOMOs and LUMOs of the reference (BNPR) and designed chromophores (BNPD1–BNPD5) illustrating the electronic cloud as different parts in a molecule.

In BNPD1–BNPD5, the electronic density in the HOMO is predominantly located over the donor units, while in BNPR, it is distributed over the acceptor region. In the LUMO, the electron density of BNPD1–BNPD5 and BNPR is predominantly located over the acceptor region (Fig. 7). This illustrates that the introduction of heterocyclic donors in the derivatives improves ICT compared with the reference chromophore, in which charge is located over the central core in both the HOMO and LUMO. Among the designed chromophores, BNPD3 appears to be a promising candidate for OSC applications in the future due to its reduced band gap and efficient photovoltaic properties.

### Global reactivity parameters (GRPs)

The energy values of the FMOs are crucial for estimating global reactivity parameters (GRPs). The GRPs comprise global softness (*σ*),^80^ global hardness (*η*),^81^ global electrophilicity index (*ω*),^82^ ionization potential (IP),^83^ chemical potential (*µ*),^84^ electron affinity (EA),^85^ and electronegativity (*X*).^86^ Chemical hardness is used to determine the stability and reactivity of the chromophores and can be calculated using eqn (S7), where *η* represents the resistance to changes in electronic charge density in the chromophore. The electron-accepting and electron-donating capabilities of chromophores are determined by electron affinity (EA) and ionization potential (IP), calculated using eqn (S5) and (S6), respectively. The ability of a species to attract electrons toward itself is referred to as electronegativity and is represented by *X.* The formula used to calculate electronegativity is given in eqn (S8).^81,86^ Electrophilicity (*ω*) is the capacity of a chromophore to accept electrons and can be calculated using the formula in eqn (S9).^82^ The chemical potential *µ* is calculated using eqn (S10).^85,87^ A direct relation is present between *µ*, global hardness (*η*), compound stability, and energy gaps, while the relationship is inverse for reactivity.^88^ Table S13 represents the calculated results of GRPs.

The ionization potential (IP) values for BNPR and BNPD1–BNPD5 are 6.264, 5.428, 5.638, 4.896, 5.671, and 6.076 eV, respectively. Their electron affinity (EA) values are 2.214, 2.236, 2.213, 2.214, 2.167, and 2.307 eV, respectively. Global softness (*σ*) and hardness (*η*) are important factors that are inversely related. A molecule having a high *σ* value and low *η* value shows less stability and *vice versa*.^89^ The designed chromophores exhibit lower global hardness (*η*) and higher global softness (*σ*) values compared to the reference chromophore. This attribute of the designed chromophores makes them more reactive and polarizing, which facilitates charge transfer in photovoltaic devices. The decreasing order of global softness (*σ*) and the increasing order of hardness (*η*) for the designed chromophores are as follows: BNPD3 > BNPD1 > BNPD2 > BNPD4 > BNPD5 > BNPR and BNPD3 < BNPD1 < BNPD2 < BNPD4 < BNPD5 < BNPR, respectively. BNPD1–BNPD5 exhibit higher Δ*N*_max_ values compared with BNPR due to the presence of end-capped donors. Among them, BNPD3 exhibits the lowest global hardness (*η*) value and the highest global softness (*σ*), global electrophilicity (*ω*), and maximum charge transfer index (Δ*N*_max_) values. These parameters indicate that BNPD3 is an effective chromophore among the series due to the stronger donor attached at both of its terminals. A study revealed that chromophores with high *σ*, *ω*, and Δ*N*_max_ values, while low *η* values are considered excellent candidates for solar cell devices.^90^

### UV-visible analysis

UV-visible analysis is performed to evaluate the optical properties of HTMs. These properties depend on several key factors, such as oscillator strength (*f*_os_), the absorption peak (*λ*_max_), and excitation energy (*E*, eV). Here, *λ*_max_ represents the wavelength corresponding to the photon energy required for a transition from the HOMO to the LUMO. *E* (eV) represents the excitation energy and *f*_os_ represents the probability of a transition.^91–95^ To explore these properties, TD-DFT calculations were performed to obtain the absorption spectra of the designed chromophores in both chloroform and the gas phase. A pictorial representation of the comparative UV-visible visible absorption spectra of the designed chromophores in both the solvent and gas phases is shown in Fig. S3. Additional information is provided in the SI Data (Tables S14–S25).

Table 2 presents the UV-Vis absorption data for BNPR and BNPD1–BNPD5 in the solvent phase and gas phase. The *λ*_max_ values for BNPD1–BNPD5 in the solvent phase range from 397.067 nm to 434.179 nm, while that for BNPR is 360.534 nm. The simulated value for the reference compound is in close agreement with reported experimental results (316–336 nm),^24^ which illustrates the suitable selection of the DFT level. These values represent a bathochromic shift for the designed chromophores, while a hypsochromic shift for the reference chromophore. Among the designed chromophores, BNPD4 exhibits the lowest excitation energy of 2.856 eV, the highest *λ*_max_ value of 434.179 nm, and a significant *f*_os_ value of 0.858. The observed decreasing order of *λ*_max_ in the solvent phase for the studied chromophores is as follows: BNPD4 > BNPD3 > BNPD2 > BNPD1 > BNPD5 > BNPR. This enhanced red shift in the solvent phase of intermolecular transitions highlights the importance of charge generation and solvent polarity, which improve π–π* transitions.^96^ The present study reveals that chromophores with lower excitation energy exhibit enhanced charge mobility. They exhibit an efficient transition from the ground state to the excited state with effective energy conversion. All the designed chromophores exhibit lower excitation energies than the reference chromophore.

**Table 2:** Wavelength (*λ*), excitation energy (*E*), oscillator strength (*f*_os_), and nature of the molecular orbital contributions of the BNPR1 and BNPD1–BNPD5 chromophores in the solvent and gas phases^a^

Phase	Chromophore	*λ*_max_ (nm)	*E* (eV)	*f*_os_	MO contributions
Solvent	BNPR	360.534	3.439	0.695	H−1 → L (90%)
	BNPD1	397.067	3.123	0.657	H−1 → L + 1 (83%)
	BNPD2	401.894	3.085	0.692	H−1 → L + 1 (85%)
	BNPD3	411.020	3.017	0.471	H−2 → L + 1 (10%), H−1 → L + 1 (73%)
	BNPD4	434.179	2.856	0.858	H−1 → L (27%), H → L (47%)
	BNPD5	393.826	3.148	0.634	H−1 → L (26%), H → L + 1 (33%)
Gas	BNPR	354.686	3.496	0.558	H−1 → L (91%), H−3 → L (2%)
	BNPD1	387.160	3.202	0.575	H−1 → L + 1 (85%)
	BNPD2	391.574	3.166	0.614	H−1 → L + 1 (85%)
	BNPD3	415.943	2.981	0.044	H−2 → L (14%), H−1 → L (82%)
	BNPD4	418.032	2.966	0.730	H−1 → L (15%), H → L (61%)
	BNPD5	390.674	3.174	0.360	H−4 → L (20%), H−1 → L (43%)

a MO = molecular orbital, H = HOMO, L = LUMO, *f*_os_ = oscillator strength, and wavelength = *λ* (nm).

Absorption spectra values for BNPD1–BNPD5 in the gas phase range from 387.160 to 418.032 nm, while that of BNPR is 354.686 nm. These values indicate a blue shift compared to the solvent phase due to the absence of solvent stabilization. Among the chromophores in the gas phase, BNPD4 exhibits the lowest excitation energy value of 2.966 eV and the highest *λ*_max_ value of 418.032 nm. The observed decreasing order of *λ*_max_ in the gaseous phase for the studied chromophores is: BNPD4 > BNPD3 > BNPD2 > BNPD1 > BNPD5 > BNPR. This analysis demonstrates that BNPD4 exhibits the lowest excitation energy and highest absorption among all the designed chromophores, making it an excellent chromophore for HTMs.

In comparison, the calculated TD-DFT results reveal that the BNPD derivatives show systematically red-shifted absorption maxima relative to BNPR and Spiro-OMeTAD. In the solvent phase, BNPD3 and BNPD4 exhibit *λ*_max_ values of 411.020 and 434.179 nm, respectively, with reduced excitation energies (3.017–2.856 eV), indicating enhanced ICT character. This bathochromic shift arises from extended π-conjugation and stronger donor–acceptor interactions, which are less pronounced in Spiro-OMeTAD due to its highly twisted molecular framework.

The optical absorption and excited-state properties of BNPR and its derivatives were benchmarked against Spiro-OMeTAD to evaluate their suitability for optoelectronic and photovoltaic applications. Spiro-OMeTAD typically exhibits a strong absorption band in the ultraviolet region with a reported absorption maximum (*λ*_max_) around 380–390 nm, corresponding to an optical energy gap of approximately 3.0–3.1 eV,^97^ which is sufficient for efficient hole extraction while minimizing parasitic absorption in perovskite solar cells.

Furthermore, both gas- and solvent-phase results show consistent trends, with the BNPD chromophores maintaining lower excitation energies than Spiro-OMeTAD, suggesting improved light-harvesting capability without compromising electron-blocking behavior. The solvent-induced stabilization observed for the BNPD compounds further supports their stronger excited-state polarization compared to Spiro-OMeTAD, which typically shows a limited solvatochromic response.

Overall, while Spiro-OMeTAD remains an effective and stable benchmark HTM, the BNPD chromophores demonstrate broader absorption, lower excitation energies, and stronger ICT features, which are advantageous for next-generation HTMs aiming to enhance photocurrent density and interfacial charge transfer in optoelectronic devices.

Further, a benchmarked analysis of the BNP-based chromophores with our reported ND-series showed that the BNP derivatives have a larger absorption range (360 to 434 nm), with BNPD4 exhibiting the largest red shift, which signifies increased π-conjugation and stronger acceptor–donor interaction. Though the BNP series has a greater ICT character, parasitic light harvesting can occur due to its prolonged absorption into the visible region.^79^

### Binding energy

The binding energy of electrons and holes acts as an important factor in the evaluation of optoelectronic properties. It further contributes to the evaluation of the exciton dissociation capacity of chromophores. Binding energy is directly proportional to the energy gap (*E*_gap_) and the coulombic interactions between holes and electrons. It has an inverse relationship with the exciton dissociation rate. When the binding energy decreases, the coulombic force of attraction between the hole–electron pair is reduced, thereby increasing exciton dissociation.^98–100^ The following eqn (S11) is used for the determination of the *E*_b_ values.^101^ According to the this equation, the energy gap (*E*_gap_) represents the LUMO/HOMO energy gap, while *E*_opt_ is the least energy required for the transfer of an electron from the ground state (S_0_) to the excited state (S_1_). Table S26 presents the binding energy (*E*_b_) values for BNPR and BNPD1–BNPD5. The above-mentioned table shows that BNPD1 has the lowest *E*_b_ (0.069 eV) among the designed chromophores, while BNPD5 exhibits the highest value (0.595 eV). Thus, BNPD1 exhibits the weakest coulombic interactions between holes and electrons, and the highest dissociation potential. The binding energy of the studied chromophores decreases in the following order (in eV): BNPD4 (0.648) > BNPD5 (0.621) > BNPR (0.611) > BNPD2 (0.340) > BNPD3 (0.335) > BNPD1 (0.069). Their lower binding energy values illustrate higher exciton dissociation rates with greater ICT, which supports their potential as promising HTMs.

### Electron–hole analysis

**Fig. 8:**
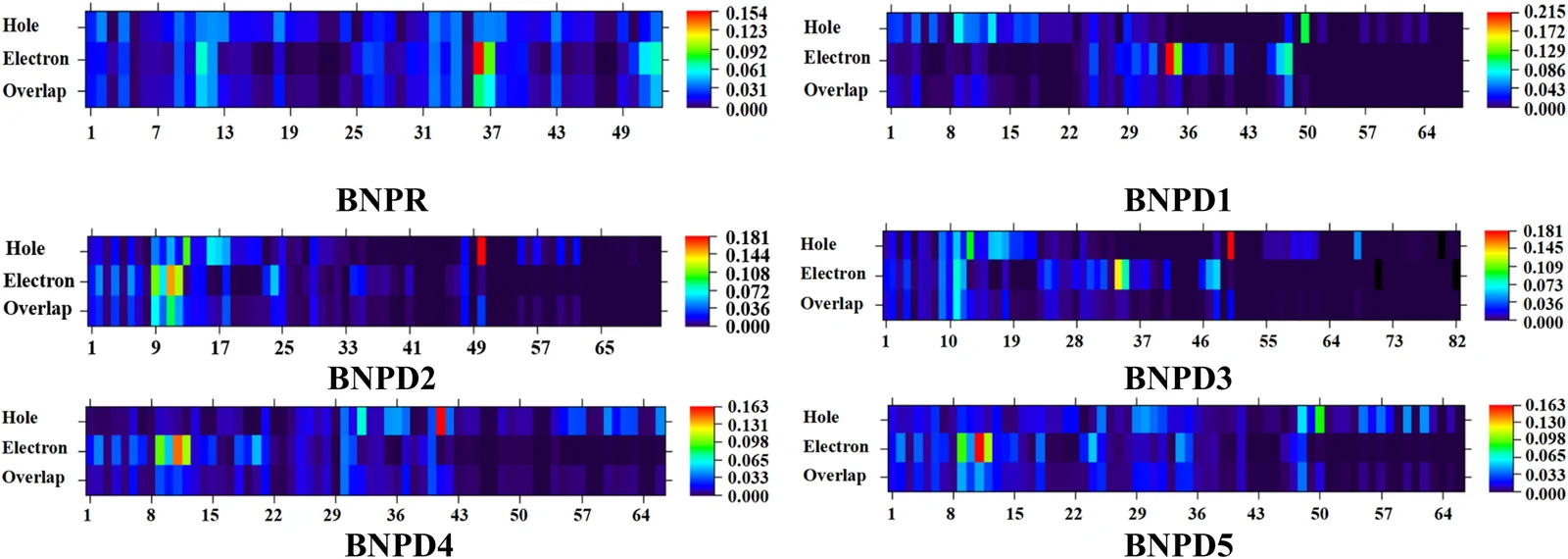
Pictorial representation of electron–hole transport analysis.

Molecular interactions can be analyzed to gain deeper insight into the nature of electronic excitations in a molecule.^102^ In the designed chromophores (BNPD1–BNPD5), holes from different atoms of the terminal donors permit effective charge transfer to the central acceptor group. This highlights the attribute of the electron-withdrawing capability of the central acceptors. According to the current analysis, all the studied chromophores exhibit uniform charge transfer pathways. This uniformity is due to the majority of the charge transfer density being present within the central electron-withdrawing acceptor unit. This study strongly supports efficient intramolecular charge transfer from the terminal donors to the central acceptor moiety. A pictorial representation of the electron–hole transport analysis of the BNPR and BNPD1–BNPD5 chromophores is shown in Fig. 8.

#### Density of states (DOS)

The density of states (DOS) analysis is a useful tool for analyzing the charge distribution between the LUMO and HOMO of the studied chromophores. DOS also helps to validate the results of FMO analysis. Furthermore, DOS is also useful for the determination of intermolecular charge transfer. In the current study, this analysis was performed to predict the percentage contribution of each moiety in a molecule to the overall electronic charge distribution. In DOS maps, the energy values on the left side represent the HOMO, while the energy values on the right side represent the LUMO. The gap between these two regions corresponds to the energy gap and is comparable to the FMO energy gap.^103–105^ To interpret the DOS analysis, the designed chromophores are divided into the donor end-capped and the acceptor (core unit). Pictorial representations of these fragments are shown as green and red lines in Fig. S4.

The current study indicates the distribution of electrons from the donor state (HOMO) to the acceptor state (LUMO).^106^ According to this study, the acceptor contributes 14.6, 14.3, 13.8, 12.5, 11.6, and 12.8% to the LUMO of the BNPR and BNPD1–BNPD5 chromophores, respectively. For the HOMO, the corresponding contributions are 15.2, 100, 99.9, 100, 89.0, and 90.8% for the BNPR and BNPD1–BNPD5 chromophores, respectively. Similarly, the donor contributes 85.4, 85.7, 86.2, 87.5, 88.4, and 87.2% to the LUMO for the BNPR and BNPD1–BNPD5 chromophores, respectively. For the HOMO, the corresponding contributions are 84.8, 0.0, 0.1, 0, 11.0, and 9.2% for the BNPR and BNPD1–BNPD5 chromophores, as presented in Table S27. These findings clearly demonstrate that structural modification significantly affects the charge density distribution in the HOMO and LUMO. These result will be helpful for fine-tuning the electronic characteristics of the studied chromophores.

#### Transition density matrix (TDM)

Transition density matrix (TDM) analysis is essential for evaluating the extent of charge transfer within chromophores. To obtain the TDM heat maps of the studied chromophores, the M06/6-311G(d,p) level of theory was used in TD-DFT calculations. This discussion analyzes the electronic charge excitation and the intermolecular interactions between the acceptor and donor units in their excited states. TDM analysis provides insights into the localization and delocalization of electron–hole pairs.^104,107^ TDM heat maps also support the charge transfer patterns observed in the FMO and DOS analyses. The studied chromophores are divided into donor and acceptor units for TDM investigation to analyze the transfer of charge density. The heat maps in Fig. S5 illustrate bright dots as the distribution of charge flow, excluding hydrogen atoms due to their minor contribution. The heat maps of the studied chromophores exhibit a diagonal pattern, indicating charge transfer. In the BNPR and BNPD1–BNPD5 chromophores, the acceptor region indicated by a red line exhibits a higher electronic cloud. The donor region, denoted by a yellow line, shows a smaller but significant charge density. These regions effectively help to understand the transfer of electrons from donors to acceptors. The results of the TDM heat maps indicate the dissociation of effective excitons due to excitation.

To investigate the charge-transfer behavior and the ability of the designed HTMs to dissociate excitons, the following parameters were calculated. The efficiency of charge separation was evaluated by using the electron–hole separation distance (*D*), the coulombic attraction energy (*E*_Coul_), overlap index (*S*_r_), the centroid displacement (*t*) and delocalization indices (HDI and EDI) of the designed compounds (Table S28).

The separation distance between the electron–hole pair of reference molecule BNPR is small (*D* = 1.147 Å), while the coulombic attraction energy is high (*E*_Coul_ = 3.27 eV), and the overlap index is large (*S*_r_ = 0.678), indicating strong interactions between the electron–hole and poor exciton dissociation in BNPR. For BNPD5, the smallest separation distance (0.887 Å), high *E*_Coul_ (3.09 eV), and high *S*_r_ (0.626) indicate that the excitation in the molecule is localized and the charge-transfer potential is not significant. The charge-transfer characteristics for the latter are markedly better, with BNPD3 showing the greatest improvement. The separation between the electron and hole (8.531 Å) and the coulombic attraction energy (1.64 eV) of BNPD3 are the greatest and the lowest among the molecules, respectively, showing that the molecule has the best intramolecular charge transfer ability and exciton dissociation ability. BNPD1 (*D* = 8.187 Å, *E*_Coul_ = 1.67 eV, *S*_r_ = 0.072) and BNPD2 (*D* = 8.054 Å, *E*_Coul_ = 1.70 eV, *S*_r_ = 0.080) also demonstrate excellent charge separation with weak electron–hole interactions. However, with a relatively long absorption wavelength, BNPD4 has a moderate electron–hole separation (5.517 Å) and a high overlap index (0.445), which means that the charge-transfer efficiency is comparatively low.

In addition, the positive *t* values of BNPD1–BNPD4 support the electron migration from one direction after excitation, while the negative values of BNPR and BNPD5 show the localized nature of the excitations. Overall, the TDM results demonstrate that donor engineering can lead to improved charge separation and reduced exciton binding. From these parameters, the charge-transfer performance in entitled chromophores can be predicted: The results showed that BNPD3 is the promising HTM for efficient perovskite solar cells, with BNPD1 and BNPD2 having similar performance.

## Conclusion

In the current study, binaphthyl acrylonitrile-based D–A–D hole-transport materials were designed and quantum chemically analyzed to determine the impact of the electron-donating units. By introducing heterocyclic donors with the binaphthyl acrylonitrile core, the optical and electronic properties were efficiently tuned. The CatBoost model exhibited good prediction performance for *E*_gap_ values, as given by the high test *R*^2^ and CV *R*^2^ values. This implies that the model is stable and can be generalized, thus making reliable predictions. SHAP analysis explains that the *E*_gap_ is driven by the combined effect of topological, electronic and structural flexibility features. Moreover, the synthetic accessibility scores of the investigated chromophores lie in the range of 2.53–4.46. BNPD2 is identified as the simplest chromophore to synthesize among the investigated chromophores, with the lowest SAS of 3.10.

All the designed derivatives exhibit energy gaps within the range of 3 to 4 eV and absorption spectra within the UV region (387.160 to 418.032 nm), supporting their utilization as HTMs. These chromophores exhibit low exciton binding energies of 0.648–0.069 eV, which indicates increased rates of exciton dissociation and effective ICT. TDM, FMO, and DOS analyses also revealed efficient charge transfer between the donors in the HOMO towards the core (binaphthyl acrylonitrile) in the LUMO for the designed derivatives. The comparative analysis of molecular orbitals energies and UV-Vis absorption properties reveals that the designed BNPD derivatives exhibit comparable electronic characteristics to the benchmark Spiro-OMeTAD, including deeper HOMO levels and reduced HOMO–LUMO gaps, which promote efficient hole extraction and charge transport. Molecular engineering effectively promotes electron–hole separation while reducing coulombic attraction and charge overlap. Among the investigated molecules, BNPD3 exhibits the most efficient charge-transfer characteristics, followed by BNPD1 and BNPD2, highlighting their potential as high-performance hole-transport materials for perovskite solar cells. Additionally, the red-shifted absorption maxima and lower excitation energies of the BNPD chromophores indicate enhanced ICT compared with Spiro-OMeTAD. These findings suggest that the designed derivatives are promising alternatives to the conventional Spiro-OMeTAD for advanced optoelectronic and photovoltaic applications.

## Conflicts of interest

There are no conflicts to declare.

## Supplementary Material

RA-016-D6RA04301A-s001

## Data Availability

All data generated or analyzed during this study are included in this published article and its supplementary information (SI) files. Supplementary information is available. See DOI: https://doi.org/10.1039/d6ra04301a.
