# Supplementary material for: Strategic modulation of heterocyclic donors in binaphthyl acrylonitrile-based hole transport materials for perovskite solar cells: a DFT and machine learning investigation
Source: RSC Adv. 2026 Aug 11;16(40):46571–89. doi: 10.1039/d6ra04301a (PMC13458690; doi:10.1039/d6ra04301a)
Supplement: Supplementary file 1 [file RA-016-D6RA04301A-s001.pdf]

Supplementary Information (SI) for RSC Advances.
This journal is © The Royal Society of Chemistry 2026

Strategic Modulation of Heterocyclic Donors in Binaphthyl Acrylonitrile-Based Hole Transport Materials for Perovskite Solar Cells: A DFT and Machine Learning Investigation

Iqra Shafiq,[1] Asma Irshad,[1] Muqadas Javed,[1] Samia Majeed,[1] Muhammad Adnan Asghar, *[2], Nadia Ayari,[3,] Ke Chen,*[4]

[1]Institute of Chemistry, Khwaja Fareed University of Engineering & Information Technology, Rahim Yar Khan, 64200, Pakistan

[2]Department of Chemistry, Division of Science and Technology, University of Education Lahore, 54000, Pakistan

[3]Department of Physics, College of Science, Northern Border University, Arar, 91431 Saudi Arabia

[4]Department of Infectious Diseases, The Affiliated Hospital of Southwest Medical University, Luzhou 646000, China

*Corresponding author's E-mail addresses:

Dr. Muhammad Adnan Asghar (adnan.muhammad@ue.edu.pk)

Dr. Ke Chen: chen_ke@swmu.edu.cn

Table S1: Cartesian coordinates of **BNPR** compound.

Atoms	X-axis	Y-axis	Z-axis
C	-3.11403	2.251497	3.101943
C	-3.80485	3.143608	2.328953
C	-3.29261	3.522868	1.072712
C	-2.13142	2.973234	0.599209
H	-3.47972	1.969046	4.08563
H	-4.73469	3.574115	2.686444
H	-3.82188	4.260133	0.476924
C	0.056915	0.342453	3.037759
C	-1.13742	0.827728	3.472716
C	-1.89983	1.68682	2.649473
C	-1.41024	2.016381	1.356123
C	-0.21921	1.393242	0.866837
C	0.524721	0.587617	1.719413
H	0.66087	-0.28337	3.688965
H	-1.50463	0.588985	4.466933
C	1.849369	0.032201	1.426449
C	2.388454	-0.59103	0.363064
C	3.832678	-0.92356	0.315493
C	4.541208	-0.76921	-0.87685
C	4.522388	-1.36073	1.446407
C	5.901171	-1.01827	-0.92868

H	4.023672	-0.42806	-1.77017
C	5.881339	-1.61277	1.389861
H	3.983593	-1.53531	2.37322
C	6.599831	-1.44256	0.203502
H	6.43582	-0.8563	-1.86008
H	6.38986	-1.98259	2.275556
C	8.049944	-1.71096	0.14747
C	8.629965	-2.28239	-0.98787
C	8.877445	-1.39803	1.228802
C	9.992537	-2.53284	-1.04006
H	8.000932	-2.5559	-1.83043
C	10.24023	-1.64733	1.176409
H	8.450439	-0.93131	2.112206
C	10.80346	-2.21602	0.041715
H	10.42243	-2.98557	-1.92825
H	10.86738	-1.38866	2.024017
H	11.87032	-2.41169	0.000695
C	1.666777	-0.97723	-0.80666
N	1.173528	-1.36877	-1.77472
C	-0.61937	1.212194	-1.59007
C	1.39429	2.343834	-0.8279
C	-0.22442	1.464874	-2.92877
C	1.806785	2.514336	-2.17895
C	0.966144	2.057274	-3.21814
H	-0.88286	1.140715	-3.72985
H	1.275399	2.207381	-4.24877
C	2.20782	2.903095	0.188846
C	3.028897	3.167628	-2.45873
C	3.808636	3.660273	-1.44908
C	3.382217	3.538202	-0.11194
H	3.33054	3.276182	-3.49721
H	4.746304	4.158444	-1.67359
H	3.987797	3.957416	0.685296
C	-1.93907	0.593814	-1.43828
C	-2.39294	-0.42189	-0.67803
C	-3.83401	-0.75019	-0.60333
C	-4.27093	-2.07014	-0.47845
C	-4.79106	0.266091	-0.63512
C	-5.62082	-2.36476	-0.42193
H	-3.54396	-2.87688	-0.44377
C	-6.14067	-0.03267	-0.57789
H	-4.47158	1.304361	-0.66744
C	-6.58365	-1.35348	-0.47386
H	-5.93602	-3.40224	-0.3596
H	-6.86438	0.777336	-0.57379

C	-8.02341	-1.66999	-0.40613
C	-8.95248	-0.93927	-1.15096
C	-8.49149	-2.70777	0.403775
C	-10.3051	-1.23703	-1.08884
H	-8.60696	-0.14376	-1.80537
C	-9.84416	-3.0047	0.467229
H	-7.78873	-3.27304	1.009574
C	-10.7566	-2.2708	-0.2791
H	-11.0101	-0.66318	-1.68245
H	-10.1885	-3.80948	1.109384
H	-11.8156	-2.50373	-0.23004
C	-1.55333	-1.2731	0.101734
N	-0.96165	-2.03833	0.732921
H	1.887968	2.829199	1.223231
H	2.546633	0.181385	2.252987
H	-2.6939	1.044051	-2.08541
C	0.187996	1.634391	-0.54099
H	-1.74859	3.282321	-0.36815

Table S2: Cartesian coordinates of **BNPD1** compound.

Atoms	X-axis	Y-axis	Z-axis
C	-2.93256	-3.76591	-2.08849
C	-3.73133	-4.21048	-1.0711
C	-3.33136	-4.01997	0.266135
C	-2.17141	-3.35373	0.557733
H	-3.21154	-3.9305	-3.12594
H	-4.66123	-4.7258	-1.289
H	-3.94773	-4.40722	1.071673
C	0.328505	-2.08105	-2.57517
C	-0.85181	-2.69537	-2.86017
C	-1.71903	-3.09344	-1.81797
C	-1.34213	-2.84031	-0.47038
C	-0.15556	-2.09275	-0.19661
C	0.683217	-1.73564	-1.24513
H	1.008656	-1.80541	-3.37633
H	-1.13244	-2.90907	-3.88777
C	1.99505	-1.09941	-1.09663
C	2.428223	-0.04008	-0.3845
C	3.864064	0.288914	-0.27229
C	4.313986	1.607986	-0.21313
C	4.824395	-0.7203	-0.20416
C	5.657902	1.90956	-0.10826
H	3.598	2.424062	-0.26327
C	6.171406	-0.43052	-0.09555

H	4.511456	-1.76118	-0.21017
C	6.621092	0.894639	-0.03798
C	8.490911	2.485934	-0.23501
C	8.974363	0.185146	-0.10019
C	9.339489	2.574946	-1.3362
C	9.815166	0.319047	-1.20165
C	1.56502	0.855745	0.316514
N	0.953706	1.65639	0.881811
C	-0.63511	-0.87285	1.939484
C	1.348329	-2.27506	1.811947
C	-0.25861	-0.51123	3.259965
C	1.747578	-1.82847	3.101336
C	0.915368	-0.9293	3.805426
H	-0.91601	0.148174	3.819831
H	1.21357	-0.60387	4.798117
C	2.138562	-3.26722	1.179656
C	2.944885	-2.31889	3.670441
C	3.704896	-3.24764	3.014492
C	3.281908	-3.74133	1.764753
H	3.241712	-1.94848	4.648079
H	4.621715	-3.61986	3.460279
H	3.866868	-4.50607	1.263098
C	-1.94224	-0.36471	1.51779
C	-2.41352	0.171754	0.374855
C	-3.85638	0.464155	0.204107
C	-4.282	1.57075	-0.53304
C	-4.81602	-0.37969	0.770393
C	-5.63202	1.846858	-0.6737
H	-3.54858	2.2268	-0.99263
C	-6.16494	-0.10281	0.631993
H	-4.50225	-1.27555	1.298339
C	-6.57411	1.012701	-0.08812
C	-8.64886	1.986738	0.763392
C	-8.69568	0.650975	-1.24823
C	-10.0289	2.180272	0.631535
C	-10.0747	0.876714	-1.33042
C	-1.60549	0.529355	-0.74542
N	-1.04268	0.892485	-1.68627
H	1.823964	-3.66176	0.218698
H	2.768597	-1.58673	-1.69284
H	-2.68371	-0.42732	2.316133
C	0.176804	-1.72698	1.203116
H	-1.87491	-3.22092	1.59343
N	-7.96254	1.292816	-0.24153
N	7.991797	1.195068	0.099848

O	-10.7201	1.720782	-0.4616
O	9.720873	1.433547	-1.99701
C	-10.8376	0.271393	-2.30712
C	-8.11085	-0.19762	-2.18236
C	-8.01579	2.506725	1.887425
C	-10.7461	2.863501	1.591298
C	9.150984	-0.89189	0.7589
C	10.77466	-0.63564	-1.49071
C	8.209492	3.634259	0.494285
C	9.841637	3.793449	-1.75771
C	8.700217	4.862162	0.07343
C	9.501234	4.942412	-1.05895
C	10.10292	-1.85953	0.472066
C	10.90267	-1.73768	-0.65811
C	-10.2416	-0.57183	-3.23963
C	-8.87899	-0.80153	-3.17279
C	-10.1019	3.383937	2.709428
C	-8.7377	3.203225	2.85151
H	-10.8461	-1.04029	-4.00853
H	-8.39642	-1.45496	-3.89211
H	-11.9026	0.480125	-2.32396
H	-11.8141	2.983609	1.43978
H	-8.21795	3.602788	3.716051
H	-10.6708	3.925003	3.457652
H	10.22373	-2.70856	1.136185
H	11.64775	-2.49383	-0.88181
H	11.40852	-0.49583	-2.36004
H	10.49586	3.819903	-2.62279
H	8.460035	5.757894	0.635948
H	9.884952	5.902452	-1.38762
H	7.594538	3.556584	1.385457
H	5.954962	2.950236	-0.09419
H	-5.96812	2.711471	-1.23794
H	-7.04251	-0.38215	-2.13647
H	-6.94569	2.369966	2.004004
H	-6.91481	-0.75899	1.065244
H	8.523631	-0.97	1.641663
H	6.876247	-1.25114	-0.05359

Table S3: Cartesian coordinates of **BNPD2** compound.

Atoms	X-axis	Y-axis	Z-axis
C	3.143684	3.665317	-2.21461
C	3.876125	4.250386	-1.21866
C	3.379609	4.2598	0.099544

C	2.191971	3.648636	0.401126
H	3.496913	3.674645	-3.24244
H	4.826797	4.723691	-1.44221
H	3.942414	4.759136	0.882294
C	-0.11492	1.972307	-2.68543
C	1.1004	2.513765	-2.97026
C	1.902202	3.048795	-1.93697
C	1.427133	2.996399	-0.59803
C	0.20811	2.308466	-0.30339
C	-0.57117	1.828247	-1.3486
H	-0.74694	1.598537	-3.4863
H	1.456579	2.569915	-3.99516
C	-1.92066	1.270773	-1.21344
C	-2.47522	0.365323	-0.38665
C	-3.92806	0.088463	-0.39283
C	-4.60135	-0.17695	0.799225
C	-4.68479	0.106217	-1.56374
C	-5.97032	-0.36289	0.833583
H	-4.04442	-0.22913	1.731983
C	-6.05266	-0.08445	-1.54348
H	-4.1931	0.242767	-2.52301
C	-6.73879	-0.29436	-0.33722
C	-8.80074	-0.96439	0.84802
C	-8.88224	-0.66523	-1.50798
C	-9.50102	-2.1713	0.752348
C	-9.58589	-1.86321	-1.66881
C	-1.74313	-0.40075	0.571468
N	-1.24079	-1.09557	1.345204
C	0.587406	1.378745	1.984996
C	-1.37986	2.756832	1.607546
C	0.183423	1.216559	3.335126
C	-1.80777	2.507887	2.941392
C	-0.99507	1.723178	3.789506
H	0.822503	0.640121	3.998211
H	-1.31458	1.552865	4.813846
C	-2.15934	3.635455	0.814764
C	-3.01536	3.078098	3.405371
C	-3.76406	3.888963	2.597938
C	-3.31885	4.182097	1.293702
H	-3.32993	2.863528	4.423313
H	-4.69055	4.321013	2.96235
H	-3.89812	4.852364	0.666592
C	1.88952	0.794587	1.653662
C	2.326136	0.056405	0.614777
C	3.760347	-0.27673	0.449959

C	4.156028	-1.50031	-0.09488
C	4.741181	0.648542	0.815038
C	5.498649	-1.80285	-0.24378
H	3.404369	-2.22315	-0.39862
C	6.084163	0.343885	0.669231
H	4.447576	1.625965	1.187071
C	6.464356	-0.882	0.140459
C	8.532663	-1.83225	1.00866
C	8.487651	-0.82027	-1.2145
C	9.894354	-2.14029	0.881686
C	9.848696	-1.09533	-1.40797
C	1.478357	-0.47806	-0.4013
N	0.88016	-0.98837	-1.24724
H	-1.82447	3.881331	-0.18774
H	-2.6196	1.710443	-1.92738
H	2.6462	0.992859	2.414882
C	-0.19447	2.129454	1.115245
H	1.822018	3.672284	1.421213
N	7.848072	-1.18717	-0.02607
N	-8.12705	-0.49265	-0.31389
C	10.42961	-0.70445	-2.61286
C	7.762777	-0.17149	-2.22443
C	7.85272	-2.17498	2.18596
C	10.52035	-2.78562	1.94645
C	-8.98929	0.35778	-2.44288
C	-10.3726	-2.01509	-2.80632
C	-8.83755	-0.2071	2.013192
C	-10.2083	-2.61029	1.867056
C	-9.53225	-0.67176	3.11902
C	-10.2142	-1.87732	3.046463
C	-9.76236	0.178645	-3.57941
C	-10.4515	-1.01124	-3.76237
C	9.717276	-0.06227	-3.61198
C	8.371406	0.20238	-3.40776
C	9.851704	-3.12696	3.110301
C	8.505288	-2.81457	3.223061
H	10.20757	0.225555	-4.53599
H	7.784938	0.702996	-4.17206
H	11.48365	-0.91123	-2.7774
H	11.57535	-3.03197	1.862327
H	7.953093	-3.06832	4.122711
H	10.37617	-3.62935	3.916249
H	-9.84036	0.97675	-4.31065
H	-11.0663	-1.15558	-4.64507
H	-10.937	-2.92931	-2.95675

H	-10.7672	-3.53907	1.8232
H	-9.55492	-0.08156	4.029439
H	-10.7674	-2.2439	3.905326
H	-6.43989	-0.57573	1.785418
H	5.811844	-2.7559	-0.66016
H	6.709628	0.041328	-2.07826
H	6.799797	-1.93656	2.286696
H	6.851101	1.062469	0.944034
H	-8.47291	1.296454	-2.2661
H	-6.58792	-0.09603	-2.48462
H	-8.32979	0.752235	2.041036
C	-9.44527	-2.91539	-0.57596
C	10.7029	-1.7996	-0.36264
C	-8.07374	-3.60345	-0.71176
H	-7.24158	-2.89665	-0.64889
H	-7.94995	-4.34563	0.085029
H	-8.01035	-4.11843	-1.67709
C	-10.5171	-3.99164	-0.67569
H	-11.5274	-3.57759	-0.58796
H	-10.4415	-4.52039	-1.63051
H	-10.3813	-4.74219	0.108661
C	11.8659	-0.87778	0.035164
H	11.48605	0.054131	0.467694
H	12.51565	-1.35625	0.774737
H	12.48428	-0.62358	-0.83144
C	11.25937	-3.09819	-0.96656
H	11.86259	-2.89413	-1.85684
H	11.89617	-3.62692	-0.25026
H	10.4422	-3.76812	-1.25504

Table S4: Cartesian coordinates of **BNPD3** compound.

Atoms	X-axis	Y-axis	Z-axis
C	2.495233	2.149307	-3.98568
C	3.318969	3.179389	-3.62259
C	2.978695	3.991968	-2.52332
C	1.853395	3.732649	-1.7878
H	2.726858	1.53054	-4.84866
H	4.222042	3.38498	-4.18823
H	3.612951	4.832817	-2.25984
C	-0.73463	0.6433	-2.97845
C	0.417474	0.86101	-3.66895
C	1.315322	1.872269	-3.25834
C	1.00004	2.647756	-2.10958
C	-0.15251	2.323579	-1.32868

C	-1.02914	1.343286	-1.7779
H	-1.43969	-0.11029	-3.31838
H	0.649571	0.281465	-4.55817
C	-2.31988	1.007667	-1.1722
C	-2.73069	0.812885	0.098059
C	-4.1574	0.644004	0.441246
C	-4.56392	-0.16471	1.503801
C	-5.15486	1.301468	-0.28056
C	-5.89856	-0.33027	1.818934
H	-3.81941	-0.6918	2.094488
C	-6.49302	1.145413	0.026385
H	-4.88033	1.973145	-1.08938
C	-6.89799	0.325057	1.087633
C	-8.65935	-0.93442	2.207703
C	-9.24202	0.525406	0.450398
C	-9.23753	-2.01402	1.52713
C	-9.82871	-0.53107	-0.25871
C	-1.84824	0.729948	1.2177
N	-1.2215	0.604064	2.179765
C	0.490096	2.95428	1.004977
C	-1.53328	3.912326	0.053646
C	0.228345	3.66316	2.206295
C	-1.81724	4.543891	1.295699
C	-0.90771	4.394914	2.366585
H	0.943237	3.578772	3.020072
H	-1.11769	4.894931	3.307967
C	-2.40304	4.16825	-1.03543
C	-2.98099	5.335477	1.425728
C	-3.82009	5.52995	0.363443
C	-3.5138	4.953926	-0.88523
H	-3.18882	5.793957	2.388945
H	-4.71141	6.139266	0.473391
H	-4.16351	5.137348	-1.73542
C	1.767432	2.23771	0.976403
C	2.131891	1.014562	0.544387
C	3.554961	0.603056	0.499621
C	3.928081	-0.71486	0.773789
C	4.54374	1.527207	0.1548
C	5.25876	-1.09204	0.73198
H	3.168085	-1.4481	1.027813
C	5.876263	1.149485	0.114863
H	4.262868	2.541646	-0.11398
C	6.235336	-0.1596	0.403985
C	8.374593	-0.52918	1.515388
C	8.113783	-1.06249	-0.86055

C	9.713451	-0.96571	1.465107
C	9.453449	-1.49684	-0.90884
C	1.220318	0.005415	0.111601
N	0.57382	-0.89281	-0.2188
H	-2.17771	3.736952	-2.0055
H	-3.10637	0.883405	-1.91827
H	2.583044	2.820504	1.408275
C	-0.39443	3.057695	-0.06162
H	1.603082	4.373215	-0.94817
N	7.602726	-0.55306	0.343508
N	-8.26019	0.19206	1.42943
C	9.95947	-1.99169	-2.10335
C	7.334353	-1.14202	-2.00662
C	7.854013	-0.07804	2.720436
C	10.47665	-0.93533	2.624675
C	-9.58942	1.837553	0.174527
C	-10.6858	-0.23608	-1.31865
C	-8.46074	-0.99024	3.577434
C	-9.51094	-3.18558	2.23242
C	-8.75063	-2.15237	4.278527
C	-9.25429	-3.25026	3.594226
C	-10.4568	2.125878	-0.86983
C	-10.9816	1.084596	-1.62334
C	9.1671	-2.06362	-3.24787
C	7.856034	-1.63969	-3.19978
C	9.941993	-0.47885	3.828422
C	8.632433	-0.05022	3.876529
H	9.59189	-2.45388	-4.16688
H	7.222712	-1.68841	-4.0794
H	10.99085	-2.3254	-2.14095
H	11.5071	-1.27194	2.586976
H	8.198083	0.308904	4.803657
H	10.5642	-0.46575	4.717248
H	-10.7114	3.154917	-1.09925
H	-11.6487	1.297371	-2.45283
H	-11.1236	-1.0398	-1.9006
H	-9.92757	-4.04366	1.716244
H	-8.57361	-2.2029	5.347367
H	-9.46999	-4.1704	4.128092
H	5.555076	-2.11385	0.951414
H	6.302794	-0.80828	-1.97038
H	6.823767	0.259422	2.758518
H	6.648848	1.86119	-0.16141
N	10.2442	-1.41323	0.246469
N	-9.548	-1.83982	0.167781

C	11.59285	-1.87506	0.201991
C	12.62039	-0.99116	-0.09529
C	11.88056	-3.20864	0.46228
C	13.93121	-1.44315	-0.1328
H	12.38151	0.050621	-0.29115
C	13.19292	-3.65243	0.422381
H	11.06655	-3.88881	0.698811
C	14.23747	-2.77869	0.121866
H	14.73449	-0.7472	-0.36165
H	13.41572	-4.69608	0.630416
C	-10.0871	-2.94222	-0.56873
C	-11.4238	-3.30354	-0.42845
C	-9.26617	-3.65214	-1.42929
C	-11.9275	-4.37197	-1.14896
H	-12.0596	-2.7377	0.248099
C	-9.7804	-4.72343	-2.14858
H	-8.22591	-3.35763	-1.53065
C	-11.1136	-5.10016	-2.01932
H	-12.9727	-4.6515	-1.0397
H	-9.13308	-5.27658	-2.82408
C	15.65008	-3.27105	0.051093
H	15.82617	-4.0833	0.762046
H	15.87917	-3.65991	-0.94796
H	16.36426	-2.46991	0.260041
C	-11.6708	-6.26436	-2.77848
H	-10.9758	-6.61294	-3.54637
H	-11.8773	-7.1065	-2.10839
H	-12.6162	-6.00447	-3.26509
H	-8.04134	-0.12102	4.076176
H	-6.16389	-0.98468	2.640196
H	-9.13959	2.628129	0.768577
H	-7.22924	1.677691	-0.56349

Table S5: Cartesian coordinates of **BNPD4** compound.

Atoms	X-axis	Y-axis	Z-axis
C	-3.10773	-1.99044	-3.57012
C	-3.83325	-2.98629	-2.97598
C	-3.34981	-3.59848	-1.80298
C	-2.1813	-3.17592	-1.22776
H	-3.45151	-1.52662	-4.49094
H	-4.76904	-3.31916	-3.41337
H	-3.9079	-4.41382	-1.35299
C	0.110336	-0.214	-3.13302
C	-1.08832	-0.58112	-3.66107

C	-1.8858	-1.5509	-3.01223
C	-1.42397	-2.12058	-1.79456
C	-0.22462	-1.62938	-1.19047
C	0.553074	-0.70322	-1.87482
H	0.739366	0.50191	-3.6549
H	-1.43384	-0.15903	-4.60068
C	1.878072	-0.22878	-1.47032
C	2.411598	0.179901	-0.30088
C	3.85639	0.464913	-0.16709
C	4.323951	1.466328	0.68776
C	4.799302	-0.27297	-0.88742
C	5.673226	1.739967	0.798598
H	3.61749	2.04107	1.280369
C	6.149762	0.002762	-0.79038
H	4.470953	-1.08757	-1.52676
C	6.609796	1.016657	0.054263
C	1.658305	0.402552	0.891294
N	1.140536	0.654578	1.892542
C	-0.62801	-1.88037	1.262683
C	1.35318	-2.91277	0.297984
C	-0.22817	-2.36704	2.534367
C	1.773451	-3.31691	1.595053
C	0.951969	-3.02245	2.706297
H	-0.87339	-2.17059	3.386202
H	1.266766	-3.34915	3.69355
C	2.138872	-3.31722	-0.80986
C	2.982184	-4.03387	1.745075
C	3.736705	-4.37432	0.656261
C	3.296378	-4.02724	-0.63613
H	3.293699	-4.31675	2.747204
H	4.663463	-4.92499	0.781804
H	3.879412	-4.32792	-1.50118
C	-1.93827	-1.2252	1.229761
C	-2.38904	-0.09698	0.644463
C	-3.82616	0.243516	0.613018
C	-4.26559	1.568644	0.667568
C	-4.79016	-0.76168	0.498571
C	-5.61156	1.877262	0.647721
H	-3.53973	2.37303	0.75017
C	-6.1377	-0.45997	0.463163
H	-4.47572	-1.79618	0.38841
C	-6.57268	0.866683	0.543717
C	-1.54494	0.866068	0.01251
N	-0.95241	1.723592	-0.48522
H	1.810129	-3.06343	-1.81268

H	2.573343	-0.19139	-2.31054
H	-2.69347	-1.76719	1.801753
C	0.166117	-2.13186	0.15054
H	-1.82103	-3.66135	-0.32629
N	-7.94023	1.178066	0.503533
N	7.982855	1.292975	0.163441
H	-5.92931	2.912837	0.715372
H	-6.86688	-1.25578	0.348193
H	6.013179	2.520961	1.47137
H	6.86279	-0.58038	-1.36463
C	8.932359	0.243793	0.102495
C	10.08656	0.385656	-0.66703
C	8.730417	-0.93552	0.819351
C	11.02338	-0.63505	-0.71283
H	10.24323	1.302562	-1.22781
C	9.663615	-1.95821	0.754932
H	7.836309	-1.04366	1.42652
C	10.81614	-1.81316	-0.00693
H	11.91777	-0.51219	-1.31592
H	9.49469	-2.87102	1.317972
H	11.5485	-2.61276	-0.0493
C	8.434167	2.623994	0.339342
C	7.903559	3.658603	-0.43081
C	9.424504	2.91067	1.278353
C	8.350155	4.958653	-0.25319
H	7.139	3.435632	-1.16927
C	9.877866	4.210807	1.438072
H	9.83598	2.105968	1.880728
C	9.341317	5.241935	0.677929
H	7.928823	5.754987	-0.85904
H	10.64916	4.420772	2.17272
H	9.693801	6.259756	0.809434
C	-8.39187	2.362737	-0.1303
C	-7.89418	2.734166	-1.37884
C	-9.35589	3.159479	0.48579
C	-8.34695	3.891863	-1.9921
H	-7.14987	2.109321	-1.86381
C	-9.81642	4.306466	-0.1416
H	-9.74163	2.870769	1.459253
C	-9.31226	4.681503	-1.3803
H	-7.95152	4.170836	-2.96394
H	-10.5675	4.918542	0.34827
H	-9.66988	5.58329	-1.86649
C	-8.89655	0.294643	1.062837
C	-8.69952	-0.25222	2.330471

C	-10.0533	-0.02514	0.353112
C	-9.64092	-1.11449	2.870457
H	-7.80357	0.004434	2.888183
C	-10.9983	-0.87475	0.90703
H	-10.2056	0.399178	-0.63508
C	-10.796	-1.42739	2.16499
H	-9.47649	-1.53338	3.858495
H	-11.8951	-1.11561	0.344482
H	-11.5347	-2.09657	2.594069

Table S6: Cartesian coordinates of **BNPD5** compound.

Atoms	X-axis	Y-axis	Z-axis
C	3.082798	1.779927	-3.77038
C	3.792764	2.826354	-3.24851
C	3.304276	3.508081	-2.1166
C	2.146369	3.104915	-1.50733
H	3.430943	1.261335	-4.65972
H	4.72005	3.145412	-3.71333
H	3.849379	4.362325	-1.72709
C	-0.10485	-0.01534	-3.19645
C	1.086731	0.332925	-3.7536
C	1.871244	1.357761	-3.17728
C	1.405584	2.000965	-1.99829
C	0.216895	1.534044	-1.35654
C	-0.55033	0.553321	-1.97321
H	-0.72483	-0.77429	-3.66558
H	1.434908	-0.14751	-4.66363
C	-1.87078	0.100658	-1.53023
C	-2.39381	-0.22544	-0.33094
C	-3.84032	-0.49699	-0.16765
C	-4.29887	-1.43049	0.764264
C	-4.78017	0.200132	-0.93086
C	-5.6506	-1.68924	0.903152
H	-3.58666	-1.97798	1.374564
C	-6.13379	-0.04389	-0.78498
H	-4.44995	0.97513	-1.61635
C	-6.57664	-0.99565	0.129328
C	-8.67149	-1.17863	1.476425
C	-8.84451	-1.56743	-0.74189
C	-10.0262	-1.46619	1.215843
C	-10.1362	-1.71612	-0.19896
C	-1.63472	-0.36286	0.869635
N	-1.11185	-0.54412	1.883362
C	0.629502	1.95242	1.070346

C	-1.36464	2.902256	0.051452
C	0.236118	2.520691	2.309293
C	-1.78121	3.386037	1.322278
C	-0.94965	3.174834	2.444914
H	0.889203	2.388998	3.167443
H	-1.26106	3.564784	3.409934
C	-2.15908	3.226481	-1.07625
C	-2.99741	4.097591	1.432547
C	-3.76128	4.357713	0.3283
C	-3.32339	3.933817	-0.94179
H	-3.30725	4.441763	2.41573
H	-4.69376	4.904675	0.423345
H	-3.91367	4.172982	-1.82098
C	1.943824	1.304181	1.070223
C	2.390974	0.134696	0.57279
C	3.832244	-0.20387	0.576602
C	4.267626	-1.50993	0.807929
C	4.785212	0.784954	0.324116
C	5.616993	-1.81228	0.83186
H	3.540923	-2.29493	0.996264
C	6.135583	0.483348	0.326514
H	4.4621	1.79314	0.079012
C	6.559666	-0.81619	0.590061
C	8.91564	-0.4586	1.332875
C	8.55049	-2.14271	-0.12825
C	10.16572	-1.05588	1.076194
C	9.933041	-2.12895	0.143398
C	1.552907	-0.87701	0.015048
N	0.965043	-1.77111	-0.41966
H	-1.83222	2.912676	-2.06269
H	-2.57144	0.003478	-2.36118
H	2.703527	1.89109	1.589813
C	-0.1729	2.122043	-0.05075
H	1.780735	3.645108	-0.63985
N	7.937234	-1.12446	0.598339
N	-7.95862	-1.24244	0.281775
C	10.76967	-3.05014	-0.48145
C	7.995842	-3.04622	-1.02838
C	8.785124	0.596924	2.228917
C	11.30631	-0.57302	1.712388
C	-8.59515	-1.7761	-2.09395
C	-11.199	-2.06112	-1.02991
C	-8.2295	-0.85393	2.754264
C	-10.9493	-1.44661	2.258138
C	-9.16732	-0.84062	3.774269

C	-10.513	-1.13732	3.534803
C	-9.66889	-2.11888	-2.8999
C	-10.9596	-2.25677	-2.37918
C	10.22093	-3.96188	-1.36648
C	8.84888	-3.95256	-1.63727
C	11.1848	0.487344	2.593679
C	9.934452	1.059186	2.849698
H	8.44013	-4.66977	-2.3423
H	11.83689	-3.04689	-0.27759
H	9.85931	1.883073	3.552655
H	-9.50115	-2.28653	-3.9593
H	-12.1986	-2.17827	-0.62043
H	-11.996	-1.66658	2.066913
H	-8.84769	-0.5916	4.781442
H	5.948441	-2.82206	1.051837
H	6.934285	-3.04209	-1.253
H	7.818666	1.041821	2.442293
H	6.868932	1.249363	0.095195
H	-5.99577	-2.44086	1.606053
H	-7.18938	-0.61337	2.948979
H	-6.85853	0.525396	-1.35854
H	-7.5957	-1.68158	-2.50583
H	12.06401	0.877175	3.096353
H	12.27438	-1.02721	1.519761
H	10.85831	-4.68799	-1.86063
H	-11.2191	-1.11891	4.358686
H	-11.777	-2.52476	-3.04077

Table S7: Machine learning algorithms' performance based on R^2, RMSE, and MAE along both test and training datasets.

Model	Train			Test		
	R^2	RMSE	MAE	R^2	RMSE	MAE
**CatBoost**	0.999	0.040	0.028	0.890	0.454	0.267
**GradientBoosting**	0.999	0.038	0.027	0.888	0.457	0.257
**XGBoost**	0.993	0.109	0.083	0.861	0.510	0.297
**HistGradientBoosting**	1.000	0.022	0.007	0.835	0.555	0.308
**RandomForest**	0.961	0.256	0.164	0.789	0.628	0.362

Table S8: Calculated cross-validation parameters of 5-fold CV.

Model	CV R^2	CV RMSE	CV MAE
Random Forest	0.7913 ± 0.0329	0.5924 ± 0.0525	0.3595 ± 0.0163

Model	CV R^2	CV RMSE	CV MAE
Gradient Boosting	0.8584 ± 0.0312	0.4866 ± 0.0574	0.2852 ± 0.0211
HistGradientBoosting	0.8403 ± 0.0245	0.5190 ± 0.0546	0.3014 ± 0.0225
XGBoost	0.8626 ± 0.0231	0.4806 ± 0.0473	0.2853 ± 0.0173
CatBoost	**0.8674 ± 0.0220**	**0.4727 ± 0.0500**	**0.2813 ± 0.0156**

Table S9: Default and optimal hyperparameters of CatBoost algorithm.

Hyperparameter	Optimal Value (Used in Study)	Default Value (CatBoost)
**iterations**	500	500
**learning_rate**	0.05	~0.01
**depth**	6	6
**loss_function**	RMSE	RMSE (for regression)
**random_seed**	42	0 (or None)
**verbose**	0	1 (default output enabled)

Table S10: Actual versus predicted energy gaps of investigated chromophores.

Compounds	Predicted E_{gap}	Actual E_{gap}
**BPNR**	3.236	4.05
**BNPD1**	3.034	3.192
**BNPD2**	3.025	3.425
**BNPD3**	3.017	2.682
**BNPD4**	3.229	3.504
**BNPD5**	3.131	3.769

Table S11: Description and types of the top ten descriptors.

Descriptor	Type	Description
**BCUT2D_LOGPLOW**	Topological / Physicochemical	Represents the lower eigenvalue of a matrix weighted by atomic logP contributions; reflects distribution of hydrophobicity in the molecule.
**PEOE_VSA9**	Electronic / Surface Area	Sum of van der Waals surface areas of atoms with partial charges in the range 0.05–0.10, indicating moderately positive charge regions.
**EState_VSA10**	Electronic / Topological	Combines electrotopological state values with surface area, representing regions with specific electronic environments and spatial contribution.
**MinAbsEStateIndex**	Electronic	Minimum absolute electrotopological state value, reflecting the least electronically influential atom in the molecule.
**PEOE_VSA7**	Electronic / Surface	Surface area of atoms with near-neutral partial charges

Descriptor	Type	Description
	Area	(−0.05 to 0.00), indicating weakly charged regions.
**SMR_VSA10**	Physicochemical	Surface area of atoms with high molar refractivity contribution (MR ≥ 4.0), related to polarizability and steric effects.
**NumRotatableBonds**	Structural / Flexibility	Number of rotatable bonds in a molecule, indicating molecular flexibility and conformational freedom.
**BalabanJ**	Topological	A connectivity index that measures molecular branching and complexity based on graph theory.
**MolWt**	Constitutional	Molecular weight of the compound, representing overall size of the molecule
**PEOE_VSA11**	Electronic / Surface Area	Surface area of atoms with higher positive partial charges (0.15–0.20), indicating strongly electron-deficient regions

Table S12: Calculated energies (***E***) and energy gap (**Δ*E***) of **HOMO-1, LUMO+1, HOMO-2** and **LUMO+2** for designed compounds.

Compounds	HOMO-1	LUMO+1	Δ*E*	HOMO-2	LUMO+2	Δ*E*
**BNPR**	-6.459	-2.124	4.335	-6.668	-1.522	5.146
**BNPD1**	-5.913	-2.055	3.858	-6.340	-1.508	4.832
**BNPD2**	-5.850	-2.024	3.826	-6.325	-1.502	4.823
**BNPD3**	-5.724	-2.068	3.656	-6.207	-1.467	4.740
**BNPD4**	-5.691	-2.059	3.632	-6.302	-1.458	4.844
**BNPD5**	-6.085	-2.201	3.884	-6.376	-1.560	4.816

E= energy, $\Delta E(eV) = E_{LUMO} - E_{HOMO}$; **HOMO**= highest occupied molecular orbital; **LUMO**= lowest unoccupied molecular orbital; **MO**= molecular orbital; Units in ***eV***

Table S13: Global reactivity descriptors of **BNPR** and designed chromophores (**BNPD1-BNPD5)***,* all units are *eV*, softness is in eV^{-1}

Compounds	*IP*	*EA*	*X*	*η*	*μ*	*ω*	*σ*	ΔN_{max}
**BNPR**	6.264	2.214	4.239	2.025	-4.239	4.436	0.246	2.093
**BNPD1**	5.428	2.236	3.832	1.596	-3.832	4.600	0.313	2.401
**BNPD2**	5.638	2.213	3.926	1.713	-3.926	4.499	0.292	2.292
**BNPD3**	4.896	2.214	3.555	1.341	-3.555	4.712	0.372	2.651
**BNPD4**	5.671	2.167	3.919	1.752	-3.919	4.383	0.285	2.236
**BNPD5**	6.076	2.307	4.191	1.884	-4.191	4.661	0.265	2.224

Table S14: Wavelength, excitation energy and oscillator strength of investigated compound **BNPR** in the gaseous phase

No	DFT *λ (nm)*	*E(eV)*	f_{os}	MO contributions
1	383.164	3.236	0.038	H→L (92%), H-2→L (3%)
2	376.166	3.296	0.256	H→L+1 (95%),
3	354.686	3.496	0.558	H-1→L (91%), H-3→L (2%)
4	344.095	3.603	0.229	H-1→L+1 (90%), H-4→L+1 (2%), H-2→L (2%), H→L (3%)

5	328.313	3.776	0.021	H-3→L (15%), H-2→L (31%), H-2→L+1 (12%), H→L+2 (25%), H-5→L+1 (3%), H-4→L (2%)
6	325.811	3.805	0.017	H-2→L (49%), H-2→L+1 (14%), H→L+2 (13%), H-5→L (3%), H-5→L+1 (2%), H-3→L (5%), H-3→L+1 (5%), H-1→L+1 (2%)

MO=molecular orbital, H=HOMO, L=LUMO, f_{os}= oscillator strength, wavelength= λ (*nm*)

Table S15: Wavelength, excitation energy and oscillator strength of investigated compound **BNPD1** in the gaseous phase

No	DFT ***λ (nm)***	***E(eV)***	f_{os}	MO contributions
1	485.033	2.556	0.000	H→L (94%),
2	423.357	2.929	0.000	H→L+1 (97%),
3	403.700	3.071	0.009	H-2→L (16%), H-1→L (79%), H-3→L (3%)
4	387.160	3.202	0.575	H-1→L+1 (85%), H-2→L (3%), H-2→L+1 (7%)
5	370.080	3.350	0.075	H-2→L (75%), H-1→L (15%), H-2→L+1 (3%), H-1→L+1 (3%)
6	362.951	3.416	0.000	H→L+2 (94%), H→L+4 (3%)

MO=molecular orbital, H=HOMO, L=LUMO, f_{os}= oscillator strength, wavelength= λ (*nm*)

Table S16: Wavelength, excitation energy and oscillator strength of investigated compound **BNPD2** in the gaseous phase

NO	DFT ***λ (nm)***	***E(eV)***	f_{os}	MO contributions
1	440.238	2.816	0.001	H→L (94%), H→L+1 (2%)
2	407.642	3.042	0.016	H-2→L (11%), H-1→L (85%), H-3→L (3%)
3	391.574	3.166	0.614	H-1→L+1 (85%), H-2→L (3%), H-2→L+1 (4%), H→L+1 (3%)
4	388.264	3.193	0.021	H→L+1 (93%), H-1→L+1 (3%), H→L (2%)
5	371.066	3.341	0.080	H-2→L (82%), H-1→L (10%), H-1→L+1 (3%)
6	357.550	3.468	0.031	H-2→L+1 (88%), H-1→L+1 (5%)

MO=molecular orbital, H=HOMO, L=LUMO, f_{os}= oscillator strength, wavelength= λ (*nm*)

Table S17: Wavelength, excitation energy and oscillator strength of investigated compound **BNPD3** in the gaseous phase

No	DFT ***λ (nm)***	***E(eV)***	f_{os}	MO contributions
1	587.102	2.112	0.000	H→L (93%), H→L+1 (4%)
2	513.413	2.415	0.000	H→L+1 (95%), H→L (4%)
3	420.656	2.947	0.000	H→L+2 (97%),
4	415.943	2.981	0.044	H-2→L (14%), H-1→L (82%),

5	406.826	3.048	0.000	H→L+6 (75%), H→L+7 (14%), H→L+14 (9%)
6	401.243	3.090	0.558	H-1→L+1 (86%), H-2→L (2%), H-2→L+1 (8%)

MO=molecular orbital, H=HOMO, L=LUMO, f_{os}= oscillator strength, wavelength= λ (*nm*)

Table S18: Wavelength, excitation energy and oscillator strength of investigated compound **BNPD4** in the gaseous phase

No	DFT *λ (nm)*	*E(eV)*	f_{os}	MO contributions
1	418.032	2.966	0.730	H-1→L (15%), H→L (61%), H→L+1 (16%), H-2→L (2%)
2	412.003	3.009	0.343	H-1→L (46%), H→L (28%), H→L+1 (11%), H-2→L (4%), H-1→L+1 (7%)
3	400.078	3.099	0.065	H-1→L (32%), H-1→L+1 (18%), H→L+1 (38%), H-2→L+1 (6%)
4	395.358	3.136	0.055	H-1→L+1 (65%), H→L+1 (23%), H-3→L+1 (3%), H-2→L (5%)
5	367.502	3.374	0.012	H-2→L (84%), H-1→L+1 (5%), H→L (6%)
6	356.984	3.473	0.017	H-2→L+1 (85%), H→L+1 (9%)

MO=molecular orbital, H=HOMO, L=LUMO, f_{os}= oscillator strength, wavelength= λ (*nm*)

Table S19: Wavelength, excitation energy and oscillator strength of investigated compound **BNPD5** in the gaseous phase

No	DFT *λ (nm)*	*E(eV)*	f_{os}	MO contributions
1	396.902	3.124	0.345	H-4→L (23%), H→L (66%), H→L+1 (6%)
2	390.674	3.174	0.360	H-4→L (20%), H-1→L (43%), H-1→L+1 (12%), H→L+1 (14%), H-5→L (2%), H-4→L+1 (6%)
3	382.986	3.237	0.065	H-4→L (19%), H-4→L+1 (13%), H-1→L+1 (40%), H→L (16%), H-1→L (4%), H→L+1 (2%)
4	378.635	3.275	0.015	H-4→L+1 (28%), H-1→L (20%), H-1→L+1 (14%), H→L+1 (24%), H-5→L+1 (3%), H-4→L (8%)
5	366.351	3.384	0.024	H-4→L (27%), H-1→L (25%), H-1→L+1 (28%), H→L (11%), H-4→L+1 (3%)
6	358.854	3.455	0.007	H-4→L+1 (45%), H→L+1 (47%), H→L (2%)

MO=molecular orbital, H=HOMO, L=LUMO, f_{os}= oscillator strength, wavelength= λ (*nm*)

Table S20: Wavelength, excitation energy and oscillator strength of investigated compound **BNPR** in the solvent phase

No	DFT λ (nm)	E(eV)	f_{os}	MO contributions
1	392.442	3.159	0.066	H→L (95%),
2	384.471	3.225	0.337	H→L+1 (96%),
3	360.534	3.439	0.695	H-1→L (90%), H-3→L (2%), H-2→L+1 (3%)
4	349.862	3.544	0.308	H-1→L+1 (87%), H-4→L+1 (2%), H-2→L (7%)
5	331.970	3.735	0.042	H-3→L (17%), H-2→L (51%), H→L+2 (10%), H-5→L (3%), H-3→L+1 (3%), H-2→L+1 (6%), H-1→L+1 (3%)
6	329.693	3.761	0.025	H-3→L (20%), H-2→L (25%), H-2→L+1 (19%), H→L+2 (15%), H-5→L+1 (3%), H-4→L (3%), H-3→L+1 (6%), H-1→L+1 (3%)

MO=molecular orbital, H=HOMO, L=LUMO, f_{os}= oscillator strength, wavelength= λ (*nm*)

Table S21: Wavelength, excitation energy and oscillator strength of investigated compound **BNPD1** in the solvent phase

No	DFT λ (nm)	E(eV)	f_{os}	MO contributions
1	472.879	2.622	0.000	H→L (94%), H→L+1 (2%)
2	415.859	2.981	0.001	H→L+1 (96%), H→L (3%)
3	408.461	3.035	0.033	H-2→L (22%), H-1→L (73%), H-3→L (2%)
4	397.067	3.123	0.657	H-1→L+1 (83%), H-2→L (5%), H-2→L+1 (7%)
5	377.517	3.284	0.096	H-2→L (68%), H-1→L (21%), H-3→L (2%), H-2→L+1 (4%), H-1→L+1 (3%)
6	365.487	3.392	0.059	H-2→L+1 (84%), H-3→L+1 (3%), H-1→L+1 (8%)

MO=molecular orbital, H=HOMO, L=LUMO, f_{os}= oscillator strength, wavelength= λ (*nm*)

Table S22: Wavelength, excitation energy and oscillator strength of investigated compound **BNPD2** in the solvent phase

NO	DFT λ (nm)	E(eV)	f_{os}	MO contributions
1	436.011	2.844	0.001	H→L (93%), H→L+1 (3%)
2	413.529	2.998	0.066	H-2→L (13%), H-1→L (81%), H-3→L (2%), H-1→L+1 (2%)
3	401.894	3.085	0.692	H-1→L+1 (85%), H-2→L (6%), H-2→L+1 (4%)
4	385.823	3.214	0.002	H→L+1 (95%), H→L (3%)
5	379.331	3.269	0.100	H-2→L (77%), H-1→L (14%), H-1→L+1 (4%)
6	365.078	3.396	0.044	H-2→L+1 (89%), H-1→L+1 (5%)

MO=molecular orbital, H=HOMO, L=LUMO, f_{os}= oscillator strength, wavelength= λ (*nm*)

Table S23: Wavelength, excitation energy and oscillator strength of investigated compound **BNPD3** in the solvent phase

No	DFT *λ (nm)*	*E(eV)*	f_{os}	MO contributions
1	575.867	2.153	0.000	H→L (91%), H→L+1 (6%)
2	507.841	2.441	0.000	H→L+1 (93%), H→L (6%)
3	422.131	2.937	0.188	H-2→L (15%), H-1→L (72%), H-1→L+1 (10%),
4	411.020	3.017	0.471	H-2→L+1 (10%), H-1→L+1 (73%), H-3→L (2%), H-2→L (7%), H-1→L (5%)
5	408.138	3.038	0.000	H→L+2 (95%), H→L+4 (3%)
6	403.463	3.073	0.001	H→L+4 (70%), H→L+6 (16%), H→L+2 (4%), H→L+14 (9%)

MO=molecular orbital, H=HOMO, L=LUMO, f_{os}= oscillator strength, wavelength= λ (*nm*)

Table S24: Wavelength, excitation energy and oscillator strength of investigated compound **BNPD4** in the solvent phase

No	DFT *λ (nm)*	*E(eV)*	f_{os}	MO contributions
1	434.179	2.856	0.858	H-1→L (27%), H→L (47%), H→L+1 (20%),
2	427.650	2.899	0.405	H-1→L (30%), H-1→L+1 (15%), H→L (38%), H→L+1 (11%), H-2→L (3%)
3	410.068	3.024	0.062	H-1→L (37%), H-1→L+1 (24%), H→L+1 (29%), H-2→L (2%), H-2→L+1 (4%)
4	405.641	3.057	0.032	H-1→L+1 (53%), H→L+1 (28%), H-3→L+1 (2%), H-2→L (6%), H-2→L+1 (3%), H→L (5%)
5	377.460	3.285	0.017	H-2→L (85%), H-1→L+1 (4%), H→L (7%)
6	365.886	3.389	0.022	H-2→L+1 (87%), H→L+1 (9%)

MO=molecular orbital, H=HOMO, L=LUMO, f_{os}= oscillator strength, wavelength= λ (*nm*)

Table S25: Wavelength, excitation energy and oscillator strength of investigated compound **BNPD5** in the solvent phase

No	DFT *λ (nm)*	*E(eV)*	f_{os}	MO contributions
1	401.789	3.086	0.218	H-2→L (48%), H→L (46%), H→L+1 (2%)
2	393.826	3.148	0.634	H-2→L+1 (22%), H-1→L (26%), H→L+1 (33%), H-2→L (8%), H-1→L+1 (6%)
3	384.853	3.222	0.063	H-2→L (12%), H-2→L+1 (24%), H-1→L (26%), H-1→L+1 (10%), H→L (18%), H-5→L (4%), H→L+1 (3%)
4	380.110	3.262	0.102	H-2→L (10%), H-2→L+1 (15%), H-1→L (16%), H-

				1→L+1 (42%), H-5→L+1 (3%), H→L (7%), H→L+1 (2%)
5	364.584	3.401	0.035	H-2→L (19%), H-1→L (20%), H-1→L+1 (31%), H→L (22%), H-5→L+1 (2%), H-2→L+1 (3%)
6	358.160	3.462	0.012	H-2→L+1 (31%), H→L+1 (53%), H-5→L (2%), H-5→L+1 (2%), H-1→L (2%), H-1→L+1 (2%), H→L (3%)

MO=molecular orbital, H=HOMO, L=LUMO, f_{os}= oscillator strength, wavelength= λ (nm)

Table S26: Calculated E_b **BNPR** and **BNPD1-BNPD5** compounds.

Compounds	$E_{H\text{–}L}$	E_{opt}	E_b
**BNPR**	4.050	3.439	0.611
**BNPD1**	3.192	3.123	0.069
**BNPD2**	3.425	3.085	0.340
**BNPD3**	2.682	3.017	0.335
**BNPD4**	3.504	2.856	0.648
**BNPD5**	3.769	3.148	0.621

Units in *eV*

Table S27: Percentages of Acceptor and Donor for LUMOs and HOMOs in **BNPR** and **BNPD1-BNPD5** chromophores.

**Compounds**	**LUMO**		**HOMO**	
	**Acceptor**	**Donor**	**Acceptor**	**Donor**
**BNPR**	14.6	85.4	15.2	84.8
**BNPD1**	14.3	85.7	100.0	0.0
**BNPD2**	13.8	86.2	99.9	0.1
**BNPD3**	12.5	87.5	100.0	0.0
**BNPD4**	11.6	88.4	89.0	11.0
**BNPD5**	12.8	87.2	90.8	9.2

Table S28. Hole-electron analysis indices for $S_0 \rightarrow S_1$ excitation (with highest f_{osc}) of entitled compounds.

Compounds	Excitation	λ (nm)	E (eV)	*D* (Å)	E_{Coul} (eV)	S_r	*H* (Å)	*t* (Å)	HDI	EDI
**BNPR**	$S_0 \rightarrow S_1$	360.534	3.159	1.147	3.27	0.67794	3.995	-1.297	4.50	4.85
**BNPD1**	$S_0 \rightarrow S_1$	397.067	2.622	8.187	1.67	0.07207	2.947	6.380	9.11	6.50
**BNPD2**	$S_0 \rightarrow S_1$	401.894	2.844	8.054	1.70	0.08047	8.054	6.207	8.99	6.46
**BNPD3**	$S_0 \rightarrow S_1$	411.020	2.153	8.531	1.64	0.06479	2.991	6.701	8.54	6.44
**BNPD4**	$S_0 \rightarrow S_1$	434.179	2.856	5.517	2.43	0.44478	4.318	2.016	7.22	5.59
**BNPD5**	$S_0 \rightarrow S_1$	393.826	3.086	0.887	3.09	0.62609	4.443	-2.067	4.19	5.57

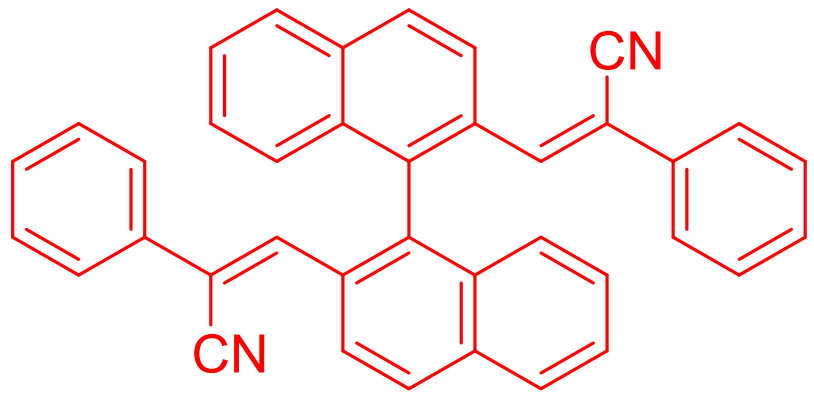

(2Z,2'Z)-3,3'-([1,1'-binaphthalene]-2,2'-diyl)bis(2-phenylacrylonitrile)
BNPR

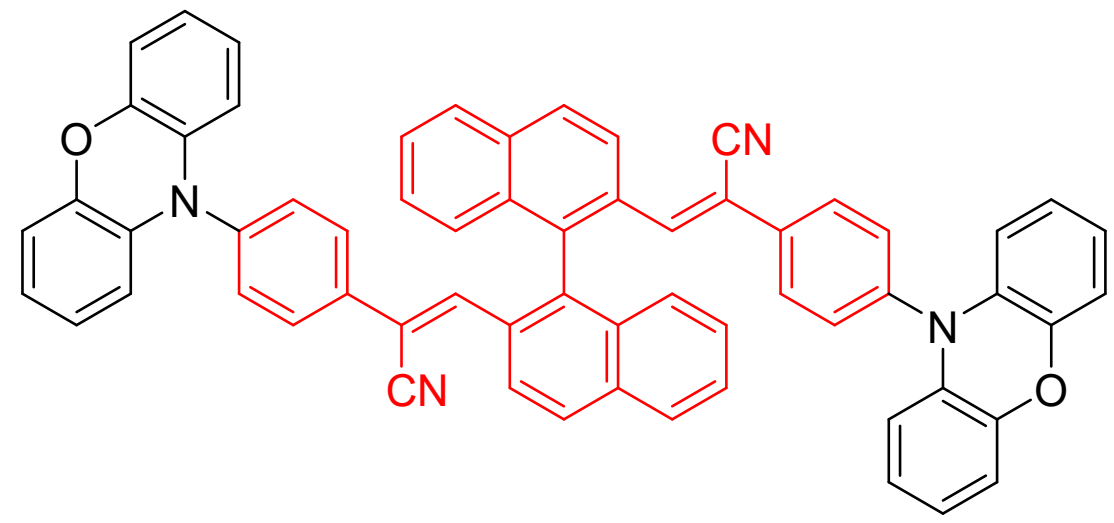

(2Z,2'Z)-3,3'-([1,1'-binaphthalene]-2,2'-diyl)bis(2-(4-(10H-phenoxazin-10-yl)phenyl)acrylonitrile)

BNPD1

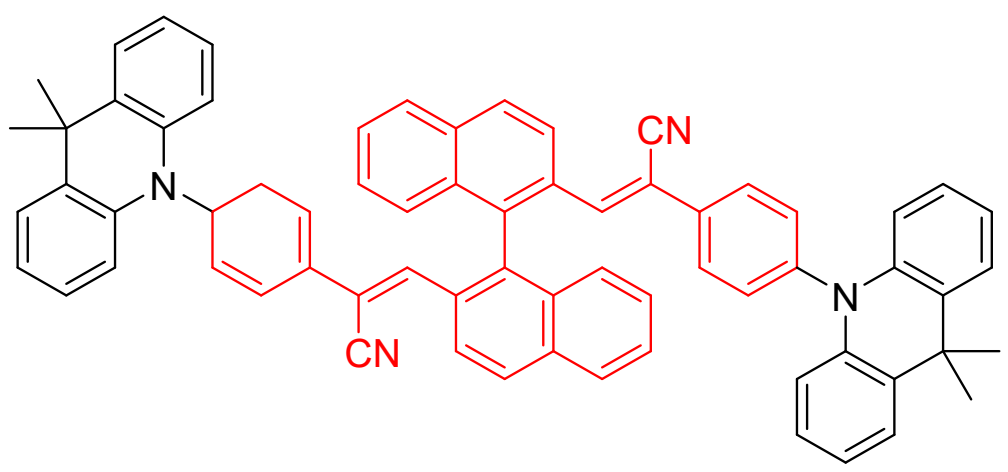

(Z)-3-(2'-((Z)-2-cyano-2-(4-(9,9-dimethylacridin-10(9H)-yl)cyclohexa-1,5-dien-1-yl)vinyl)-[1,1'-binaphthalen]-2-yl)-2-(4-(9,9-dimethylacridin-10(9H)-yl)phenyl)acrylonitrile
BNPD2

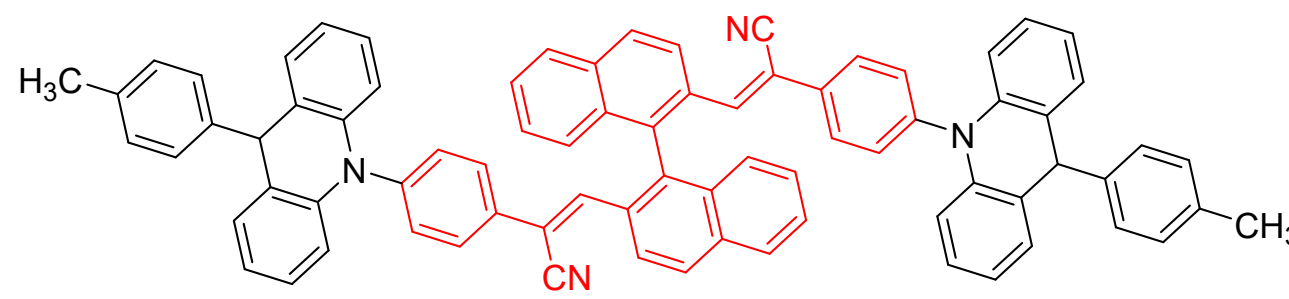

(2Z,2'Z)-3,3'-([1,1'-binaphthalene]-2,2'-diyl)bis(2-(4-(9-(p-tolyl)acridin-10(9H)-yl)phenyl)acrylonitrile)

BNPD3

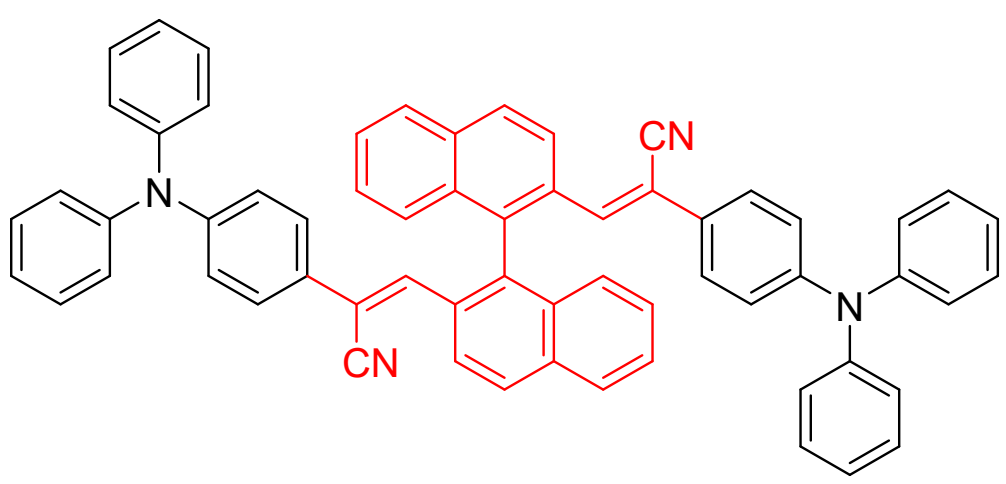

(2Z,2'Z)-3,3'-([1,1'-binaphthalene]-2,2'-diyl)bis(2-(4-(diphenylamino)phenyl)acrylonitrile)
BNPD4

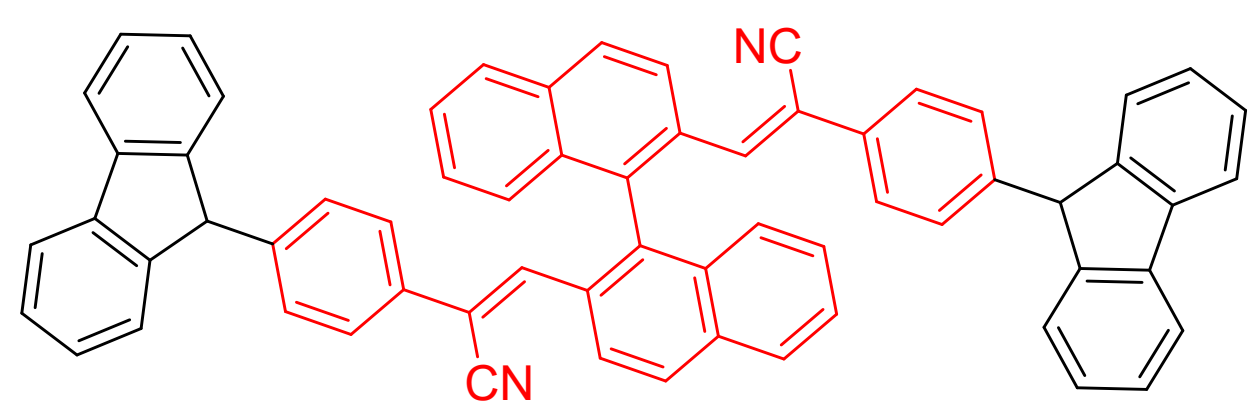

(2Z,2'Z)-3,3'-([1,1'-binaphthalene]-2,2'-diyl)bis(2-(4-(9H-fluoren-9-yl)phenyl)acrylonitrile)

BNPD5

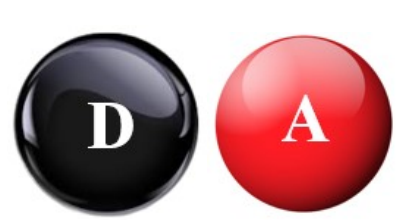

Figure S1: The ChemDraw structural representation along with IUPAC names of the reference (**BNPR**) and derivatives (**BNPD1-BNPD5**) chromophores.

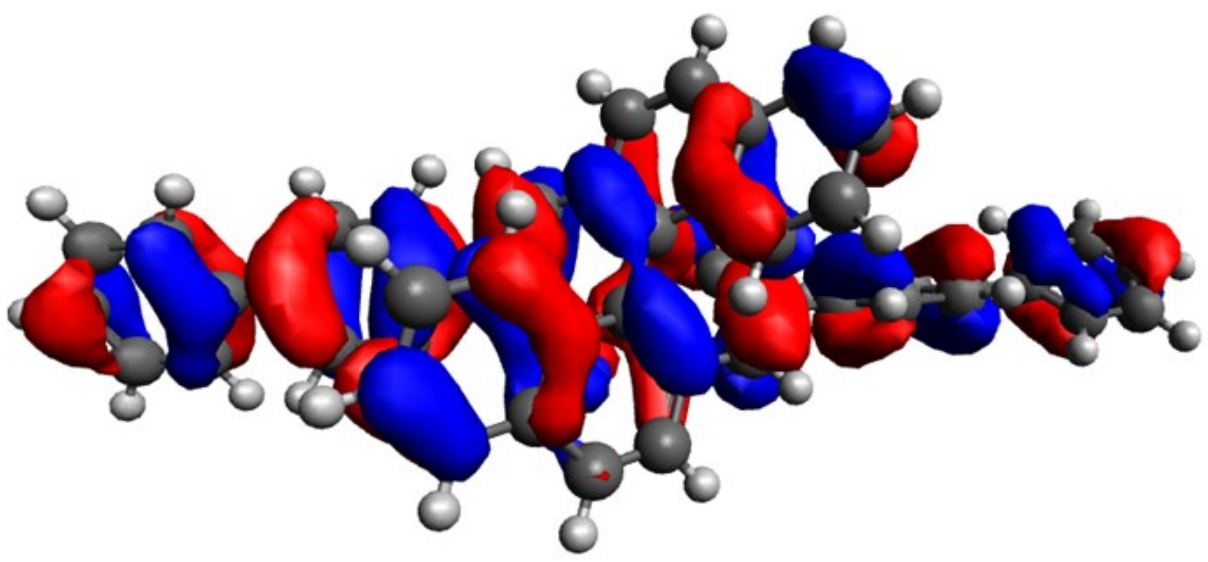

HOMO-1

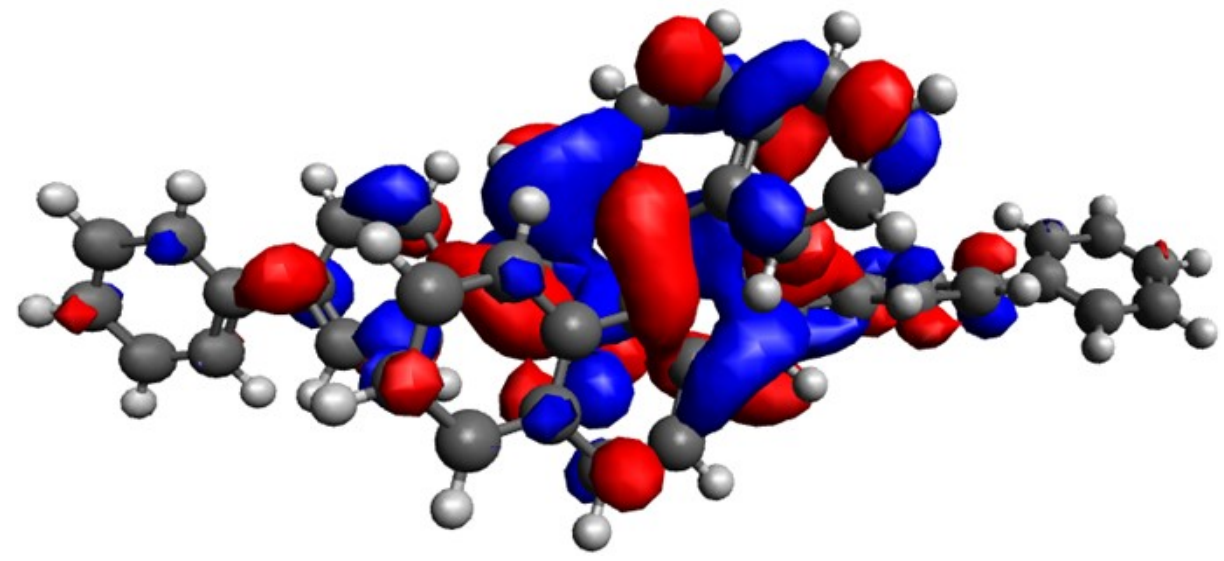

LUMO+1

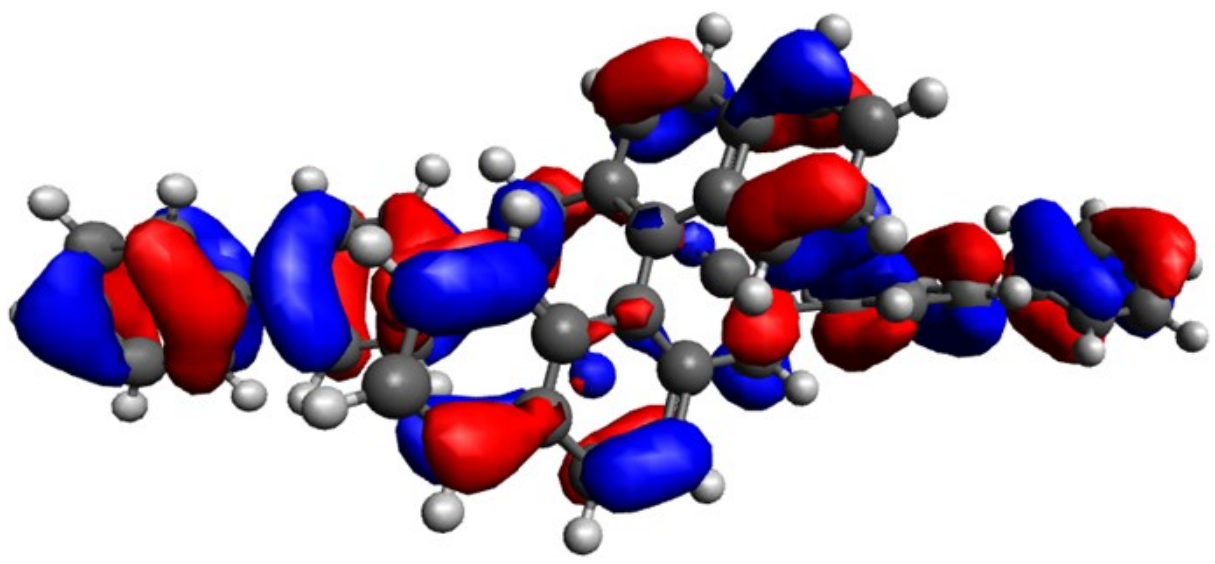

HOMO-2

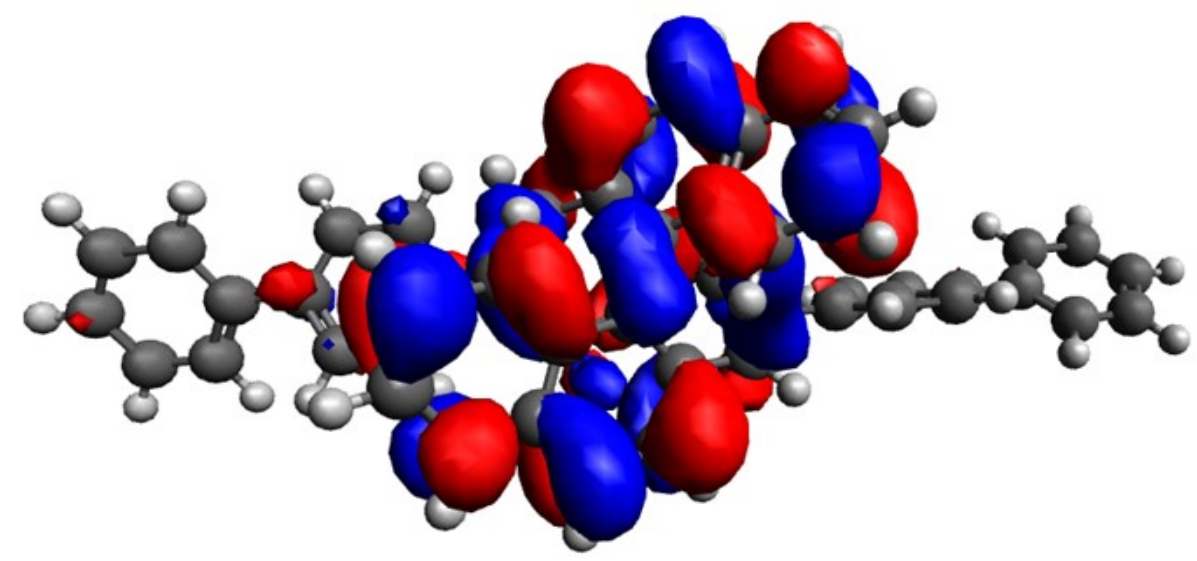

LUMO+2

BNPR

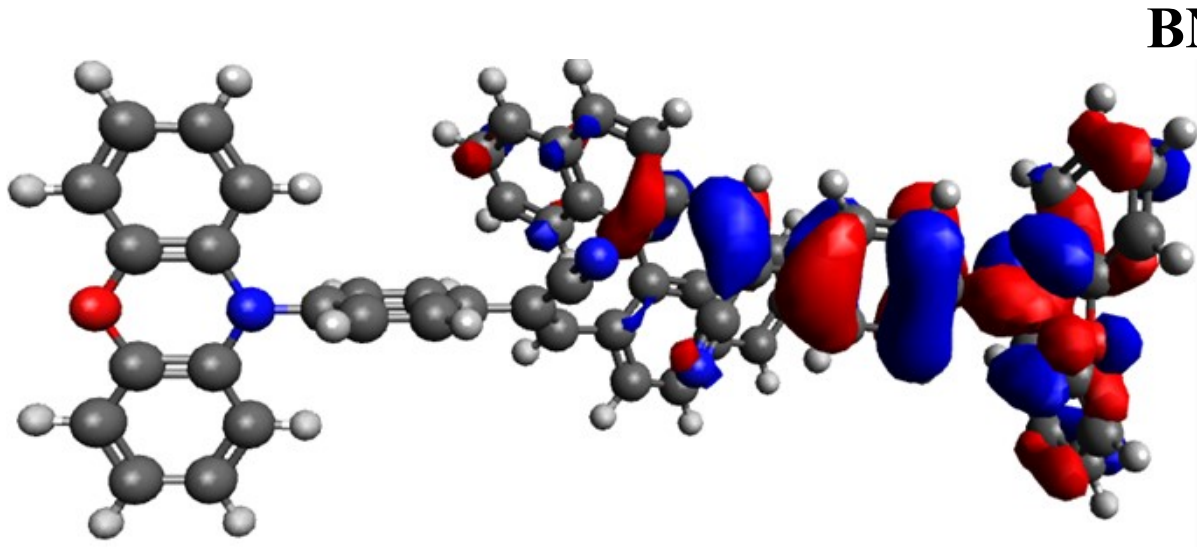

HOMO-1

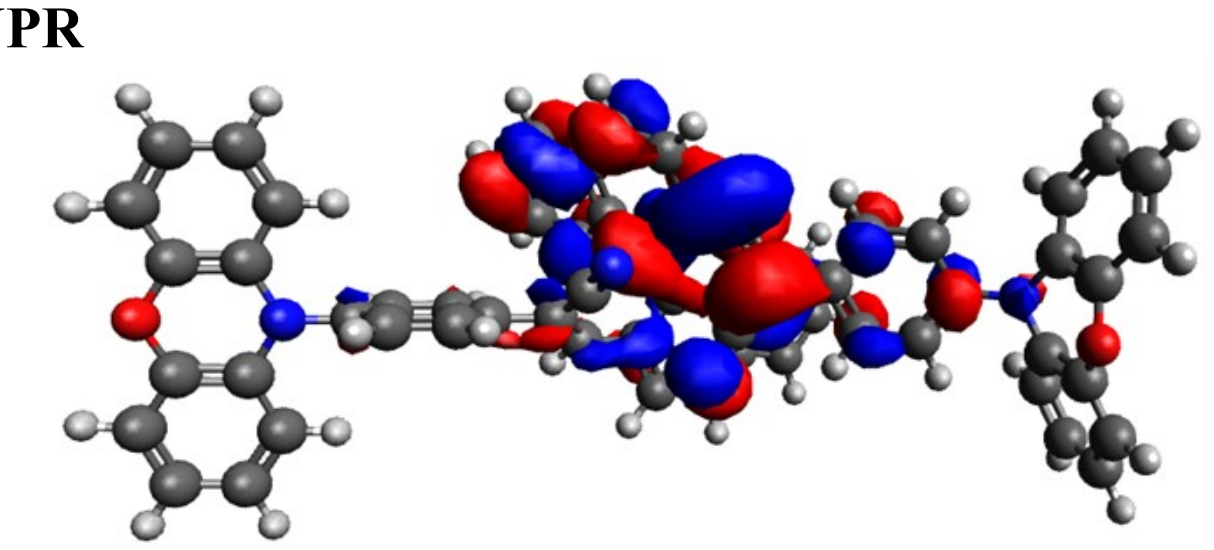

LUMO+1

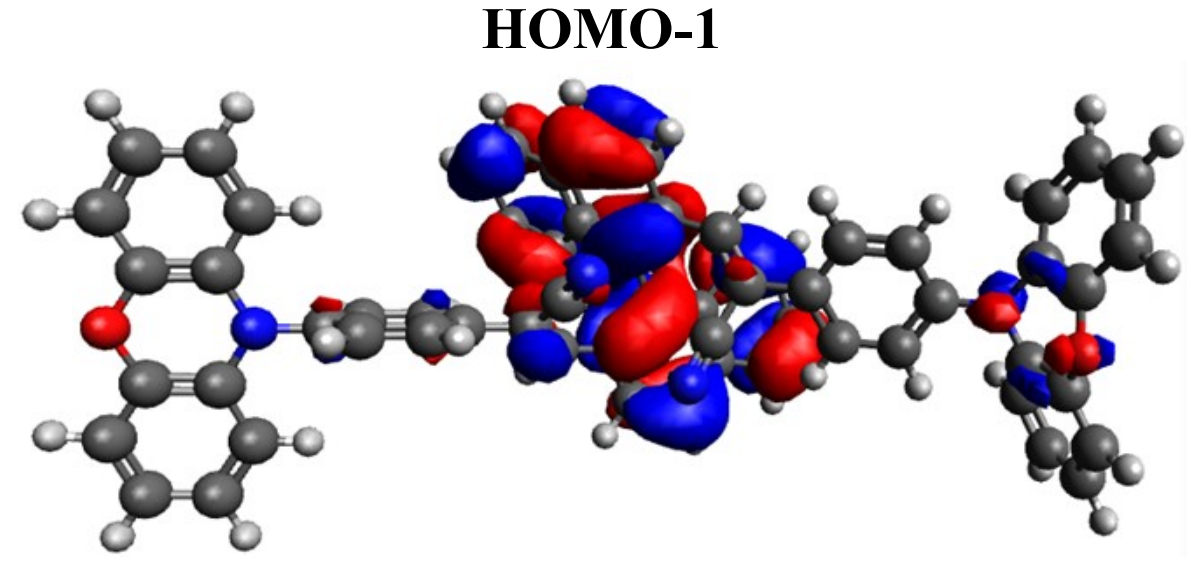

HOMO-2

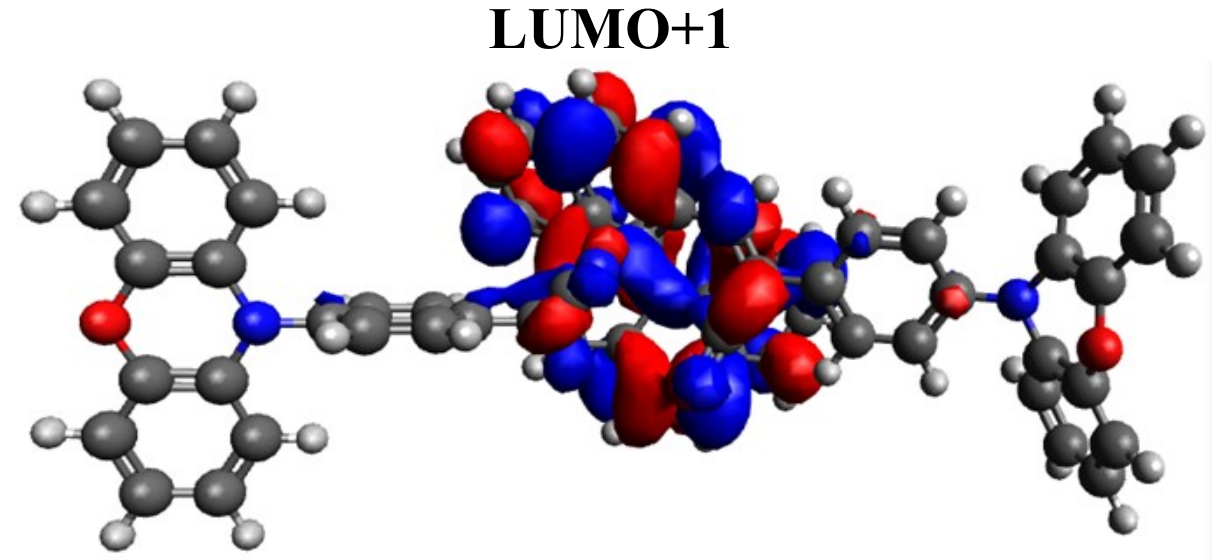

LUMO+2

BNPD1

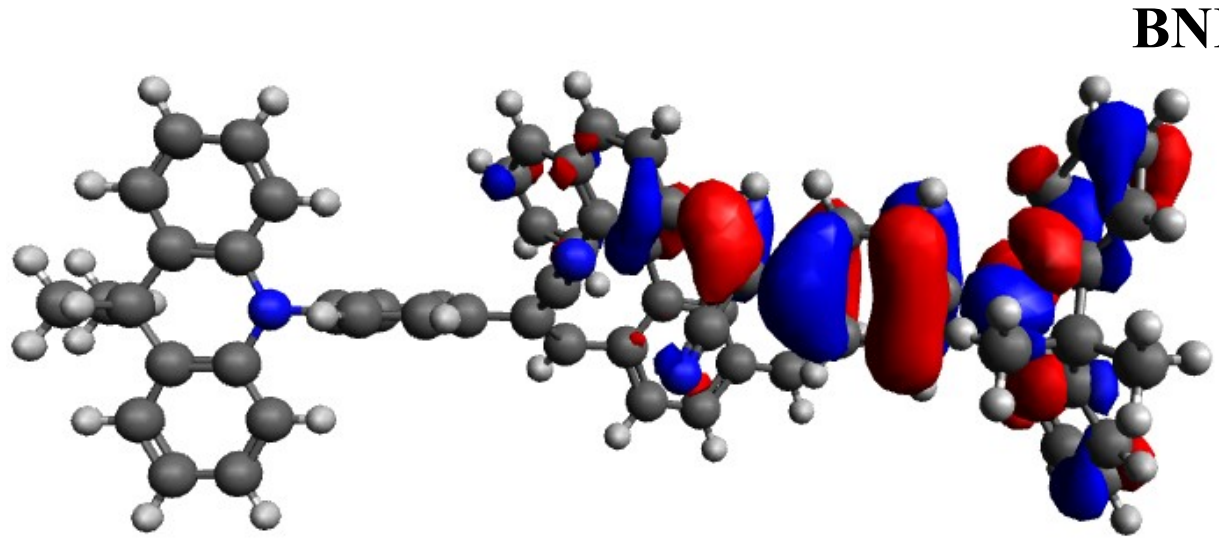

HOMO-1

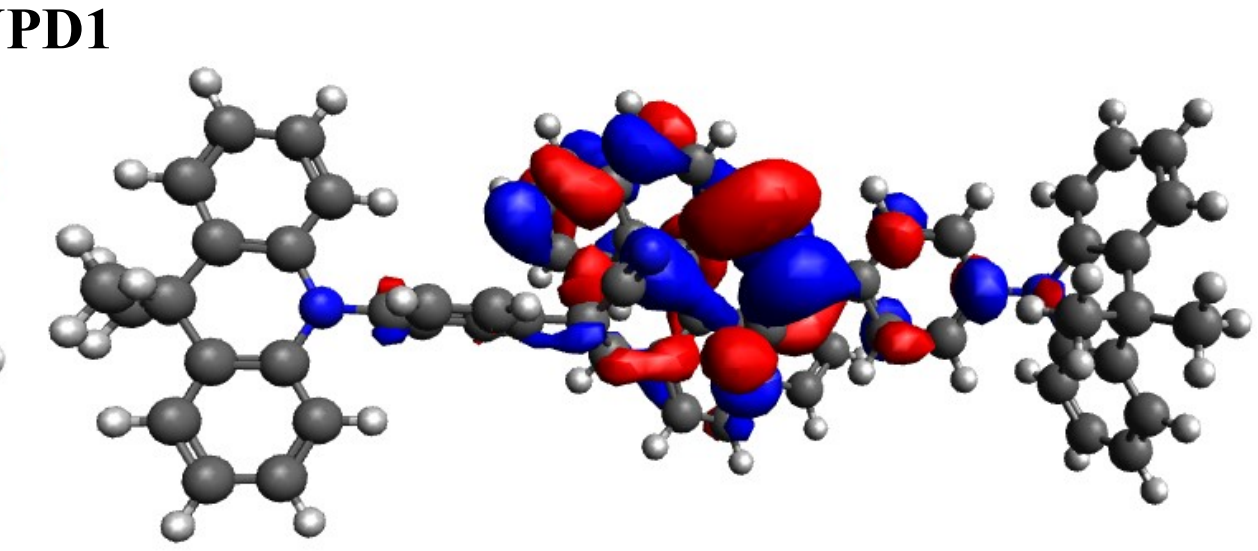

LUMO+1

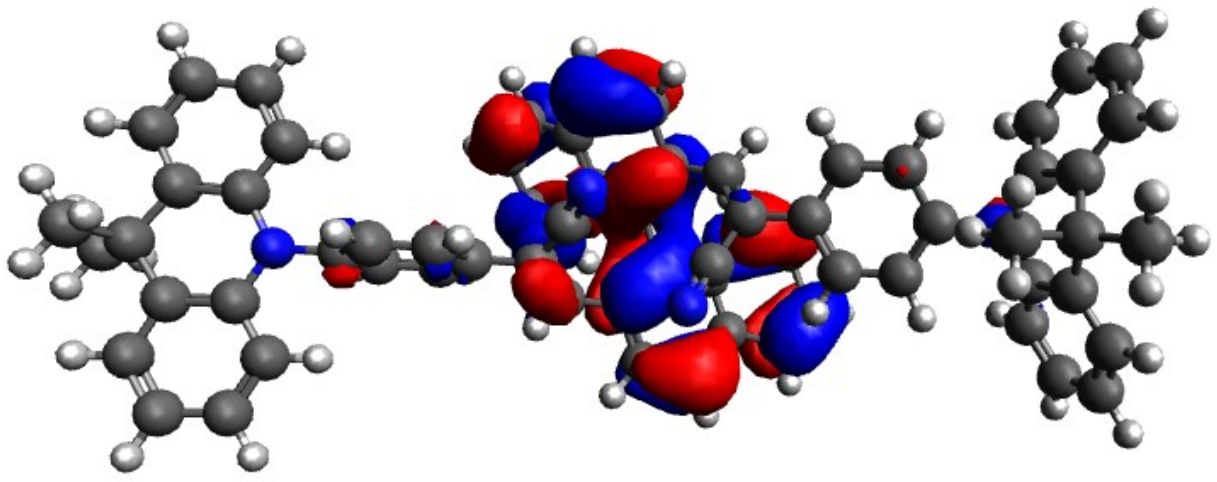

HOMO-2

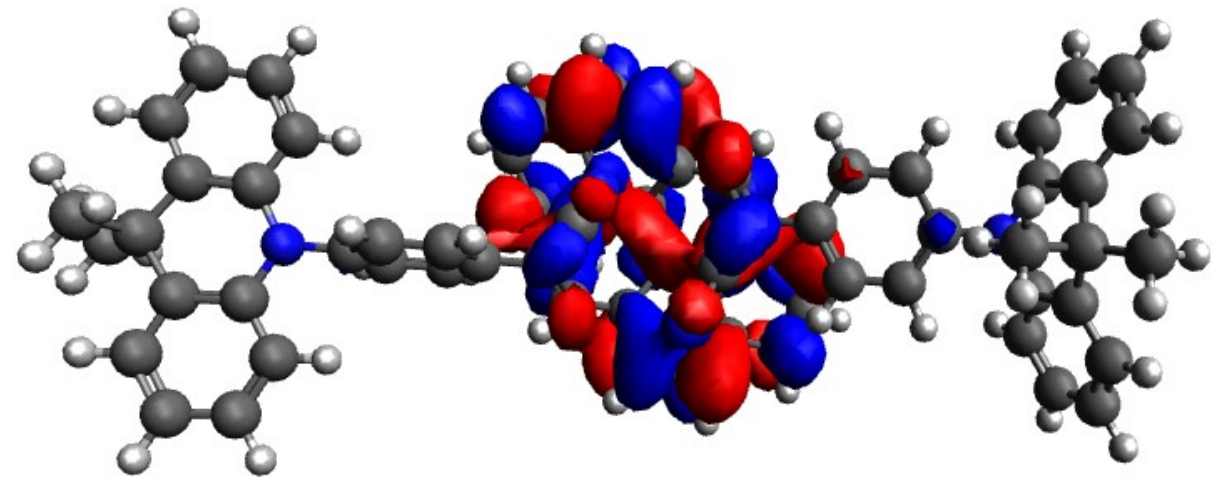

LUMO+2

BNPD2

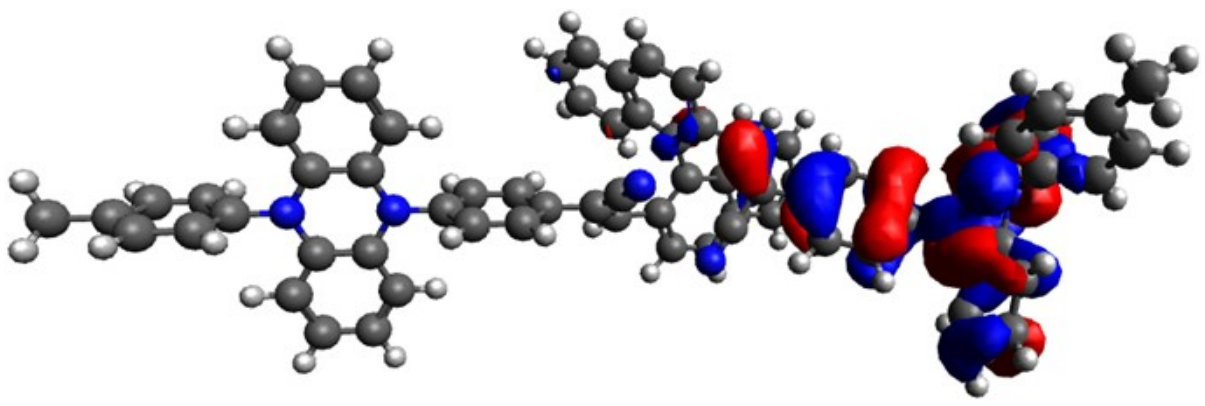

HOMO-1

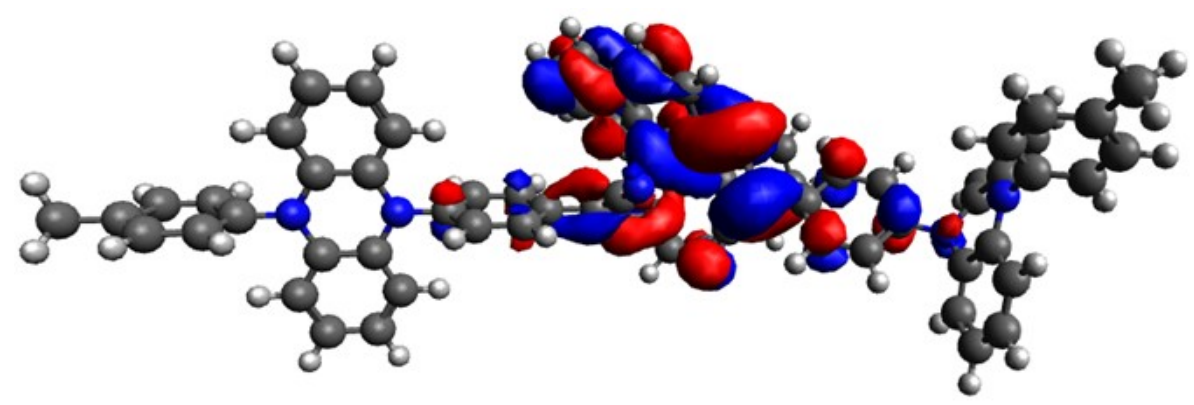

LUMO+1

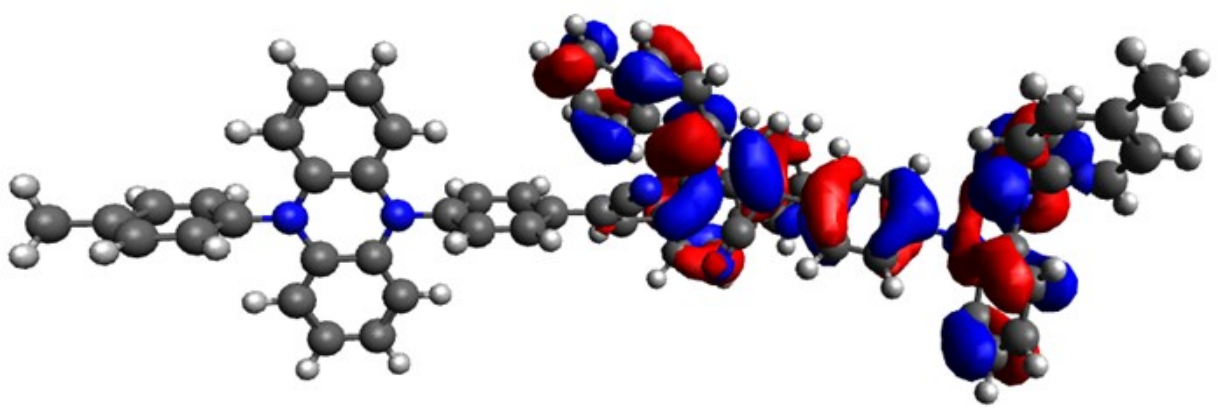

HOMO-2

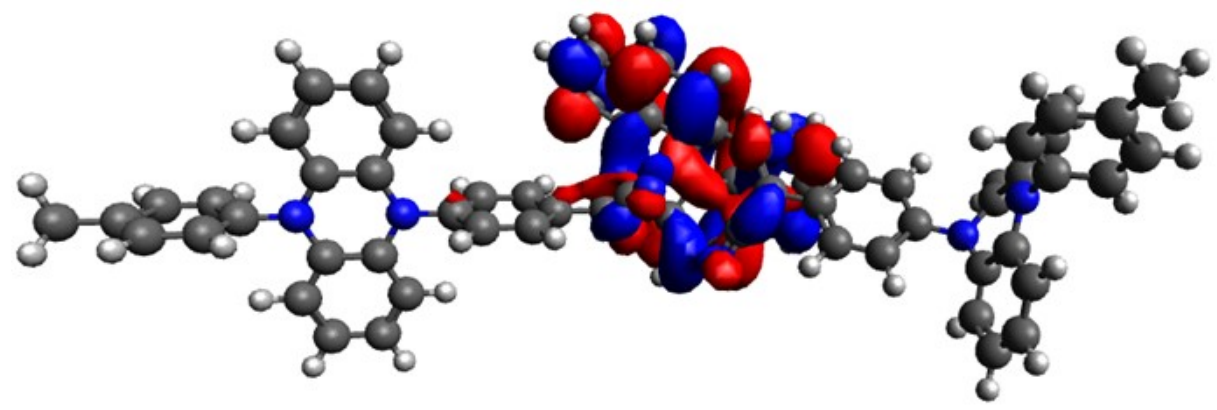

LUMO+2

BNPD3

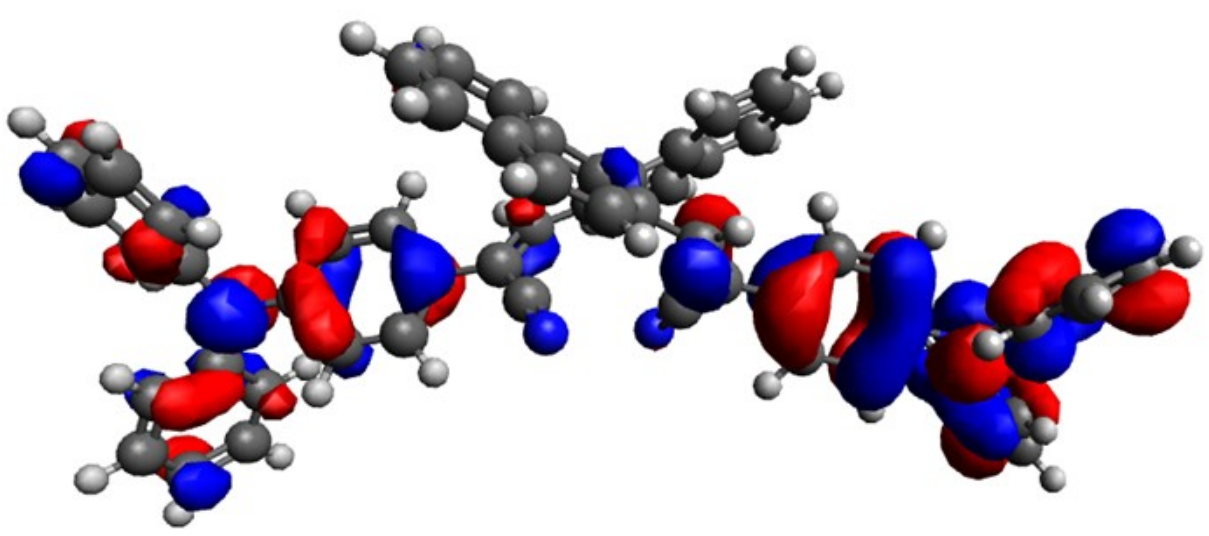

HOMO-1

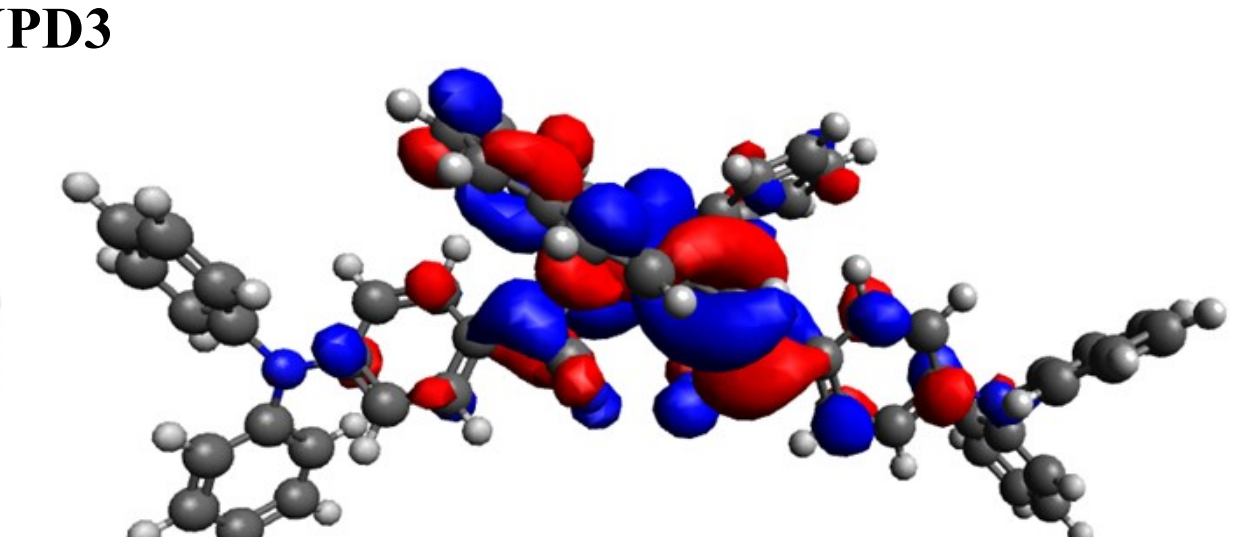

LUMO+1

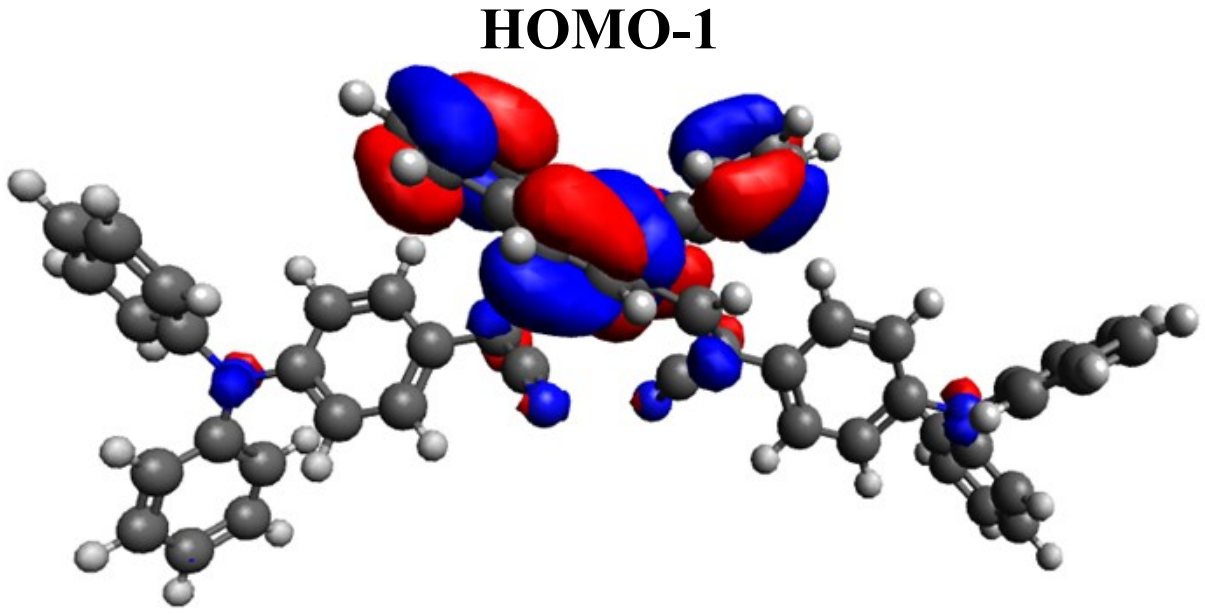

HOMO-2

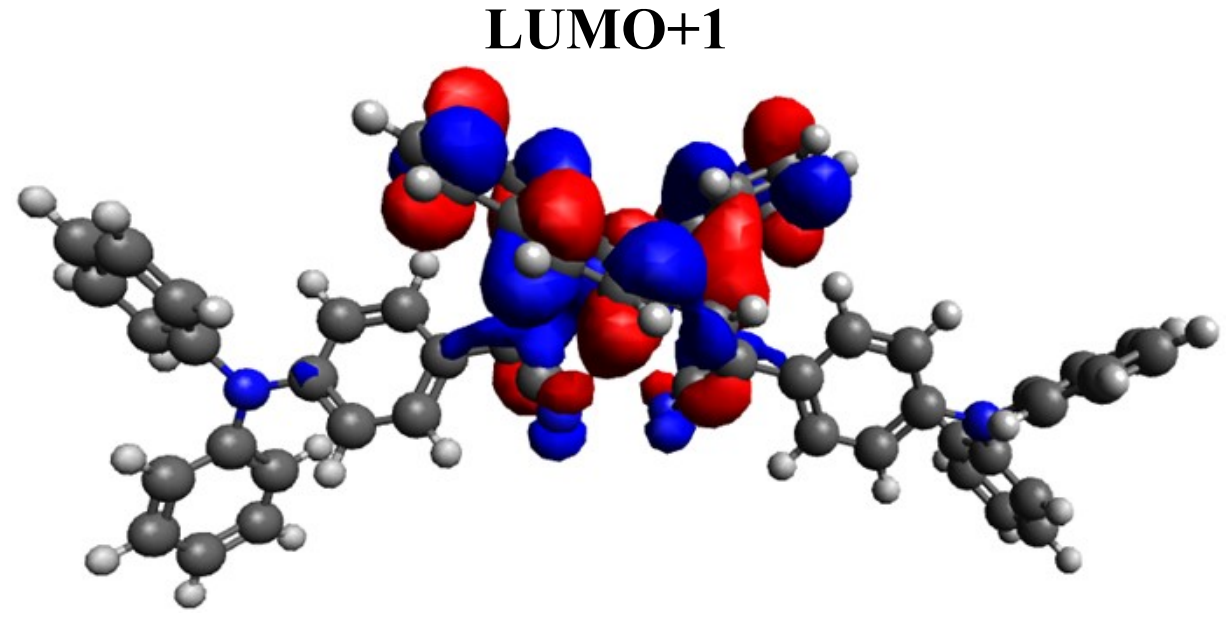

LUMO+2

BNPD4

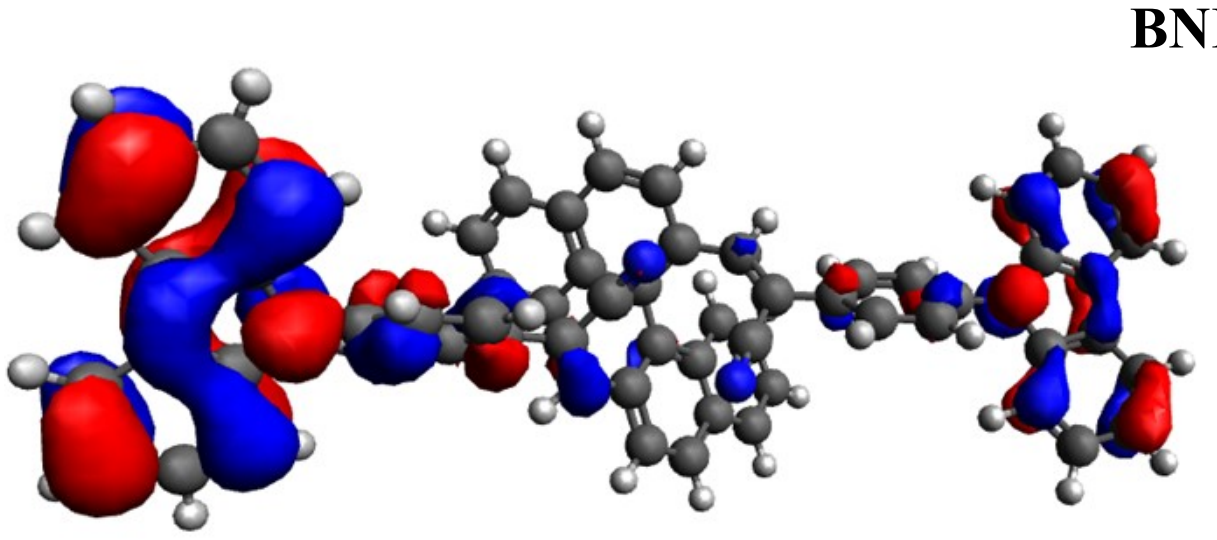

HOMO-1

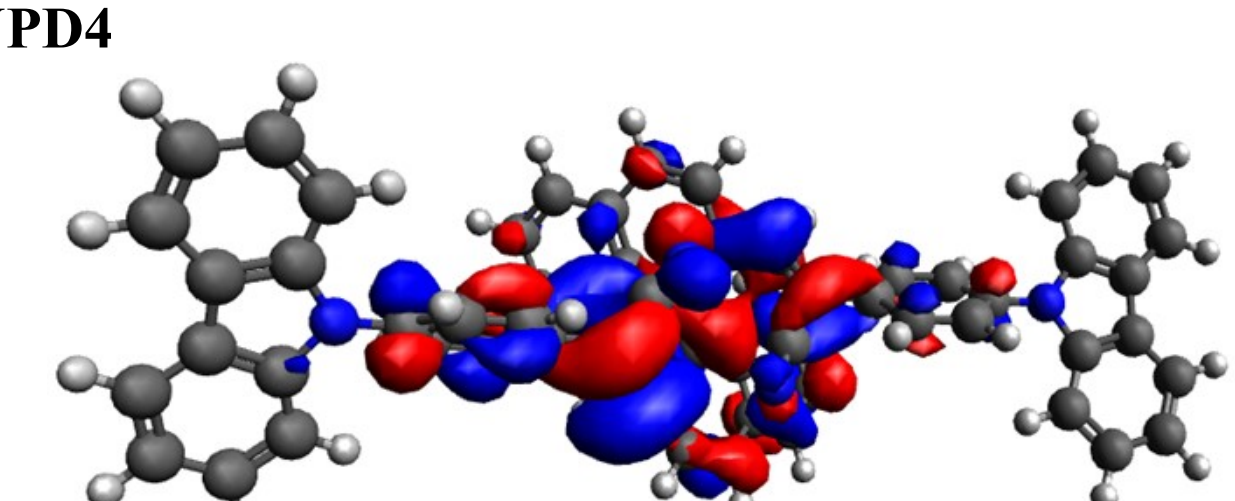

LUMO+1

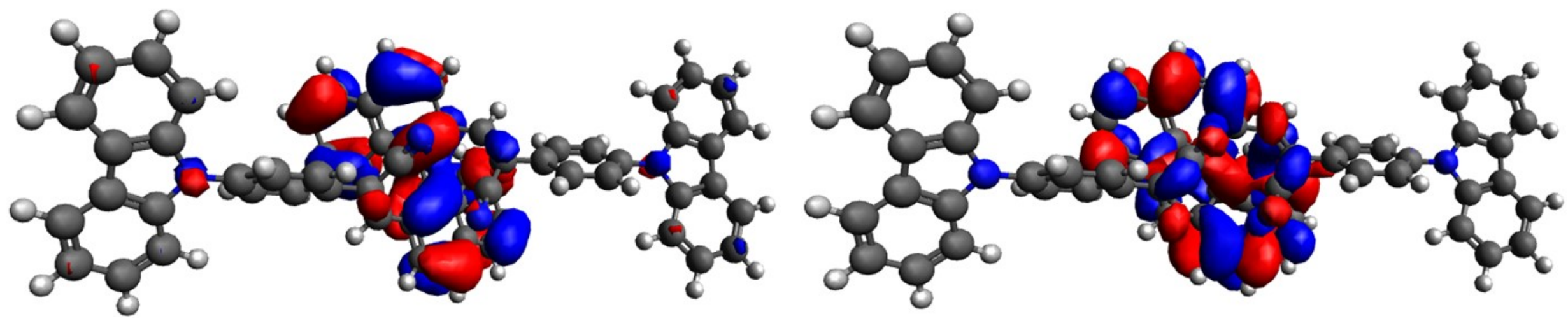

HOMO-2 **LUMO+2**

BNPD5

Figure S2: HOMO-1, LUMO+1, HOMO-2 and LUMO+2 of the investigated chromophores **BNPR** and **PNPD1-BNPD5**.

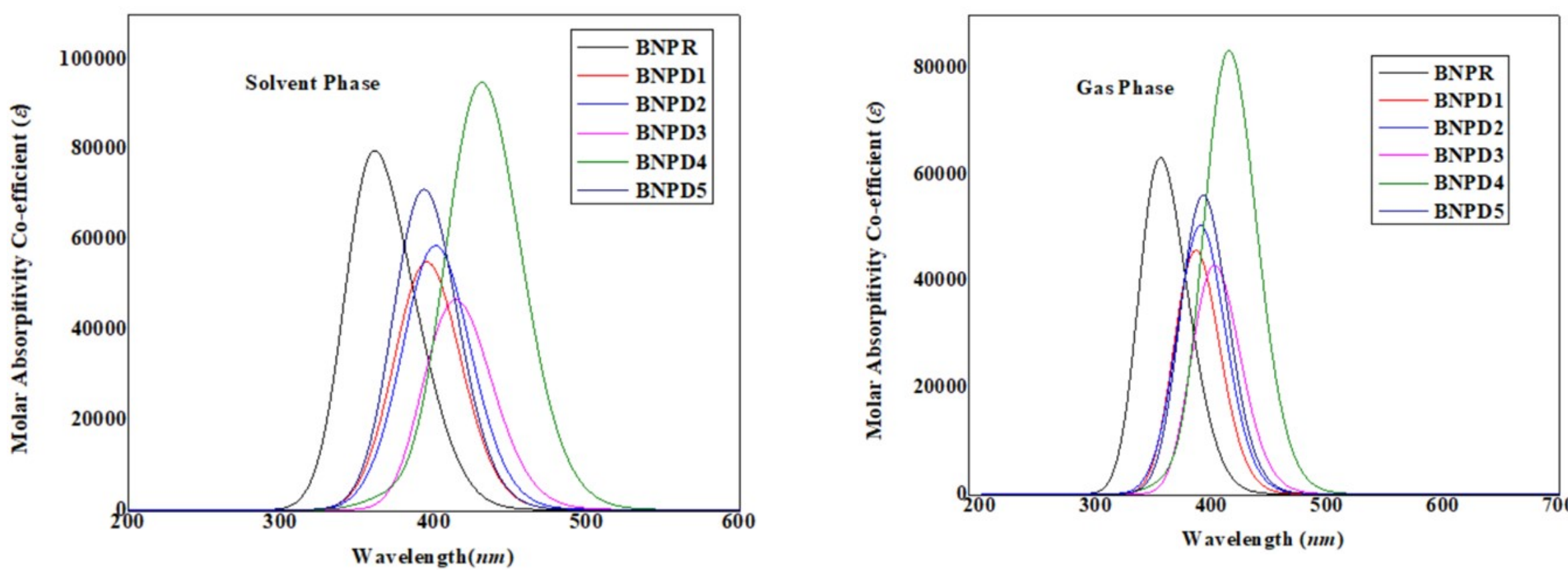

Figure S3: Pictorial representation of UV-Visible graph in solvent and gas phase of **BNPR** and **BNPD1-BNPD5** chromophores.

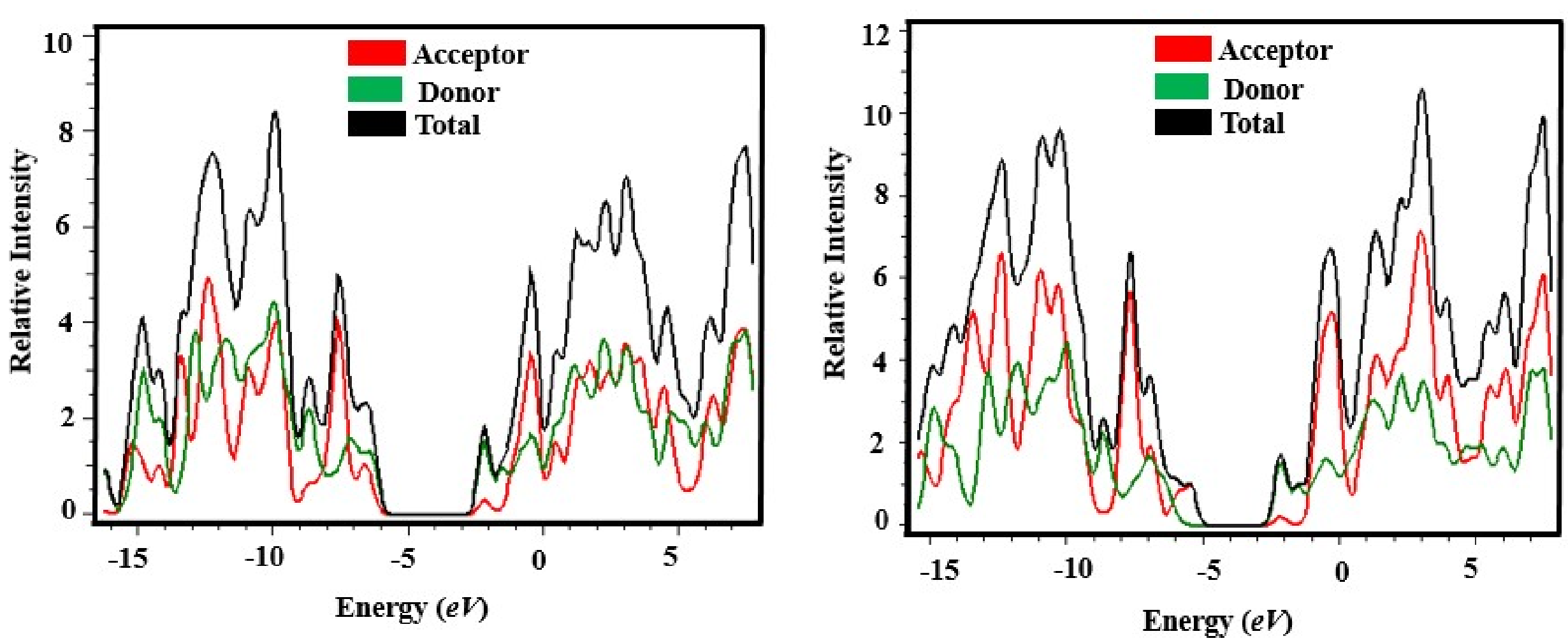

BNPR **BNPD1**

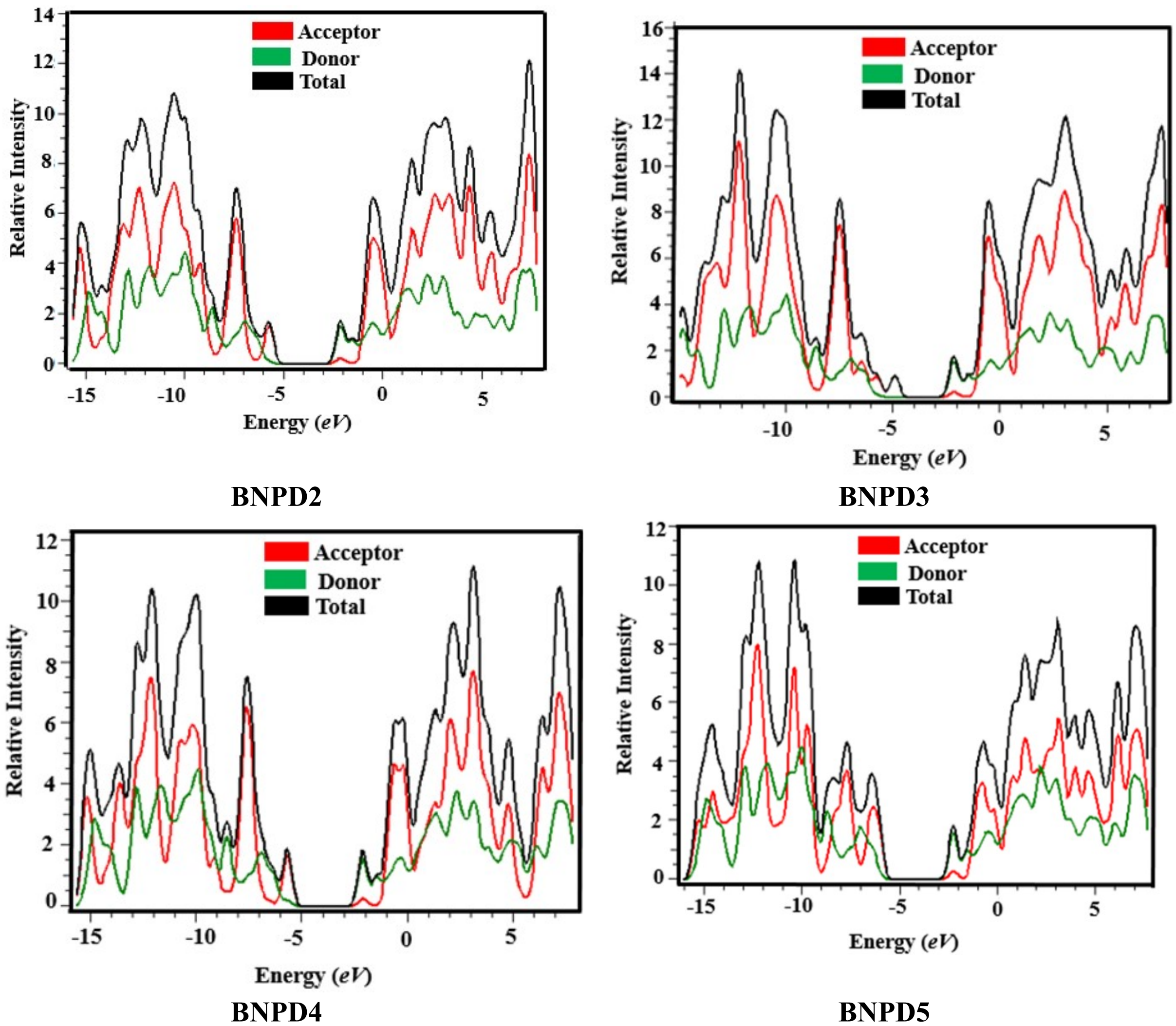

Figure S4: Pictorial representations of DOS fragments of **BNPR** and (**BNPD1-BNPD5)** chromophores.

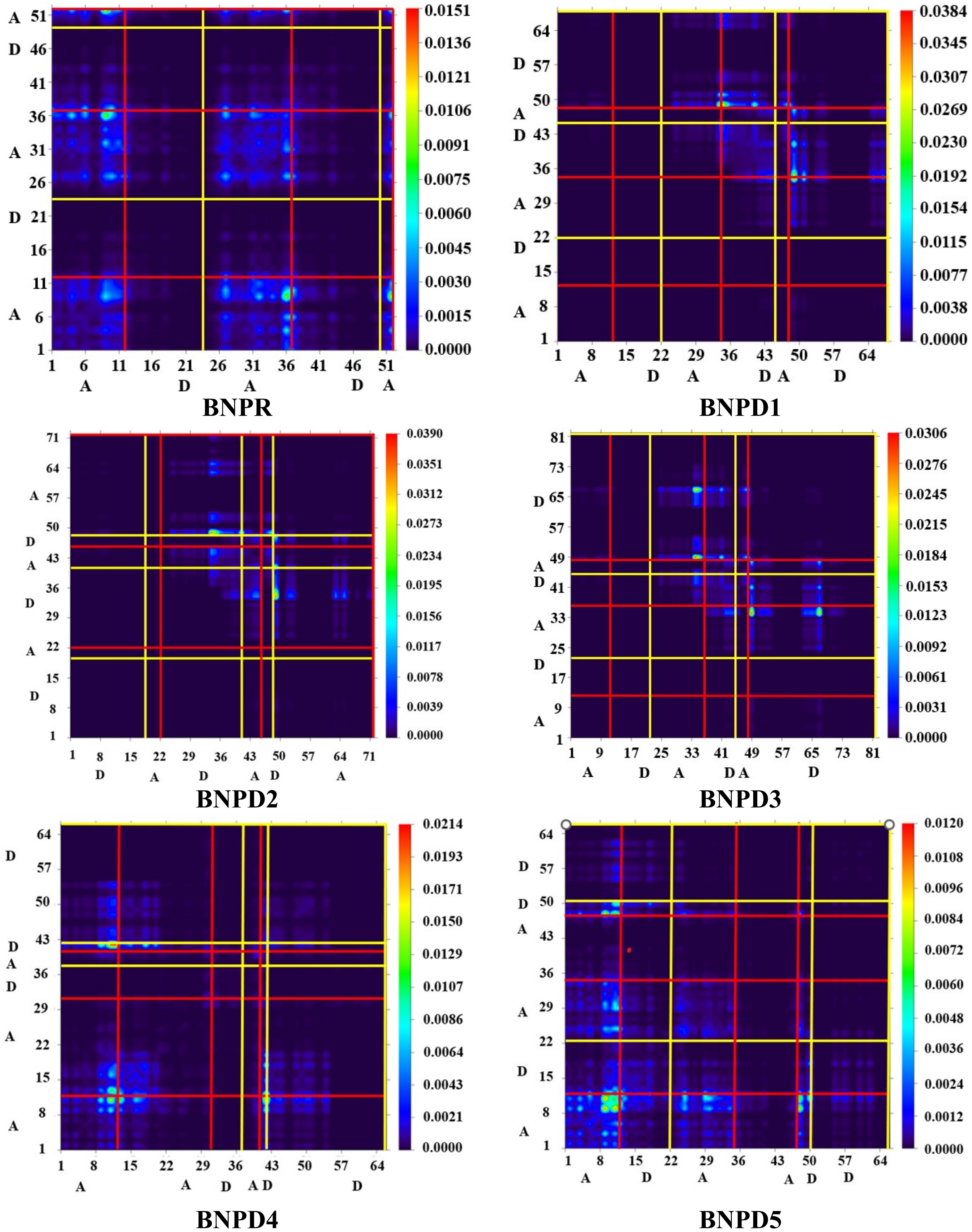

Figure S5: TDM representations of **BNPR** and **BNPD1-BNPD5** chromophores.

$$\text{RMSE} = \sqrt{\frac{1}{n}\sum_{i=1}^{n}(\hat{y}i - yi)^2} \quad \text{(S1)}$$

$$\text{MAE} = \frac{1}{n}\sum_{i=1}^{n}|\hat{y}i - yi| \quad \text{(S2)}$$

$$R^2 = 1 - \frac{\sum_{i=1}^{n}(\hat{y}i - yi)^2}{\sum_{i=1}^{n}(\hat{y}i - \bar{y}i)^2} \quad \text{(S3)}$$

$$\text{F1} = 2 \times \frac{Precision \times Recall}{Precision + Recall} \quad \text{(S4)}$$

$$IP = -E_{HOMO} \quad \text{(S5)}$$

$$EA = -E_{LUMO} \quad \text{(S6)}$$

$$\eta = \frac{(IP - EA)}{2} \quad \text{(S7)}$$

$$X = \frac{(IP + EA)}{2} \quad \text{(S8)}$$

$$\omega = \frac{\mu^2}{2\eta} \quad \text{(S9)}$$

$$\mu = -\frac{(IP + EA)}{2} \quad \text{(S10)}$$

$$E_b = E_{gap} - E_{opt} \quad \text{(S11)}$$

Detailed Step-wise Algorithm of CatBoost Model:

CatBoost is an effective gradient-boosting algorithm that is used as a machine learning tool, especially in problems with structured input. It builds on the principle of gradient boosting, which is a technique of ensemble learning. CatBoost applies regularization to avoid overfitting. The methods apply limitations to the training process in order to prevent the model from getting too complex and to fit the training data too well. Regularization is useful in generalizing the model

and strengthening it against invisible data. The algorithm will make use of gradient descent to construct the ensemble of trees by minimizing the loss function. In every iteration, it estimates the negative gradient of the loss function with respect to the existing predictions and estimates a new tree based on the negative gradient. Learning rate defines the step taken in the descent of the gradient. The process is continued until a specific number of trees has been added or a convergence criterion is reached. CatBoost takes the combination of the predictions of all trees in the ensemble when making predictions. Such a combination of predictions leads to very accurate and reliable models.

CatBoost may be expressed in the following way: Given a training dataset of N samples and M features, such that each sample is represented as (x_i, y_i), where x_i is a vector of M features, and y_i is the target variable. CatBoost is expected to learn a function $F(x)$, when applied to x, that predicts the target variable y.

$$F(x) = F_0(x) + \sum_{m=1}^{M} \sum_{i=1}^{N} f_m(x_i) \tag{S13}$$

While,

$F(x)$ is the total prediction function that CatBoost is supposed to train. It is given an input vector x, and it makes a prediction of the target variable y.

$F_0(x)$ denotes the initial guess or the initial prediction. It is usually fixed as an average of the target variable in the training data. This is the general average behavior of the target variable.

$\Sigma_{m=1}^{M}$ is the summation of an ensemble of trees. M refers to the size of the ensemble.

$\Sigma_{i=1}^{N}$ denotes the summation of the training samples. N refers to the overall number of training samples.

$F_m(x_i)$ denotes forecast of the m-th tree of the i-th training sample.

Every tree in the ensemble makes its prediction of each training sample, contributing to the overall prediction. It has been written as follows: the overall prediction $F(x)$ equals $F_0(x)$ along with predictions of every tree $F_m(x_i)$ of every training sample. This summation is done in all the trees (m) and all training samples (https://www.geeksforgeeks.org/machine-learning/catboost-algorithms).
